# Mapping yield and yield-related traits using diverse common bean germplasm

**DOI:** 10.3389/fgene.2023.1246904

**Published:** 2024-01-03

**Authors:** Yarmilla Reinprecht, Lyndsay Schram, Gregory E. Perry, Emily Morneau, Thomas H. Smith, K. Peter Pauls

**Affiliations:** ^1^ Department of Plant Agriculture, University of Guelph, Guelph, ON, Canada; ^2^ Harrow Research and Development Centre, Agriculture and Agri-Food Canada, Harrow, ON, Canada

**Keywords:** common bean (bean), yield, common bacterial blight, molecular markers, GWAS, marker validation, quantitative trait locus/loci (QTL) confirmation

## Abstract

Common bean (bean) is one of the most important legume crops, and mapping genes for yield and yield-related traits is essential for its improvement. However, yield is a complex trait that is typically controlled by many loci in crop genomes. The objective of this research was to identify regions in the bean genome associated with yield and a number of yield-related traits using a collection of 121 diverse bean genotypes with different yields. The beans were evaluated in replicated trials at two locations, over two years. Significant variation among genotypes was identified for all traits analyzed in the four environments. The collection was genotyped with the BARCBean6K_3 chip (5,398 SNPs), two yield/antiyield gene-based markers, and seven markers previously associated with resistance to common bacterial blight (CBB), including a Niemann–Pick polymorphism (NPP) gene-based marker. Over 90% of the single-nucleotide polymorphisms (SNPs) were polymorphic and separated the panel into two main groups of small-seeded and large-seeded beans, reflecting their Mesoamerican and Andean origins. Thirty-nine significant marker-trait associations (MTAs) were identified between 31 SNPs and 15 analyzed traits on all 11 bean chromosomes. Some of these MTAs confirmed genome regions previously associated with the yield and yield-related traits in bean, but a number of associations were not reported previously, especially those with derived traits. Over 600 candidate genes with different functional annotations were identified for the analyzed traits in the 200-Kb region centered on significant SNPs. Fourteen SNPs were identified within the gene model sequences, and five additional SNPs significantly associated with five different traits were located at less than 0.6 Kb from the candidate genes. The work confirmed associations between two yield/antiyield gene-based markers (AYD1m and AYD2m) on chromosome Pv09 with yield and identified their association with a number of yield-related traits, including seed weight. The results also confirmed the usefulness of the NPP marker in screening for CBB resistance. Since disease resistance and yield measurements are environmentally dependent and labor-intensive, the three gene-based markers (CBB- and two yield-related) and quantitative trait loci (QTL) that were validated in this work may be useful tools for simplifying and accelerating the selection of high-yielding and CBB-resistant bean cultivars.

## 1 Introduction

Common (dry) bean (*Phaseolus vulgaris* L.) (bean) is the most important legume crop for direct human consumption. Beans contain high levels of dietary fibers, proteins, vitamins, and minerals and have moderate levels of calories because of low levels of fats and high levels of resistant starch (Duranti, 2006; Hefni et al., 2010). *P. vulgaris* is of Mesoamerican origin, with the common ancestor most likely located in Mexico (Bitocchi et al., 2012). It was domesticated from two geographically isolated and genetically differentiated gene pools, which diverged from a common wild population more than 100,000 years ago (Gepts, 1988). The two gene pools are subsequently subdivided into races with different morphological, phenological, agronomic, biochemical, and molecular traits (Singh et al., 1991; Beebe et al., 2000; Singh, 2001). The small-seeded Mesoamerican gene pool is divided into three races: Mesoamerica [white (navy) and black market classes], Durango/Jalisco (pinto, pink, great northern, and small red), and Guatemala (climbing beans). The large-seeded Andean gene pool is split into Nueva Granada (white kidney, light red kidney, dark red kidney, and cranberry market classes), Peru, and Chile races. There is strong interest in characterizing and increasing genetic diversity in bean breeding germplasm since the founding populations for domestication only captured a small portion of the available diversity. This is particularly true for beans of Andean origin that are used for breeding (Navabi et al., 2014).

The development of high-yielding cultivars is the main goal of bean breeding programs in North America and Europe. The University of Guelph bean breeding program is focused on breeding high-yielding cultivars within navy, black, kidney, and cranberry market classes that are suited for production in the Southern Ontario environment. In addition, the cultivars must meet consumer preferences in various markets and maintain quality traits of each market class.

In general, small-seeded Mesoamerican genotypes are higher yielding than large-seeded Andean beans (Beebe, 2012). Yield and yield-related components, including seed weight, are complex, quantitatively inherited traits highly influenced by the environment (Singh, 2001), but specific loci conditioning yield have been located throughout the genome (Checa and Blair, 2008; Diaz et al., 2017; Oladzad et al., 2019). Understanding the relationships between yield and its components is important for devising effective selection criteria and breeding strategies. Selection for yield component traits has been used to indirectly improve yield and develop improved bean cultivars.

Common bacterial blight (CBB), caused by the bacteria *Xanthomonas citri* pv. *fuscans* (Xcf) and *Xanthomonas phaseoli* pv. *phaseoli* (Xpp), is among the most destructive diseases of Canadian beans. All aboveground plant parts can be infected and show symptoms such as dry and brown necrotic lesions surrounded by a narrow yellow halo on leaves or yellow and then brown spots on seeds at the areas of infection (Chen et al., 2021). With warm temperatures and severe infection pressure, the whole plant may die. However, infected plants and seeds may also be symptomless and pass on the infection to the next generation. Under environmental conditions favorable for disease development, substantial yield loss and reductions in seed quality have been reported in susceptible cultivars (Shi et al., 2011; Durham et al., 2013; Boersma et al., 2015). There are no effective chemical controls for the disease, so the industry relies on crop production from pedigreed seed grown in CBB-free environments, which adds additional expenses to the bean producers. Therefore, growing cultivars with resistance to CBB is the best disease management strategy in beans. The navy bean cultivar OAC Rex was the first CBB-resistant bean cultivar released in Ontario. It was developed at the University of Guelph from an interspecific cross that transferred CBB resistance from *Phaseolus acutifolius* into *P. vulgaris* (Michaels et al., 2006).

Numerous loci associated with yield and yield-related traits have been identified and mapped on all 11 bean chromosomes. The majority of these quantitative trait loci (QTL) were identified by linkage mapping with various biparental populations (Tar’an et al., 2002; Beattie et al., 2003; Blair et al., 2006; Wright and Kelly, 2011; Blair et al., 2012a; Mukeshimana et al., 2014). Association mapping [or genome-wide association study (GWAS)] approaches were also used to identify genome locations associated with yield by using collections of diverse bean germplasm (Galeano et al., 2012; Kamfwa et al., 2015). The two mapping techniques complement each other and were successfully used to map over 20 QTL for CBB resistance on all 11 bean chromosomes (Miklas et al., 2000b; Yu et al., 2000b; Miklas et al., 2003; Miklas et al., 2006; Viteri et al., 2015; Zhu et al., 2016). Often, QTL for yield and yield-related traits such as seed weight, flowering, maturity, and CBB resistance occur on the same chromosome (Mukeshimana et al., 2014; Berny Mier Y Teran et al., 2019).

Based on linkage disequilibrium [(LD) “the nonrandom association of alleles at different loci” (Flint-Garcia et al., 2003)], GWASs use collections of diverse germplasm and, thus, avoid the need for population development. They offer high resolution in localizing QTL because they include genotypes that are separated by many historical recombination events. However, the approach may result in spurious identifications of associations due to population size and structure and inadequate assessment of rare alleles and requires high marker density to detect significant QTL. In addition, factors like heritability, genetic architecture of the trait, and the statistical model used may also affect the detection ability of the GWAS. Sequencing of the bean genome (Schmutz et al., 2014) and identification of numerous single-nucleotide polymorphisms (SNPs) have allowed the development of platforms such as BeadChip (Hyten et al., 2010; Song et al., 2015). They have been used in both linkage mapping and association approaches to identify SNPs associated with yield and a number of yield-related traits in various locations of the bean genome (Trapp et al., 2015; Hoyos-Villegas et al., 2016; Geravandi et al., 2020).

The use of multiparent advanced generation intercross (MAGIC) populations can overcome the limitations of both QTL and GWAS approaches (Cavanagh et al., 2008). Diaz et al. (2020) identified a major QTL for yield and phenological traits on chromosome Pv01 by using a MAGIC population from eight Mesoamerican breeding lines using genotyping by sequencing (GBS) and whole-genome sequencing (WGS) data for GWAS and linkage mapping.

Comparative studies have revealed extensive preservation of genome order, or synteny, among species (McConnell et al., 2010). Recently, a meta-analysis that combined data from different QTL and/or GWASs (Goffinet and Gerber, 2000) identified the genomic regions in the current *P. vulgaris* v2.1 sequence map that were most consistently associated with yield and yield components in bean (Arriagada et al., 2023; Izquierdo et al., 2023). A similar approach was used to identify meta-QTL (MQTL) for major diseases in bean, including CBB (Rahmanzadeh et al., 2022).

The objective of the present research was to use association mapping to identify loci for yield and yield-related traits with a diverse collection of 121 bean genotypes field-evaluated in four Ontario locations/year environments and genotyped with the BeadChip SNP array and a number of gel-based markers previously associated with the yield and CBB resistance. The work not only validated some of the QTL identified previously with different germplasm/markers/analyses but also identified new regions associated with the yield and yield-related traits.

## 2 Materials and methods

### 2.1 Assembly of the yield/antiyield association mapping (AYD_AM) panel

An association mapping panel was assembled from 121 diverse bean genotypes, used or created by the University of Guelph bean breeding program, which represented a range of previously measured yields (1,618–4,107 kg ha^−1^). The selected genotypes belonged to diverse market classes, gene pools, and years of release. The panel (AYD_AM) consisted of 42 genotypes from the University of Guelph bean registration trials (14 large-seeded colored beans and 28 navy beans), 38 historical cultivars/lines, and 41 genotypes from a previous phenyl propanoid study (Supplementary Table S1, Supplementary Figure S1). In general, the recently released, modern cultivars yielded more than the landraces or cultivars developed in the 1960s (Supplementary Figure S2).

The panel contained predominantly small-seeded Mesoamerican genotypes (70.2%). These accessions (85) consisted of navy (57), black (12), pinto (9), small red (4), and great northern (1) market classes. In addition, there were 34 large-seeded Andean accessions, mainly from cranberry (10), dark red kidney (8), light-red kidney (5), white kidney (3), and yellow (3) market classes. Based on the seed phenotype, nine genotypes did not belong to any of the bean market classes typically grown in North America. Two genotypes were from the Bat93 (Mesoamerican) x Jalo EEP558 (Andean) cross (Supplementary Table S1, Supplementary Figure S1).

### 2.2 Phenotyping

#### 2.2.1 Field evaluation

The AYD_AM panel was evaluated for yield and a number of yield-related traits at the University of Guelph, Elora [ERS; 43°39′N, 80°25′W; 2,680 crop heat units (CPU) (Brown, 1993), London loam soil] and Woodstock, Ontario (WRS; 43°08′N, 80°46′W; 2,890 CHU, Guelph loam soil [Voisey, 1971]) research stations in 2015 and 2016. In both years, the previous crop was alfalfa at the ERS. At the WRS, the first year planting followed winter wheat and the second year (2016) planting followed corn. The 121 entry trials were conducted using a square (11 × 11) lattice design with four replications. The entries were planted in four-row plots with a distance of 0.50 m between plots. The rows were 1.9 m long and spaced 0.36 m apart. The experiments were machine-planted on 28 May 2015 at the WRS and on 22 June 2015 at the ERS. The plots were combine-harvested on 24 September 2015 at the WRS and on 26 October 2015 at the ERS. In 2016, the experiments were machine-planted on 27 May 2016 at the WRS and on 3 June 2016 at the ERS. The plots were combine-harvested on 14 September 2016 at the WRS and on 4 October 2016 at the ERS. Standardized cultural practices were performed in all trials as needed.

##### 2.2.1.1 Conventional in-field data collection

Flowering (days to flowering, DF) was measured as the number of days from planting to 50% of the plants in the plot with at least one flower fully opened. Maturity (days to maturity, DM) was calculated as the number of days from planting to 95% of the plants in the plot at physiological maturity. Plant height (PH) in centimeter was determined after flowering as the height of the plant from the soil surface to the tip of the main stem. Seed weight (SW) was measured as the weight of 100 randomly selected threshed seeds (g) and adjusted to 18% moisture content. Yield (YD) was determined as the seed weight measured for each plot in kg ha^-1^ and adjusted to 18% moisture content. Harvestability (HR) was determined as the standability of plants in a plot at maturity (in 2016 only) using a scale of 1–5 (1, erect plants suitable for machine harvesting; 5, prostrate plants not suitable for combine harvesting).

##### 2.2.1.2 Derived traits

The reproductive period (RP, days) was calculated as the difference between days to maturity and days to flowering (RP = DM–DF; Scully and Wallace, 1990; Orf et al., 1999). The seed growth rate (SGR) was estimated as the ratio between the measured yield and derived reproductive period (SGR = YD/RP) and expressed in kg ha^-1^ day^-1^ (Scully and Wallace, 1990). Yield gain per day (YGD) was estimated as the ratio between yield and maturity (YGD = YD/DM) and expressed in kg day^-1^ ha^-1^ (Scully and Wallace, 1990). Yield per unit of height (YDH) was calculated as the yield-to-height ratio (YDH = YD/HT) and expressed in kg ha^-1^ cm^-1^ (Orf et al., 1999). High YDH values are obtained from short plants with high yields. Seed number (SN) was estimated as the yield-to-seed weight ratio (SN = YD/SW) and expressed as seed number x 10^6^ seeds ha^-1^ (Orf et al., 1999). Yield per unit of harvestability was calculated as yield divided by harvestability score (YDHR = YD/HR) and expressed in kg ha^-1^. High YDHR values are obtained from plants with high yields and suitable for combine harvesting (low harvestability scores).

##### 2.2.1.3 CBB inoculation and rating

Plants were grown in the CBB nursery at the Agriculture and Agri-Food Canada Research and Development Centre in Harrow, Ontario, in 2015 and 2016. Entries were planted following a lattice design, with two replications, into hill plots, which contained seven plants. The bacterial inoculum was prepared with two strains of *X. campestris* pv. *phaseoli* (strains 18 and 98) and two strains of *X. fuscans* subsp. *fuscans* (strains 12 and 118), following a modified protocol outlined in Yu et al. (2000a). Inoculation was performed twice, five and again six weeks after after planting. Plots were inoculated with a bacterial culture suspended in deionized water using a high-pressure sprayer which injures the leaf canopy as it sprays the plant with the inoculum, thus allowing for bacterial infection of the leaf tissue. The sprayer was pulled at a rate of 3 km h^-1^, and the inoculum was sprayed at a pressure of 200 psi. The plots were irrigated twice a week from the point of first inoculation until rating to maintain high humidity and promote bacterial growth.

The rating for CBB disease severity was conducted two weeks after the second inoculation and 10 days later. Overall disease severity across plants in the hill plot and the lesion leaf cover on each plant were rated. The total active lesion cover, determined as a percentage, was rated on a scale from 0 to 5, where plots with no active lesions were rated 0, 1%–10% infection was rated 1, 11%–30% infected leaf area was rated 2, 31%–50% infected leaf area was rated 3, 51%–80% infected leaf area was rated 4; and >80% of leaf area under active infection was rated 5. The area under the disease progress curve (AUDPC) was calculated using the following equation (Madden et al., 2007):

$$AUDPC=\sum_{i=1}^{n-1}\frac{y_{i}+y_{i+1}}{2}\left(t_{i+1}-t_{i}\right),$$

where

“y_i_” and “y_i+1_“ are assessments of disease severity ratings “i” and the next “i+1”, respectively; “t_i_” and “t_i+1_“ are time intervals (days) between evaluations; and “n” is the total number of evaluations.

Higher AUDPC values indicate higher disease intensity (susceptibility). In this study, the two CBB ratings and the AUDPC value were treated as separate traits (CBB_R1, CBB_R2, and CBB_AUDPC).

#### 2.2.2 Weather conditions during bean growing seasons

Daily weather data for the two locations (Elora and Woodstock) in the two experimental years (2015 and 2016) were collected at the nearest weather stations (available at: http://climate.weather.gc.ca/climateData/dailydata_e.html).

#### 2.2.3 Field data analysis

Analysis of variance was performed using the GLIMMIX procedure to fit the generalized linear mixed model (GLMM) for lattice or randomized complete block design (RCBD) in Statistical Analysis System (SAS) v.9.4 software (SAS Institute, 2013). Data were analyzed separately for each environment (location and year) and combined across locations and years. The genotype was considered a fixed effect, while the environment, genotype x environment, and all other effects were estimated as random effects. A residual analysis was used to test the homogeneity of error variances before pooling data for combined analyses. Studentized residuals were plotted against the predicted values and visually inspected to check if they were randomly and independently distributed. The Shapiro–Wilk test was performed on the residuals in the UNIVARIATE procedure to test their normality. A few data produced outlier residuals, and they were removed from the analysis.

The least square means (LS means) for each trait was used to calculate the pair-wise Spearman’s rank correlation for all traits using the CORR Spearman procedure in SAS. Significant relationships between a number of traits were identified, which suggested that multivariate principal component analysis (PCA) would be a suitable method for data reduction. The PRINCOMP procedure was used in SAS to determine the principal component (PC) values and estimate the proportion of variance explained by each PC. A scree plot of the eigenvalues of PCs was used to determine the number of PCs to keep in a PCA. Genotype x trait (GT) biplots were plotted in Microsoft Excel.

Multi-Environment Trait Analysis with R (Meta-R) for Windows version 6.04 software (Alvarado et al., 2020) was used to estimate the best linear and unbiased estimators (BLUEs) and the best linear and unbiased predictors (BLUPs), the variance–covariance parameters, the genetic variance, and the broad-sense heritability. BLUE estimates averaged over four environments were used in the GWAS analyses.

### 2.3 Genotyping

#### 2.3.1 BeadChip and gene-based markers

Genomic DNA was isolated from 121 AYD_AM bean genotypes using a DNeasy plant mini kit (QIAGEN, Canada) according to the manufacturer’s instructions. Genotyping was performed using the bean BARCBean6K_3 BeadChip (Hyten et al., 2010; Song et al., 2015) containing 5,398 SNPs. Chip genotyping was done using the Illumina^®^ Infinium High-Density (HD) Assay at the McGill University and Génome Québec Innovation Centre (Montreal, Canada). SNP calling was done using GenomeStudio software. The panel was also screened with the two yield/antiyield gene-specific temperature switch PCR (TSP) markers (Reinprecht et al., 2021), a number of sequence characterized amplified region (SCAR) markers previously associated with CBB resistance in bean (Miklas, 2005), and the gene-based Niemann–Pick polymorphism (NPP) marker that was developed to screen bean for CBB resistance (Morneau, 2019). All additional markers were gel-based (Supplementary Table S2).

#### 2.3.2 Data filtering

Only SNPs with known physical positions in the bean v2.1 genome sequence were used in mapping. The SNPs (5,300 with known genome positions) were pre-processed using Trait Analysis by aSSociation, Evolution, and Linkage (TASSEL) v. 5.2.85 software (Bradbury et al., 2007) using the following parameters: missingness 10% (missing SNPs per genotype) and 7% (each SNP present in 113 out of 121 genotypes), 5% both minor allele frequency (MAF) and heterozygosity, and minor alleles removed. Filtering was also done using PLINK 1.9 software (Purcell et al., 2007). Four genotypes exceeded heterozygosity (5%) and missingness (10%) thresholds [heterozygosity: XAN 159 (11.5%) and ICA Pijao (11.3%); missingness: Montcalm (9.9%) and ACUG 14–7 (5.6%)]. However, their removal did not improve mapping results, and they were retained in the panel. Because LD pruning removed many SNPs from the dataset (1,827 SNPs remained), creating large regions with no SNP representation, and because the missing data level was only 0.5%, neither LD pruning nor imputation was used. Implementation of LD pruning and imputation did not change the GWAS results. Therefore, filtered marker data with 4,485 SNPs (84.6% of initial SNPs with a proportion of missingness of 0.00549, a proportion of heterozygosity of 0.00435, and an average MAF of 0.2846) and 121 genotypes were used for the GWAS analyses.

#### 2.3.3 Genetic diversity and LD

The level of genetic diversity in the AYD_AM panel was assessed using the filtered SNP dataset (4,485 SNPs) in Tassel 5. Within a basic diversity analysis, nucleotide diversity (average pairwise divergence between all possible pairs of genotypes in a collection, π; Nei and Li, 1979), estimated mutation rate (θ; Watterson, 1975), and Tajima’s D (Tajima, 1989) were calculated at the genome level, as well as for each chromosome, using sliding windows including 50 sites (“window size”) and steps (“how far the window jumps to do the next analysis”) of 200 sites.

LD was estimated in Tassel 5 in various ways, including “sliding window LD” analysis, which calculates LD for sites within a window of sites surrounding the current site, at a setting of 50 bp, “LD Window Size”, which determines the width of the window on one side of the current site, and “Full Matrix LD”, which calculates LD for every combination of sites in the alignment. For all methods, heterozygotes were treated as missing data. The LD results were visualized in R (https://github.com/mohsinali1990/My_scripts/blob/main/LD%20decay%20Plot%20from%20TASSEL%20LDoutput.R).

#### 2.3.4 Population structure

The initial analysis of population structure was performed with 20 evenly spaced SNPs per chromosome (220 in total) using a Bayesian clustering model, implemented in Structure 2.3.4 software (Pritchard et al., 2000), with the following parameters: 100,000 burn-ins, 200,000 Markov chain Monte Carlo (MCMC) replications after burn-in, admixture ancestry model, and correlated allele frequency model, with the number of populations (K) set from 1 to 10 and with 15 iterations. The population structure was also determined with the whole filtered dataset (4,485 SNPs) with 5,000 burn-ins, 50,000 MCMC replications after burn-in, and using admixture ancestry models and correlated allele frequency models. Five runs were performed for each number of populations (K) set from 1 to 10. The best (true) K was identified by L(K) and delta K methods. Structure Harvester (Earl and von Holdt, 2012; http://taylor0.biology.ucla.edu/structureHarvester/) was used to visualize the structure output and implement Evanno’s method (Evanno et al., 2005). The cutoff probability for assigning genotypes to a cluster was 0.60 or above. The population structure was plotted using Structure Plot v2 software (Ramasamy et al., 2014).

PCA and evolutionary relatedness among the AYD_AM panel genotypes were assessed with the filtered dataset (4,485 SNPs) in Tassel 5. Five PCs were calculated, and the first three PCs were visualized in CurlyWhirly (https://ics.hutton.ac.uk/curlywhirly).

Evolutionary relationships among genotypes were inferred using the neighbor-joining method based on the modified Euclidean distance (“where a homozygote is 100% similar to itself, but a heterozygote is only 50% similar to itself”) in Tassel 5. Dendrograms were visualized using Dendroscope 3 software (Huson et al., 2007).

#### 2.3.5 AYD_AM GWAS

Association mapping was performed with the filtered SNPs using Genomic Association and Prediction Integration Tool (GAPIT) v3 software (Lipka et al., 2012; Wang and Zhang, 2021) with multiple methods in the GWAS, including the General Linear Model (GLM), Mixed Linear Model (MLM), Multiple Locus Mixed linear Model (MLMM), Fixed and random model Circulating Probability Unification (FarmCPU), and Bayesian-information and Linkage-disequilibrium Iteratively Nested Keyway (BLINK) with 2–5 PCs and the kinship (VanRaden, 2008) (both calculated in GAPIT) to control for population structure and relatedness. The suitability of the model(s) to account for population structure was assessed using quantile–quantile (Q-Q) plots. Significance levels were established using the Bonferroni corrections at *p* < .05 (0.05/4,485 = 1.11483^–5^) (calculated in GAPIT). Since BLINK does not report *R* ^2^ for identified SNPs, the code “Random.model = TRUE” was added to calculate the phenotypic variance explained (PVE). Manhattan plots were visualized using SRplot (http://www.bioinformatics.com.cn/srplot).

#### 2.3.6 Comparison with previously identified QTL and candidate gene investigation

SNP sequences (121 bp) were BLASTed against the bean genome v2.1 sequence to identify their physical positions. Chromosome positions and flanking markers for QTL associated with the current study yield and yield-related traits were retrieved from the Legume Information System (https://legumeinfo.org/), SoyBase (https://soybase.org/), and Pulse Crop Database (http://pulsedb.org). A literature search was performed to identify previously reported QTL and genes, not present in databases, which co-localize with the regions significantly associated with the traits analyzed in the current study. The approximate (tentative) positions of the QTL on the bean physical map were identified *in silico* by BLASTing sequences of QTL flanking markers against the bean v2.1 genome sequence in Phytozome v13 (Goodstein et al., 2012).

To identify candidate genes, a 200-Kb region centered on each significant marker was searched using the JBrowse tool in the *P. vulgaris* v2.1 (*P. vulgaris* accession G19833 genome assembly v2.1) available in the Phytozome and Legume Information System (Dash et al., 2016). The annotation of the candidate genes was obtained from the “Pvulgaris_442_v2.1.annotation_info.txt” (Phytozome) and/or “G19833.gnm2.ann1.PB8d” (https://www.legumeinfo.org/collections/phaseolus/) files. Publications were also examined for candidate genes identified previously.

The SNP-based sequence and MTA/QTL alignment maps were drawn using MapChart (Voorrips, 2002).

#### 2.3.7 Single-marker analysis

The association of yield/antiyield gene-based TSP markers and CBB-related markers with yield and yield-related traits was analyzed with the PROC REG and PROC CORR Spearman procedures in SAS using LS means values for traits and marker scores. Adjusted *R* ^2^ from regression analysis was used as a measure of the trait’s phenotypic variability explained by the marker.

## 3 Results

### 3.1 Phenotypic evaluation of the AYD_AM collection of beans

#### 3.1.1 Environmental conditions

The weather conditions during the growing season at the ERS varied over the two experimental years (Supplementary Figure S3). However, because of incomplete weather data for the WRS site, direct comparisons among the location/years were not possible over the bean growing seasons. Nevertheless, in both years, the warmer temperatures at the WRS resulted in earlier maturity values at that location. At the WRS, the beans were in the field for 119 days in 2015 and 110 days in 2016, compared to 126 days in 2015 and 123 days in 2016 for the beans grown at the ERS.

#### 3.1.2 Variability of analyzed traits and heritability estimates

Random variation effects due to environment, genotype × environment interaction, and block were successfully removed by the REML estimation of GLIMMIX (Supplementary Table S3). Significant variability among the 121 bean genotypes (fixed effect) was identified for all analyzed traits (Supplementary Table S4). The averaged yield (four location/year environments) varied from 965 kg ha^-1^ (Othello) to 2,837 kg ha^-1^ (Lariat). Both of these cultivars are pinto beans (small-seeded Mesoamerican). The variability of seed weight was even more pronounced because the panel included genotypes belonging to both gene pools, ranging from 17.0 g (navy bean OAC Spark, Mesoamerican) to 68.4 g (PI 598312, Andean bean). The best resistance against CBB was identified in a breeding line ACUG 13-1, in both ratings (0.88 ± 0.681 and 1.50 ± 0.361), as well as in AUDPC (11.00 ± 3.319). Cultivar Island (pinto bean) was the most susceptible to CBB in rating 1 (4.13 ± 0.681) and AUDPC (39.00 ± 3.319), while AC Elk (light red kidney) had the highest CBB rating 2 (5.00 ± 0.361). Estimates of broad-sense mean-based heritability varied from 47.2% (CBB_R1) to 98.3% (SW) for the 15 analyzed traits (Supplementary Table S5). High percentages of broad-sense heritability were estimated for phenological traits (DF at 95.9% and DM at 93.9%) and plant height at 85.5%. The yield exhibited a moderately lower percentage (77.2%), followed by harvestability (68.7%).

#### 3.1.3 Relationships among traits

Spearman’s correlational analysis using LS means (N = 121) combined over four environments identified significant correlations among the evaluated traits (Table 1). The majority of the relationships were confirmed for each location/year environment separately (Supplementary Table S6). Significant positive associations were identified between the four environments for most of the traits, except for the derived traits YGD (ERS16_WRS15), SGR (ERS16_WRS15 and WRS15_WRS16), and YDH (ERS16_WRS15) (Supplementary Table S6). The results of a combined analysis indicated an inverse relationship between yield and seed weight [r (120) = −.229, *p* = .0115] and yield and CBB susceptibility, both ratings and AUDPC [CBB_AUDPC, r (120) = −.309, *p* = .0006]. There were moderate-to-strong positive correlations between yield and most of the other evaluated traits, with correlation coefficients ranging from r (120) = .389 (*p* < .0001) with YDHR to r = .951 (*p* < .0001) with YGD, both yield-based derived traits. However, yield was not associated with harvestability averaged over four Ontario location/year environments. Seed weight was negatively correlated with the majority of traits, except with CBB, with which it was positively related but significant only with CBB_AUDPC [r (120) = −.220, *p* = .0155]. In contrast, flowering and maturity were positively correlated with all traits, except with CBB (Table 1).

**TABLE 1:** Spearman’s correlation coefficients among yield and yield-related traits in the AYD_AM panel of common beans averaged over four Ontario location/year environments (LS means, N = 121).

Trait^a^	SW	DF	DM	PH	HR	RP	YGD	SGR	YDH	SN	YDHR	CBB_R1	CBB_R2	CBB_AUDPC
**YD**	−.23*	.63***	.67***	.77***	.08	.48***	.95***	.89***	.89***	.73***	.39***	−.23*	−.36***	−.31***
**SW**		−.60***	−.48***	−.15	−.39***	−.22*	−.08	−.20*	−.28**	−.78***	.06	.14	.22*	.15
**DF**			.69***	.49***	.37***	.22*	.48***	.61***	.58***	.75***	.14	−.17	−.28**	−.22*
**DM**				.63***	.38***	.82**	.45***	.38***	.55***	.73***	.13	−.42***	−.57***	−.51***
**PH**					−.21*	.53***	.69***	.60***	.45***	.55***	.44***	−.32**	−.41***	−.38***
**HR**						.19*	.06	−.01	.28**	.33***	−.45***	−.06	−.12	−.08
**RP**							.29**	.10	.33**	.43***	.10	−.47***	−.57***	−.53***
**YGD**								.94***	.88***	.60***	.42***	−.15	−.26**	−.22*
**SGR**									.86***	.64***	.39***	−.07	−.18	−.13
**YDH**										.69***	.25**	−.11	−.24**	−.18*
**SN**											.20*	−.26**	−.41***	−.33***
**YDHR**												−.14	−.20*	−.17
**CBB_R1**													.74***	.93***
**CBB_R2**														.92***

^a^ Measured agronomic traits: YD, yield (kg ha^-1^); SW, seed weight (g); DF, flowering (days); DM, maturity (days); PH, plant height (cm); HR, harvestability (scale 1–5). Derived traits: RP, reproductive period (RP = DM–DF, days); YGD, yield gain per day (YGD = YD/DM, kg ha^-1^ day^-1^); SGR, seed growth rate (SGR = YD/RP, kg ha^-1^ day^-1^); YDH, yield per unit of height (YDH = YD/PH, kg ha^-1^ cm^-1^); SN, seed number (SN = YD/SW, seed number × 106 seeds ha^-1^); and YDHR, yield per unit of harvestability (YDHR = YD/HR, kg ha^-1^). Disease resistance: CBB_R1 = common bacterial blight (scale 0–5) first scoring; CBB_R2 = second CBB, scoring; and CBB_AUDPC = CBB, area under the disease progress curve.

*Indicates *p* < .05; ** indicates *p* < .01; *** indicates *p* < .001.

**FIGURE 1:**
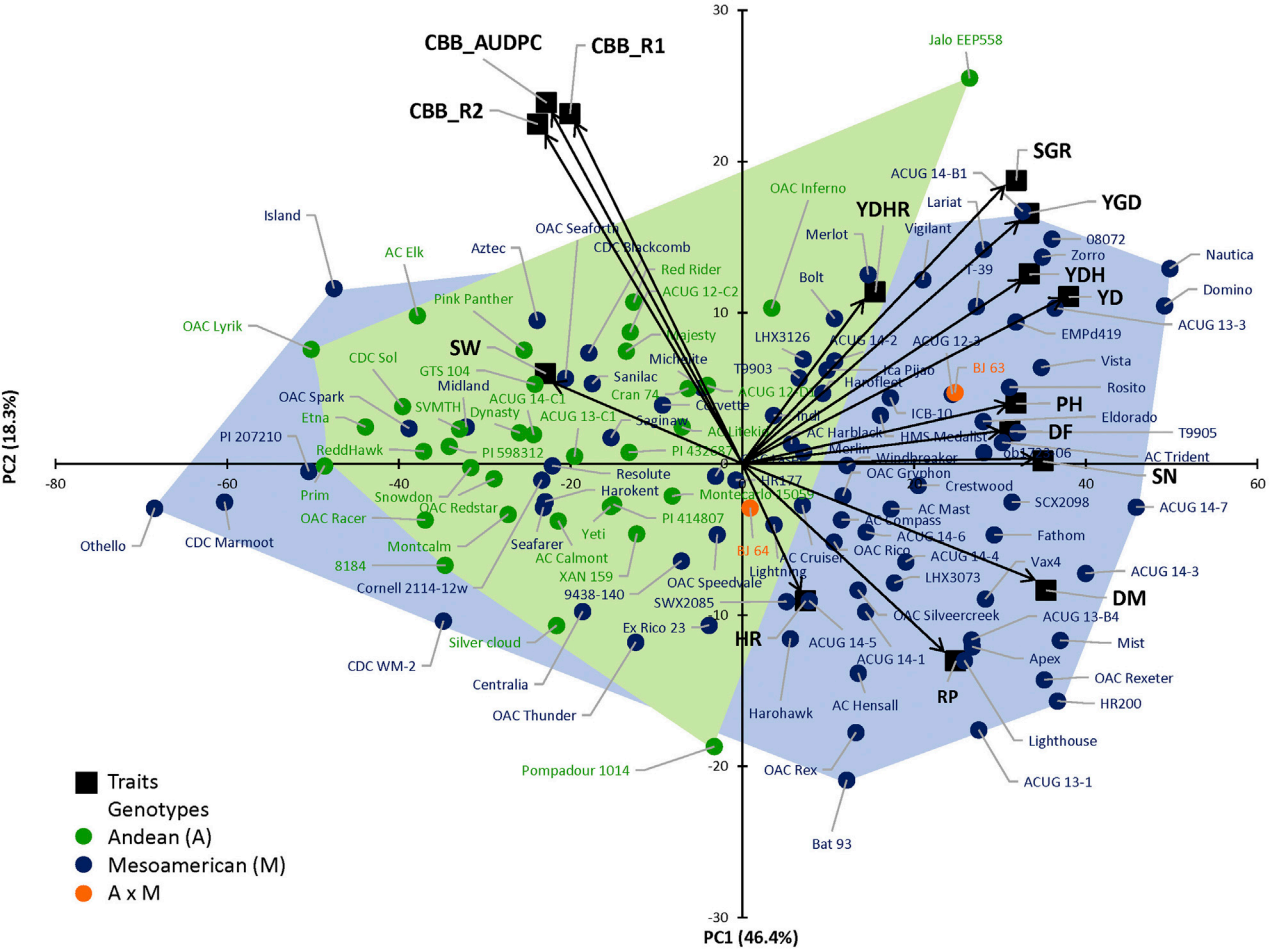
Principal component analysis (PCA) based on the correlation matrix for traits evaluated in the 121 AYD_AM collection of common beans averaged over four Ontario location/year environments. The first two principal components (PCs) explained 64.7% variability. Genotype–trait (GT) biplot analysis, where arrows represent original variables. The directions of arrows indicate the correlation between original variables and PCs, while their lengths describe the adherence of original data to PCs. Andean, Mesoamerican, and Andean*Mesoamerican individual distributions according to trait PC values. Individuals at the limits of the gene pool distributions are identified.

The complexity of the trait relationships, suggested by correlation analysis, was reduced by a PCA. Based on the Kaiser criterion (Kaiser, 1958), nine PCs were identified with eigenvalues >1, which cumulatively explained 99.5% of the variation for the evaluated traits (Table 2). The scree plot results indicated that three PCs should be kept in a PCA (Supplementary Figure S4). The first two PCs explained 64.7% of the variation observed in the data. Averaged over four location/year environments, PC1 had large positive associations with yield, flowering, maturity, and derived traits RP, YDH, and SN and was negatively associated with seed weight and CBB. PC2 had large positive associations with seed weight, CBB, and derived trait YDHR and was negatively associated with harvestability (Supplementary Table S7). Significant associations among traits were indicated by their groupings in a genotype-trait (GT) biplot. For example, seed weight and CBB were clustered together in a quadrant opposite to the negatively associated yield trait (Figure 1).

**TABLE 2:** Eigenvalues of the correlation matrix from the principal component analysis of the traits evaluated in the AYD_AM panel of common beans at four Ontario location/year environments.

Order	Eigenvalue	Percentage	Cumulative percentage
1	6.9655	46.44	46.44
2	2.7424	18.28	64.72
3	2.1615	14.41	79.13
4	1.0860	7.24	86.37
5	0.7666	5.11	91.48
6	0.5063	3.38	94.86
7	0.3451	2.30	97.16
8	0.1937	1.29	98.45
9	0.1604	1.07	99.52
10	0.0360	0.24	99.76
11	0.0201	0.13	99.89
12	0.0100	0.07	99.96
13	0.0035	0.02	99.98
14	0.0020	0.01	99.99
15	0.0008	0.01	100.00

There was no clear separation of the genotypes in the two gene pools, based on analyzed traits (Figure 1). However, the large-seeded Andean beans (94.1%) were grouped in the left quadrants of the plot (except OAC Inferno and Jalo EEP558). The small-seeded Mesoamerican genotypes filled the right quadrants of the plot (71.8%), but, interestingly, 24 genotypes were positioned in the left quadrants with the majority of the Andean beans.

### 3.2 GWAS of yield and yield-related traits in the AYD_AM bean panel

#### 3.2.1 SNP distribution

After removing SNPs with unknown genome positions (107) and pre-processing in Tassel, 84.6% of the SNPs (4,485) were used for analyses. Their occurrence ranged from 276 SNPs on chromosome Pv06 to 516 SNPs on chromosome Pv05, with the largest percentage of SNPs filtered out (approximately 20%) from chromosomes Pv08 and Pv11 (Supplementary Figures S5A–D). SNPs were denser denser toward chromosome ends. The genome-wide average marker density was one SNP every 114.5 Kb, varying from one SNP per 79.3 Kb on Pv05 to one SNP per 182.2 Kb on chromosome Pv03. The physical positions of the 4,485 SNPs are given in Supplementary Figure S6.

#### 3.2.2 Genetic diversity, population structure, and LD

Genome-wide nucleotide diversity (π, the average pairwise divergence among genotypes) was 0.387 per bp. There was significant variation in π along the bean genome, ranging from 0.349 on chromosome Pv09 to 0.411 per bp on chromosome Pv05. The average Watterson’s theta (θ, the expected number of polymorphic sites per nucleotide), which estimates the mutation rate in the panel, was 0.187 per bp. The θ values were similar among 11 bean chromosomes. The average Tajima’s D [which estimates the normalized measure of difference between the observed (π) and expected (θ) nucleotide diversity] was 3.603 per bp and varied along the bean genome from 2.872 on chromosome Pv09 to 3.962 per bp on chromosome Pv05 (Supplementary Table S8). Diversity attributes were also determined in older (50 cultivars released in or before 2000) and newer (71 cultivars released after 2000) cultivars, as well as in gene pools. The π values were similar between older (0.388) and newer (0.387) cultivars; θ was higher in older (0.223) cultivars than in the newer (0.207) cultivars, while the Tajima’s D was higher in newer cultivars (3.090) than in the older (2.624) cultivars. Mesoamerican genotypes had higher values for all three diversity indicators (π = 0.246, θ = 0.172, and Tajima’s D = 1.486) than the Andean gene pool (π = 0.145, θ = 0.143, and Tajima’s D = 0.056).

**FIGURE 2:**
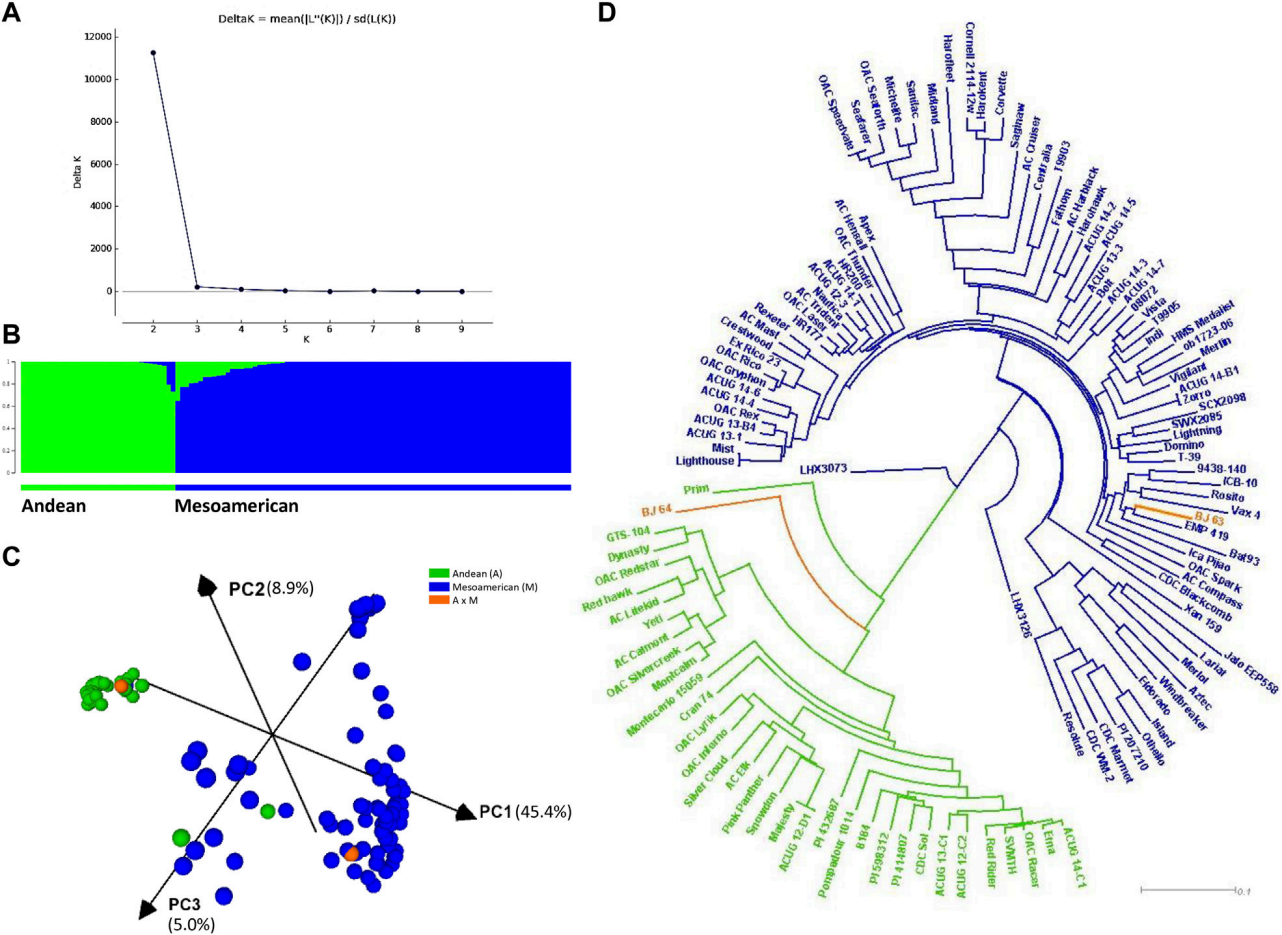
Population structure and relatedness of the AYD_AM panel of common beans performed with 4,485 SNPs. **(A)** Magnitude of delta K from STRUCTURE 2.3.4 analysis of 121 common bean genotypes. **(B)** Estimated population structure at K = 2 sorted by Q. Inferred populations are partitioned into colored segments representing the individual membership to the clusters. **(C)** Principal component analysis (PCA) performed in Tassel 5 and visualized in CurlyWhirly. **(D)** Neighbor-joining phylogenetic tree (created in Tassel 5 and visualized in Dendroscope).

Based on population structure analyses with 220 SNPs (Supplementary Figure S7) and 4,485 filtered SNPs (Figure 2A), the ΔK statistics indicated K = 2 as the best arrangement of genotypes, which suggested that the population could be divided into two subpopulations of small-seeded Mesoamerican (M, constituting 70.3%) and large-seeded Andean (A) beans. Establishing membership coefficient ≥0.60 criteria, 87 genotypes belonged to the Mesoamerican gene pool and 34 were identified as Andean beans. However, twenty-four genotypes within the Mesoamerican gene pool and eight genotypes within the Andean gene pool had some level of admixture (Figure 2B).

This subdivision was generally supported by the PCA and the phylogenetic analyses (4,485 SNPs). The first PC explained 45.4% of the variability, while the second and third PCs accounted for 8.9% and 5.0% of the variability, respectively (Figure 2C). With a few exceptions, the analysis divided the AYD_AM panel into two clusters according to the gene pools. A small-seeded navy bean cultivar OAC Silvercreek (0.996 A and 0.004 M) was grouped with the Andean beans, while two Andean genotypes [Jalo EEP558 and XAN 159 (both 0.773 M and 0.227 A)] were grouped with the Mesoamerican beans. Two genotypes from the Bat93 x Jalo EEP558 cross were positioned in different clusters. Line BJ 64 (0.793 A and 0.207 M) was grouped with the Andean beans, while BJ 63 (1.000 M) was grouped with the small-seeded Mesoamerican beans. The same grouping was identified in a dendrogram based on the identity by state (IBS) distance matrix (Figure 2D).

**FIGURE 3:**
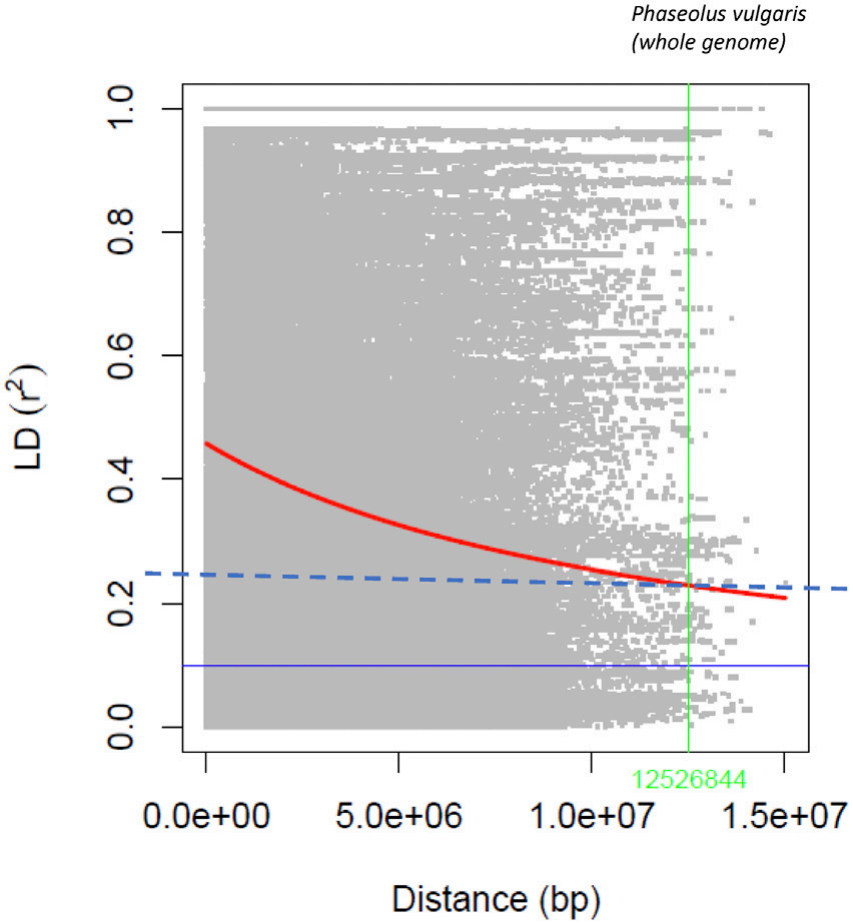
Linkage disequilibrium decay rate in the common bean genome evaluated with the 4,485 SNPs in the AYD_AM collection of 121 genotypes.

The statistic *r* ^2^, used to estimate LD between SNP pairs with a sliding window size of 50 SNPs, generally declined with increasing distance (Figure 3), and the rate of decay varied among chromosomes (Supplementary Figure S8). Using the *R* ^2^ = 0.2 value as a cutoff for LD, a long decay was determined at the genome level (12.53 Mb). The longest LD (13.82 Mb) was estimated for Pv04 and the shortest (1.70 Mb) for chromosome Pv06.

#### 3.2.3 Marker-trait associations

##### 3.2.3.1 GWAS model selection and significant association

Based on the Q-Q plots, BLINK was the most suitable method to identify significant marker-trait associations (MTAs) with the 4,485 SNPs in the AYD_AM collection of 121 bean genotypes using three PCs to control for population stratification, except for the derived trait RP (reproductive period), where five PCs were used (Supplementary Figures S9, S10).

**FIGURE 4:**
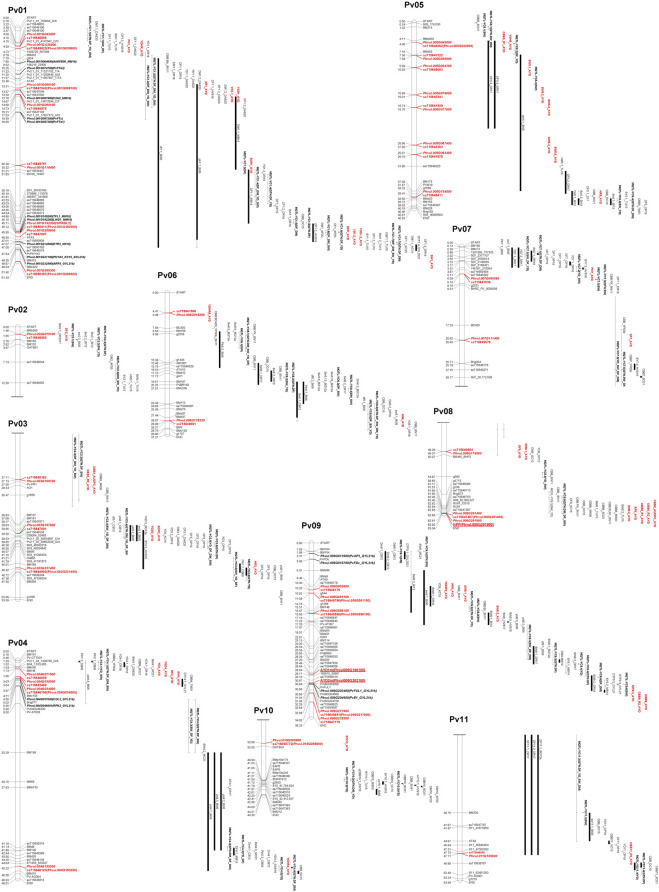
Physical map positions of significant MTAs identified in the current AYD_AM study and QTL reported previously for yield and yield-related traits in the common bean genome sequence (*Phaseolus vulgaris* v2.1). The MTAs (bold, red), yield/antiyield (AYD1m and AYD2m), and CBB (NPP) gene-based markers (red, bold, underlined) and previously published QTL are labeled to the right of the chromosomes. QTL tags consist of QTL name (original, if available) and first initials of last names of the first two authors and the year of publication [AP20 (Almeida et al., 2020), BG12a (Blair et al., 2012a), BI06 (Blair et al., 2006), CB08 (Checa and Blair, 2008), CB12 (Checa and Blair, 2012), DAS20 (Diaz et al., 2020), DC15 (Diaz Castro, 2015), DR17 (Diaz et al., 2017), DR18 (Diaz et al., 2018), GC12 (Galeano et al., 2012), GYL21 (González et al., 2021), HVS16 (Hoyos-Villegas et al., 2016), HVS17 (Hoyos-Villegas et al., 2017), KC15 (Kamfwa et al., 2015), MB14 (Mukeshimana et al., 2014), MK06 (Miklas et al., 2006), MS00b (Miklas et al., 2000b), MC21 (Mir et al., 2021), OP19 (Oladzad et al., 2019), SN11 (Shi et al., 2011), SO21 (Simons et al., 2021), TM01 (Tar’an et al., 2001), TU15 (Trapp et al., 2015), VC15 (Viteri et al., 2015), XK17 (Xie et al., 2017), YP00a (Yu et al., 2000a), YP00b (Yu et al., 2000b), and ZW16 (Zhu et al., 2016)]. QTLs from the meta-analysis (Izquierdo et al., 2023) were labeled as META_name (trait). Additional markers on the map were downloaded from the Legume Information System (LIS; https://www.legumeinfo.org), and background literature was searched and BLASTed against the current common bean genome sequence (*P. vulgaris* v2.1) in Phytozome (https://phytozome-next.jgi.doe.gov). Maps were drawn in MapChart (Voorrips, 2002), and the scale is in Mb.

Thirty-nine significant MTAs were identified between 31 SNPs and 15 yield and yield-related traits on all 11 bean chromosomes using the Bonferroni corrections at *p* = .05. An additional ten MTAs, slightly below the threshold cutoff, were detected for seven traits on five chromosomes. Ten MTAs for eight traits were identified on chromosome Pv01, and single MTAs were found on chromosomes Pv02, Pv10, and Pv11 (Table 3; Figure 4).

**TABLE 3:** SNPs associated with yield and yield-related traits and candidate genes identified in the AYD_AM panel of common beans evaluated over four Ontario location/year environments using the BLINK model in GAPIT 3 software.

Trait^a^	Chromosome	SNP	Position (bp)^b^ in *P. vulgaris* v2.1	*p*-value	MAF	PVE^c^	Gene model	Distance from the SNP (bp)^d^
YD	Pv01	ss715644976	14,734,759	1.6209E-08	0.07826	4.68	*Phvul.001G090100*	−38,631
	Pv01	ss715647097	45,952,069	3.9845E-08	0.43043	8.95	*Phvul.001G200600*	−3,356
	Pv04	ss715646889	1,950,380	1.0570E-06	0.28696	42.49	*Phvul.004G011500*	+592
SW	Pv01	ss715650357	51,402,468	1.6387E-09	0.26522	1.47	*Phvul.001G269300*	0
	Pv04	ss715646796	3,972,770	6.5344E-14	0.26522	14.49	*Phvul.004G034000*	0
	Pv05	ss715645003	10,026,364	1.2265E-06	0.28261	12.19	*Phvul.005G064300*	−39,366
	Pv05	ss715643341	16,005,368	1.6757E-05	0.27826	NA	*Phvul.005G079800*	−14,836
	Pv05	ss715641929	18,743,776	8.1797E-05	0.27391	NA	*Phvul.005G077600*	+18,301
	Pv05	ss715642263	27,001,772	1.6757E-05	0.27826	NA	*Phvul.005G087400*	−36,380
	Pv05	ss715641675	29,096,005	6.2037E-05	0.28696	NA	*Phvul.005G093400*	−26,005
	Pv09	ss715649796	11,080,639	5.2444E-09	0.11304	24.41	*Phvul.009G061100*	0
	Pv09	ss715648556	15,496,055	7.8937E-12	0.11304	8.96	*Phvul.009G098100*	0
	Pv09	ss715645651	32,980,503	1.0660E-06	0.15652	10.26	*Phvul.009G217900*	0
	Pv10	ss715650772	32,892,101	5.9499E-09	0.40000	14.08	*Phvul.010G085900*	0
DF	Pv01	ss715647042	13,312,244	5.2927E-31	0.30000	35.47	*Phvul.001G086100*	0
	Pv02	ss715646980	1,652,590	4.9026E-07	0.45217	3.34	*Phvul.002G015100*	−564
	Pv04	ss715642469	3,638,694	1.0555E-05	0.23478	15.99	*Phvul.004G030500*	−3.691
	Pv07	ss715639576	20,629,979	2.5372E-05	0.09565	NA	*Phvul.007G111400*	−4,531
	Pv08	ss715640684	49,364,726	6.0064E-09	0.11739	11.57	*Phvul.008G174900*	+2,838
DM	Pv01	ss715650911	45,116,577	8.6107E-07	0.14783	41.01	*Phvul.001G192300*	0
	Pv08	ss715640684	49,364,726	2.3294E-05	0.11739	NA	*Phvul.008G174900*	+2,838
	Pv08	ss715647407	62,894,129	3.1064E-06	0.46957	11.50	*Phvul.008G291400*	0
PH	Pv01	ss715645586	4,098,201	1.7827E-06	0.20435	5.12	*Phvul.001G042400*	−12,324
	Pv07	ss715647036	6,095,569	5.8355E-11	0.33043	69.90	*Phvul.007G066580*	−316
HR	Pv03	ss715644902	46,421,101	2.8462E-07	0.30833	28.70	*Phvul.003G231400*	0
	Pv05	ss715645411	38,409,982	5.1312E-07	0.35417	2.22	*Phvul.005G154500*	−1,526
	Pv09	ss715646179	10,324,191	1.9835E-06	0.30417	10.07	*Phvul.009G053600*	−211
RP^e^	Pv08	ss715647407	62,894,129	1.0861E-07	0.46957	47.75	*Phvul.008G291400*	0
YGD	Pv01	ss715644976	14,734,759	9.5666E-08	0.07826	4.20	*Phvul.001G090100*	−38,631
	Pv01	ss715647097	45,952,069	6.4629E-07	0.43043	7.11	*Phvul.001G200600*	−3,356
	Pv03	ss715647091	38,850,086	4.4207E-05	0.37826	NA	*Phvul.003G167800*	−9,097
	Pv04	ss715646889	1,950,380	4.4576E-06	0.28696	41.00	*Phvul.004G011500*	+592
SGR	Pv01	ss715649751	30,278,991	2.5695E-05	0.34783	NA	*Phvul.001G118400*	−43,964
	Pv03	ss715647091	38,850,086	2.6649E-06	0.37826	38.41	*Phvul.003G167800*	−9,097
YDH	Pv05	ss715648062	4,399,405	1.4713E-08	0.40435	46.41	*Phvul.005G042800*	0
SN	Pv06	ss715649801	28,075,639	4.3657E-06	0.13043	52.69	*Phvul.006G178250*	−236
YDHR	Pv01	ss715649523	4,591,153	1.2477E-07	0.12821	14.79	*Phvul.001G038800*	0
	Pv04	ss715646123	45,217,448	4.7019E-07	0.33761	18.56	*Phvul.004G150200*	0
	Pv06	ss715641566	4,470,973	6.2005E-07	0.30342	3.70	*Phvul.006G018200*	+5,650
	Pv09	ss715646179	10,324,191	4.98988–10	0.31197	16.65	*Phvul.009G053600*	−211
CBB_R1	Pv05	ss715647322	7,358,155	1.6948E-06	0.32609	12.96	*Phvul.005G055800*	+9,399
	Pv08	NPP	62,915,925	3.0994E-14	0.15652	30.88	*Phvul.008G291900*	0
CBB_R2	Pv03	ss715650152	27,107,256	3.3949E-05	0.46087	NA	*Phvul.003G100700*	+22,236
	Pv08	NPP	62,915,925	2.0823E-16	0.15652	34.89	*Phvul.008G291900*	0
	Pv09	ss715647179	34,922,930	7.3935E-09	0.30870	13.10	*Phvul.009G232500*	−1,535
	Pv11	ss715648956	47,703,735	1.3097E-06	0.36087	16.89	*Phvul.011G169600*	+3,806
CBB_AUDPC	Pv03	ss715650152	27,107,256	6.0005E-05	0.46087	NA	*Phvul.003G100700*	+22,236
	Pv08	NPP	62,915,925	2.6771E-14	0.15652	37.91	*Phvul.008G291900*	0
	Pv09	ss715647179	34,922,930	2.0639E-06	0.30870	21.92	*Phvul.009G232500*	−1,535

^a^ Measured agronomic traits (ERS and WRS, in 2015 and 2016): YD, yield (kg ha^-1^); SW, seed weight (g); DF, flowering (days); DM, maturity (days); PH, plant height (cm); harvestability (1–5 scale, data collected only in 2016). Derived traits: RP, reproductive period [RP = DM–DF (days)]; YGD, yield gain per day [YGD = YD/DM (kg day^-1^ ha^-1^); SGR, seed growth rate [SGR = YD/RP (kg ha^-1^ day^-1^)]; YDH, yield per unit of height [YDH = YD/PH (kg ha^-1^ cm^-1^)]; SN, seed number [SN = YD/SW (seed number x 106 seeds ha^-1^)]; YDHR, yield per unit of harvestability [YDHR = YD/HR (kg ha^-1^)]. Disease resistance (AAFC, Harrow 2015 and 2016 disease nursery): CBB (common bacterial blight), where CBB_R1 indicates first disease severity scoring (10 days after the inoculation), CBB_R2 denotes second disease severity scoring (10 days after the first scoring), and CBB_AUDPC represents the AUDPC calculated based on two disease scorings using a scale 0–5.

^b^ Physical position (bp) in *P. vulgaris* genome sequence v2.1 (https://phytozome-next.jgi.doe.gov).

^c^ PVE, phenotypic variance explained; NA (not available) if below a Bonferroni threshold of 0.05 (1.114,827^−05^).

^d^ Distance from the SNP, in bp, where minus (−) and plus (+) indicate the gene sequence upstream and downstream, respectively, from the SNPs.

^e^ BLINK was used with five principal components (PCs) in RP analysis. All other traits were analyzed with three PCs to control the population structure.

###### 3.2.3.1.1 Significant associations for measured agronomic traits

**FIGURE 5:**
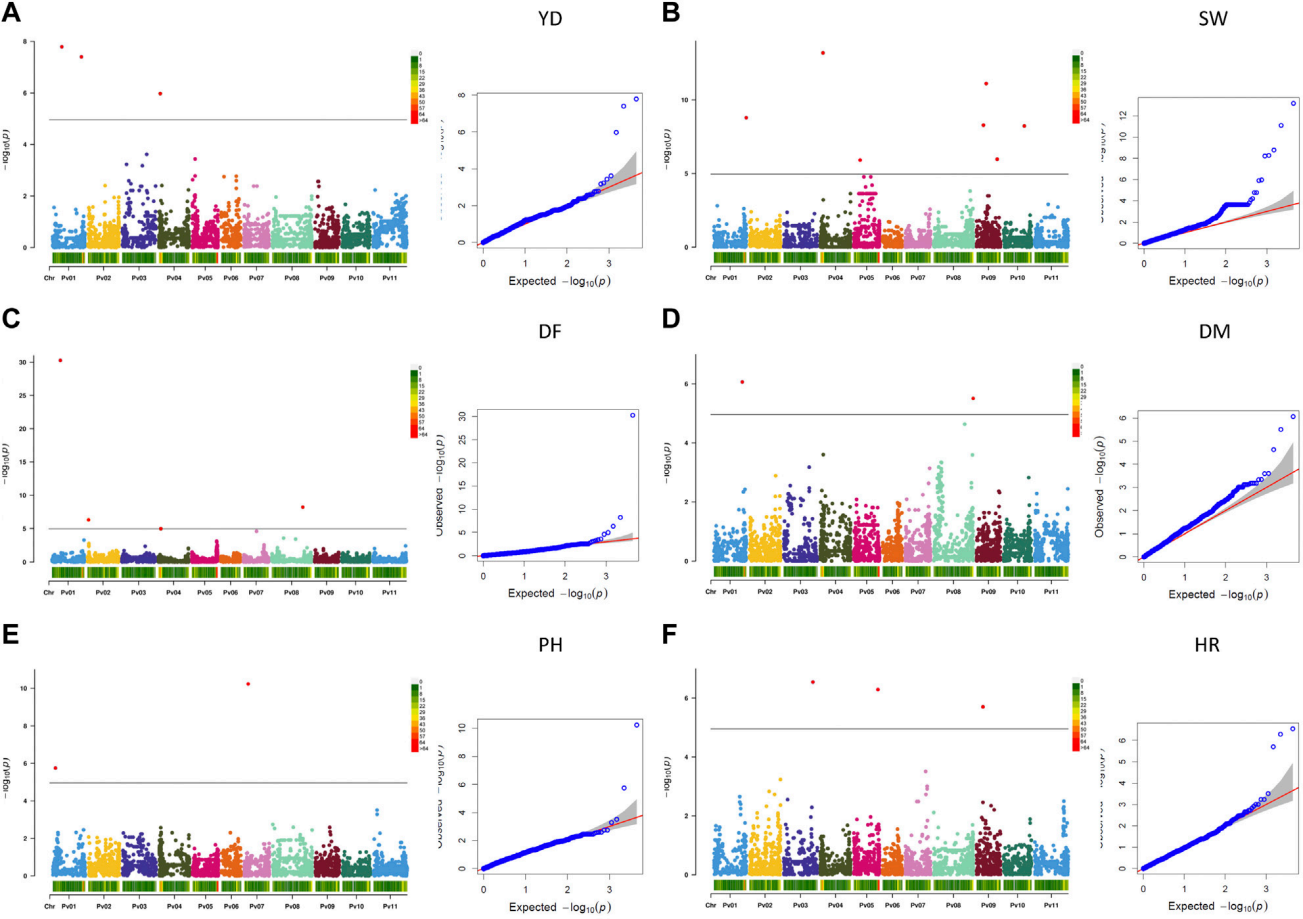
Q-Q plots and Manhattan plots for the six agronomic traits measured in the AYD_AM collection of 121 common bean genotypes in four Ontario location/year environments. The analyses were conducted using BLINK and genotyped with 4,485 SNPs. The shaded areas indicate the 95% confidence intervals. **(A)** Yield (YD), **(B)** seed weight (SW), **(C)** days to flowering (DF), **(D)** days to maturity (DM), **(E)** plant height (PH), and **(F)** harvestability (HR).

Twenty-one MTAs were identified for six measured agronomic traits. Six additional MTAs that were slightly below the Bonferroni threshold were identified for three traits (Table 3; Figures 5A–F, Supplementary Figure S11). The current GWAS analysis did not identify associations between two yield/antiyield gene-based markers (AYD1m and AYD2m) determined previously (Reinprecht et al., 2021) with any of the analyzed traits.

Three MTAs for yield on two bean chromosomes were identified in the current GWAS work. Two MTAs on chromosome Pv01 explained a small portion of phenotypic variation (4.7% and 9%, respectively), while the SNP (ss715646889) on chromosome Pv04 accounted for 42.5% of the variability for yield. Seven MTAs were found for seed weight, three on chromosome Pv09 and one on four other chromosomes (Pv01, Pv04, Pv05, and Pv10), that explained 1.5% (SNP ss715650357 on Pv01) to 24.4% (SNP ss715649796 on Pv09) of the phenotypic variability. Four additional SNPs on Pv05 were also associated with seed weight but were below the Bonferroni threshold.

Four MTAs were detected for flowering on four different chromosomes (Pv01, Pv02, Pv04, and Pv08). They explained 3.3% (ss715646980 on Pv02) to 35.5% (ss715647042 on Pv01) of the phenotypic variability for the trait. An additional SNP on Pv07 (ss715639579) was associated with flowering but was below the threshold cutoff. Two MTAs were identified for maturity on two chromosomes. SNP ss715650911 on Pv01 was associated with 41.0% variability in maturity, while SNP ss715647407 on Pv08 explained 11.5% of the phenotypic variability. An additional SNP on Pv08 was associated with maturity but was slightly below the Bonferroni threshold.

Two MTAs were detected for plant height, and they explained 5.1% (ss715645586 on Pv01) and 69.9% (ss715647036 on Pv07) of the phenotypic variability for the trait. Three MTAs that were identified for harvestability explained 2.2% (on Pv05), 10.1% (on Pv09), and 28.7% (SNP on Pv03) of the phenotypic variability.

###### 3.2.3.1.2 Significant associations for derived traits

**FIGURE 6:**
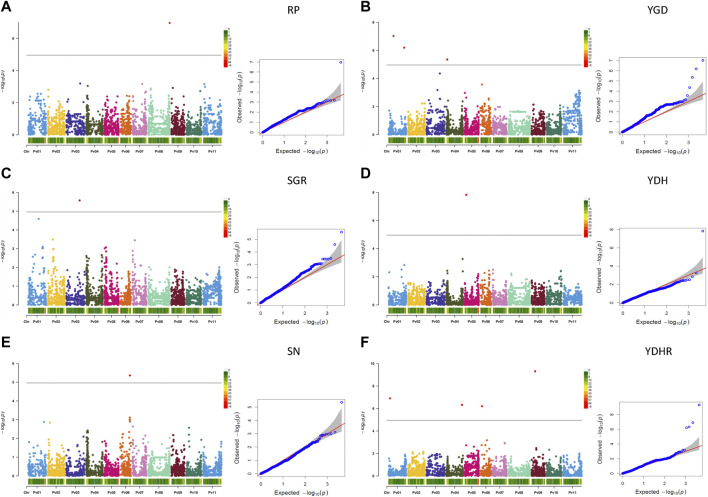
Q-Q plots and Manhattan plots for the six traits derived from the measurements of the yield and yield-related traits evaluated in the AYD_AM collection of 121 common bean genotypes in four Ontario location/year environments. The analyses were conducted using BLINK and genotyped with 4,485 SNPs. The shaded areas indicate the 95% confidence intervals. **(A)** Reproductive period (RP = DM—DF), **(B)** yield gain per day (YGD = YD/DM), **(C)** seed growth rate (SGR = YD/RP), **(D)** yield per unit of height (YDH = YD/PH), **(E)** seed number (SN = YD/SW), **(F)** and yield per unit of harvestability (YDHR = YD/HR).

Based on the Bonferroni threshold cutoff, eleven MTAs were identified for six additional traits that were calculated from the values of the measured traits (Table 3; Figures 6A–F, Supplementary Figure S11). Single MTAs were identified for four derived traits on four different chromosomes (Table 3). An SNP on Pv03 was associated with 38.4% of the variability in SGR, an SNP on Pv05 explained 46.4% of the phenotypic variability in YDH, an SNP on Pv06 was associated with 52.7% of the variability in SN, and an SNP on Pv08 explained 47.8% of the phenotypic variation in RP. An additional SNP associated with the SGR was identified on Pv01. Three MTAs were detected for YGD: two on Pv01 and one on Pv04. They explained 4.2% (SNP on Pv01) to 41.0% (SNP on Pv04) of the phenotypic variability. An additional SNP associated with YGD was identified on chromosome Pv03. Four MTAs were identified for YDHR on four chromosomes (Pv01, Pv04, Pv06, and Pv09). They explained 3.7% (ss715641566 on Pv06) to 16.6% (ss715646179 on Pv09) of the trait variability (Table 3).

###### 3.2.3.1.3 Significant associations for CBB resistance

**FIGURE 7:**
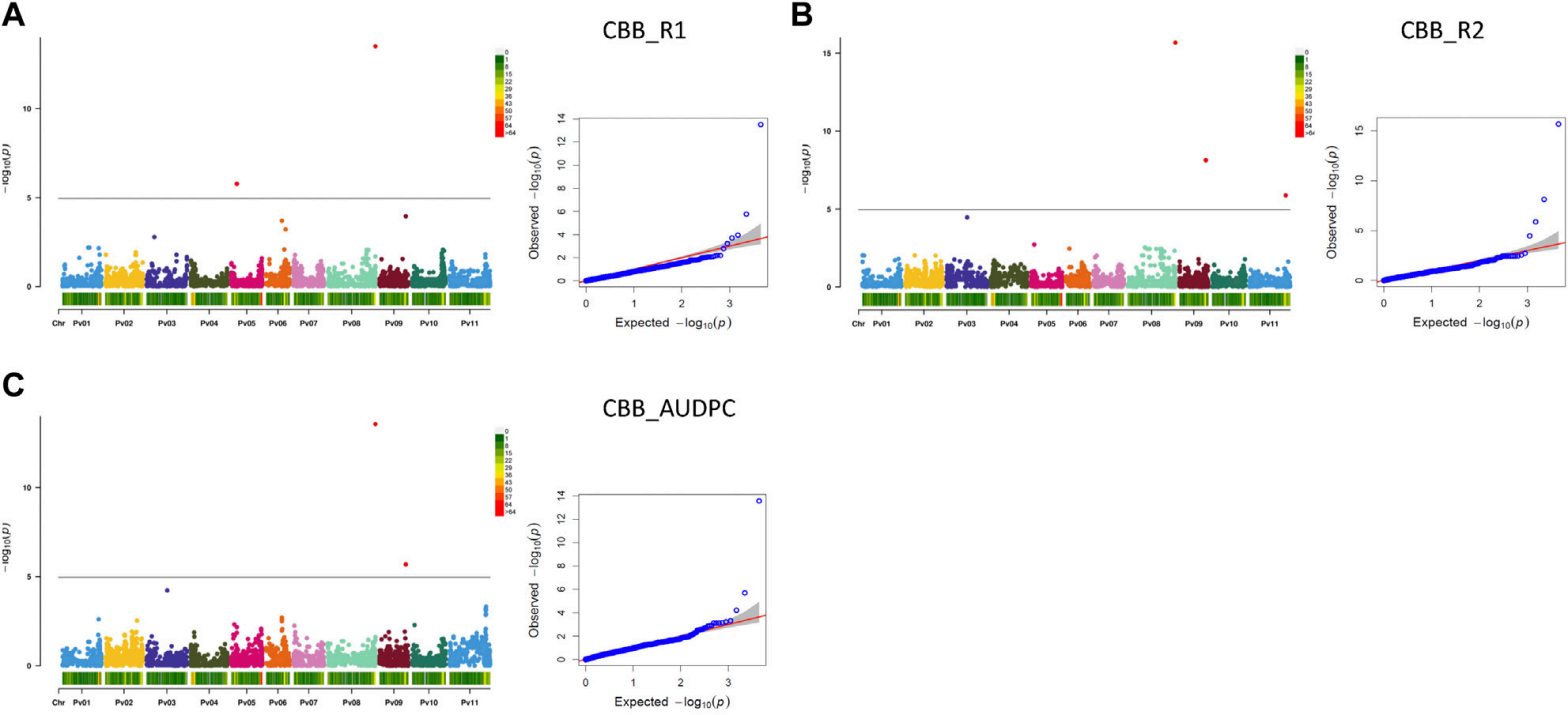
Q-Q plots and Manhattan plots for the resistance to common bacterial blight (CBB) evaluated in the AYD_AM collection of 121 common bean genotypes within the CBB nursery at the Agriculture and Agri-Food Canada Research and Development Centre in Harrow, Ontario, in 2015 and 2016. The analyses were conducted using BLINK and genotyped with 4,485 SNPs. The shaded areas indicate the 95% confidence intervals. **(A)** Common bacterial blight—rating 1 (CBB_R1), **(B)** common bacterial blight—rating 2 (CBB_R2), and **(C)** common bacterial blight—area under disease progress curve (CBB_AUDPC).

Seven MTAs on four chromosomes were detected for CBB resistance that explained significant phenotypic variability for the three measurements of the trait (two CBB readings and AUDPC, treated separately). A gene-based NPP marker (*Phvul.008G291900*) (Morneau, 2019) located close to the end of Pv08 (62.92 Mb) was associated with 30.9% variability in CBB_R1, 34.9% variability in CBB_R2, and 37.9% variability in CBB_AUDPC. SNP ss715647322 on Pv05 was associated with 13.0% of the variability in CBB_R1. SNP ss715647179 on Pv09 (34.92 Mb) explained 13.1% of the phenotypic variability in CBB_R2 and 21.9% of CBB_AUDPC variability. SNP ss715648956 on Pv11 (47.70 Mb) explained 16.9% of the variability in CBB_R2. An additional SNP on chromosome Pv03 (slightly below Bonferroni’s threshold) was associated with CBB_R2 and CBB_AUDPC (Table 3; Figures 7A–C, Supplementary Figure S11).

###### 3.2.3.1.4 SNPs associated with multiple traits

Ten SNPs were related to two or more traits. A gene-based NPP marker was associated with all three assessments of CBB resistance in the AYD_AM panel. Two SNPs were associated with the two CBB measurements (CBB_R2 and CBB_AUDPC). SNP ss715647179 on Pv09 explained 13.1% of the variability in CBB_R2 and 21.9% of the variability in CBB_AUDPC. The association of SNP ss71550152 on Pv03 with these traits was slightly below the Bonferroni threshold. SNP ss715640684 on Pv08 explained 11.6% of the variability in flowering and was also associated with maturity (slightly below the threshold). Five SNPs that were associated with some derived traits were also associated with one of the measured traits. All three SNPs on Pv01 and Pv04 that were associated with yield were also associated with the derived trait YGD and explained similar levels of variability for this trait (Table 3). In addition, SNP ss715647407 on Pv08 explained 11.5% of the variability in maturity and 47.8% variability in RP (derived from maturity and flowering measurements). Similarly, SNP ss715646179 on Pv09 was associated with harvestability (measured) and YDHR (derived) and explained similar phenotypic variabilities for the traits. SNP ss715647091 on Pv03 explained 38.4% of the variability in SGR (YD/RP) and was also associated with the derived trait YGD (YD/DM), but slightly below Bonferroni’s threshold.

##### 3.2.3.2 Candidate gene identification

In total, 627 candidate genes with different functional annotations were identified for 15 analyzed traits in the 200-Kb regions surrounding the SNPs of MTAs. The number of candidates ranged from thirty-seven genes in the area of SNP ss715649801 associated with SN on Pv06 to only two candidate genes in the 200-Kb region centered on SNP ss715645003 linked to seed weight on Pv05 (Supplementary Table S9). In addition to the gene-based NPP marker (*Phvul.008G291900*), 13 SNPs were identified within gene sequences (Table 3). Five additional SNPs significantly associated with five different traits (flowering, yield, seed number, plant height, and harvestability) were located less than 0.6 Kb upstream or downstream from the candidate genes. For nine SNPs, the closest candidate genes were identified more than 10 Kb apart. The distance ranged from 12.3 Kb upstream (plant height on chromosome Pv01) to 44 Kb downstream (SGR on chromosome Pv01) from the SNP. Six of these SNPs were slightly below the Bonferroni significance threshold (Table 3, Supplementary Table S9).

### 3.3 Confirmation of the AYD (yield/antiyield) and NPP (CBB) markers

Single-marker analyses confirmed the associations of AYD and CBB gene-based markers with yield and CBB resistance, respectively.

#### 3.3.1 NPP marker

Five SCAR markers previously associated with CBB resistance (SW13, R4865, SAS13, SU91-CG10, and SAP6) resulted in amplicons of expected sizes. However, none of them were significantly associated with CBB resistance in the current GWAS study. Amplification of the codominant NPP marker in the 121-bean AYD_AM panel was successful, and most of the genotypes produced the expected PCR product sizes (except cultivars Dynasty and Resolute, which did not have successful PCRs). In general, a band of 956 bp was amplified in resistant genotypes, and a 535-bp PCR product was found in CBB susceptible beans. The marker was associated with a significant variation in all three CBB assessments (Table 4), with values (29.7% CBB_R1, 40.3% CBB_R2, and 37.2% CBB_AUDPC) similar to the GWAS findings. The association of this marker with seed weight was small and that with the yield was insignificant. The NPP marker allele for CBB resistance was negatively correlated with the three CBB measurements (Supplementary Table S10).

**TABLE 4:** Trait variation explained by the yield/antiyield markers AYD1m and AYD2m and CBB marker NPP.

Traits		Markers^a^		
		AYD1m	AYD2m	NPP
Measured agronomic traits	Yield (YD, kg ha^-1^)	12.13	6.68	NS
	Seed weight (SW, g)	60.77	74.06	3.47
	Flowering (DF, days)	30.68	36.08	NS
	Maturity (DM, days)	15.41	12.45	3.29
	Plant height (PH, cm)	NS	NS	NS
	Harvestability (HR, scale 1–5)	6.74	14.46	NS
Derived traits^b^	Reproductive period (RP, days)	NS	NS	5.49
	Yield gain per day (YGD, kg ha^-1^ day^-1^)	7.57	2.98	NS
	Seed growth rate (SGR, kg ha^-1^ day^-1^)	14.08	10.24	NS
	Yield per unit of height (YDH, kg ha^-1^ cm^-1^)	16.73	11.91	NS
	Seed number (SN, seed #x 10^6^ seeds ha^-1^)	53.29	53.39	3.19
	Yield per unit of harvestability (YDHR, kg ha^-1^)	NS	NS	NS
Disease resistance^c^	Common bacterial blight (CBB_R1)	3.65	NS	29.67
	Common bacterial blight (CBB_R2)	6.02	5.20	40.31
	Common bacterial blight (CBB_AUDPC)	4.54	2.79	37.24

^a^ Markers: AYD1m (AYD, gene 1, *Phvul.009G190100*) and AYD2m (AYD, gene 2, *Phvul.009G202100*), yield/antiyield markers (Reinprecht et al., 2021); NPP (gene *Phvul.08G291900*), Niemann–Pick polymorphism (NPP) CBB marker (Morneau, 2019).

^b^ Derived traits: RP, reproductive period [RP = DM–DF (days)]; YGD, yield gain per day [YGD = YD/DM (kg day^-1^ ha^-1^); SGR, seed growth rate [SGR = YD/RP (kg ha^-1^ day^-1^)]; YDH, yield per unit of height [YDH = YD/PH (kg ha^-1^ cm^-1^)]; SN, seed number [SN = YD/SW (seed number x 106 seeds ha^-1^)]; YDHR, yield per unit of harvestability [YDHR = YD/HR (kg ha^-1^)].

^c^ Disease resistance (AAFC, Harrow 2015 and 2016 disease nursery): CBB (common bacterial blight), where CBB_R1 indicates first disease severity scoring (10 days after the inoculation), CBB_R2 denotes second disease severity scoring (10 days after the first scoring), and CBB_AUDPC represents the AUDPC calculated based on two disease scorings using a scale of 0–5.

**FIGURE 8:**
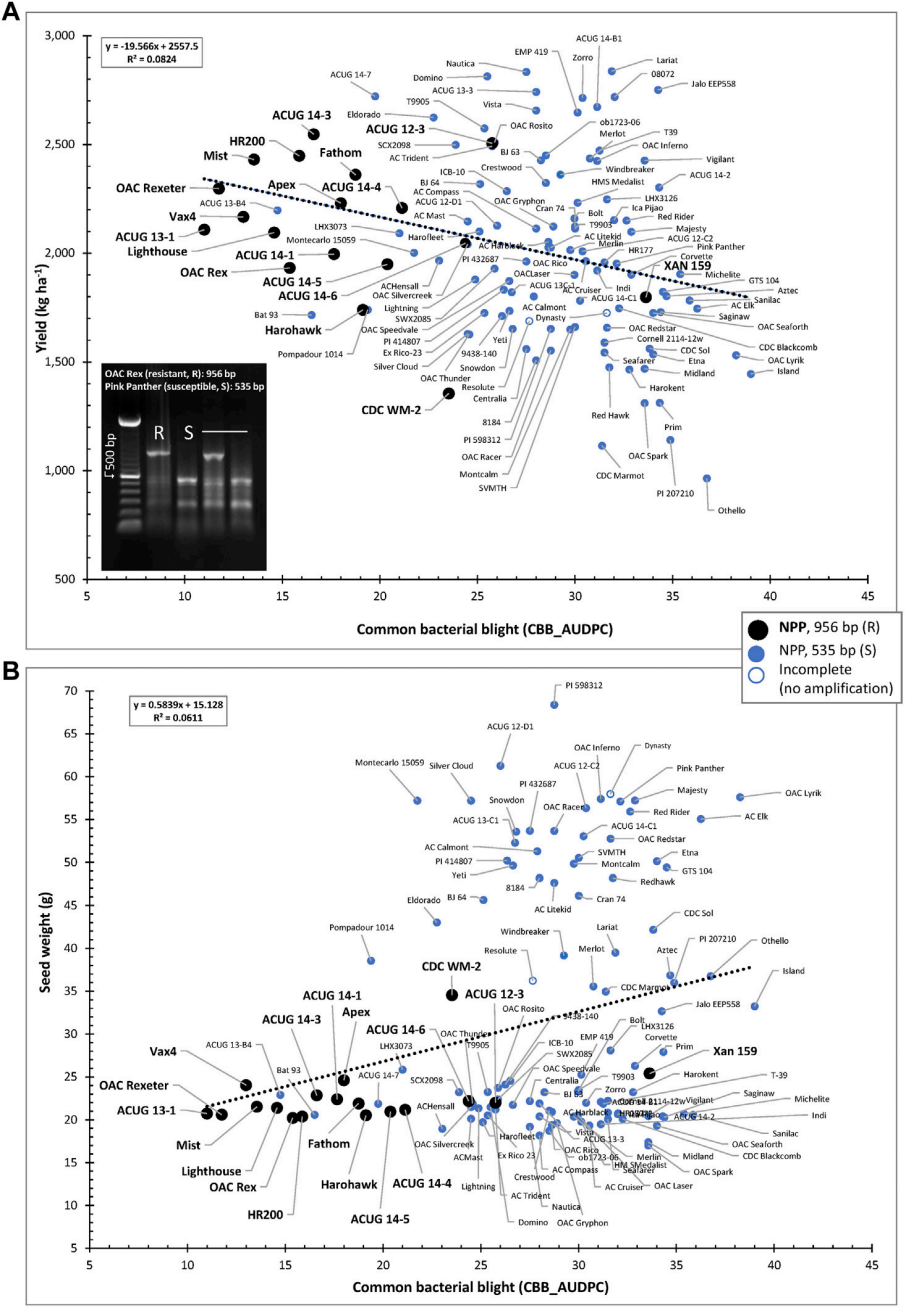
Relationships between CBB susceptibility and yield **(A)** or seed weight **(B)** in the AYD_AM collection of common beans. The allelic state of the NPP marker (956-bp fragment in resistant lines and 535 bp in the susceptible lines) is indicated.

The usefulness of the NPP marker is further extended through the negative association between yield and CBB resistance (CBB_AUDPC) as well as the positive correlation between CBB and seed weight. In general, small-seeded genotypes resistant to CBB and containing the NPP resistant marker allele were grouped in the top left portion of the CBB-YD scatter plot (indicated in black in Figure 8A) and in the bottom left portion of the CBB-SW scatter plot (Figure 8B).

#### 3.3.2 Yield/antiyield (AYD) markers

**FIGURE 9:**
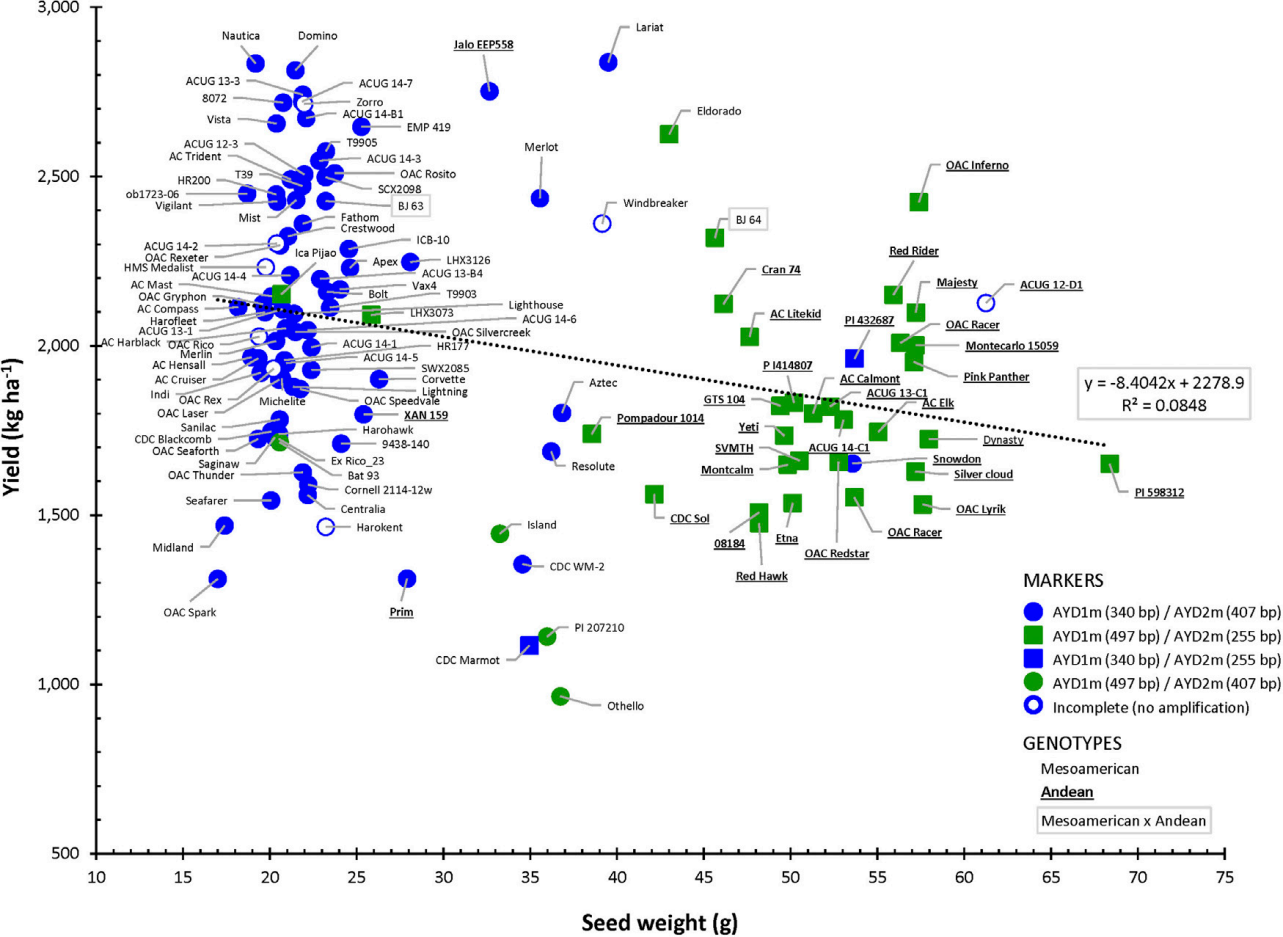
Relationship between seed weight and yield in the AYD_AM collection of common beans and the allelic state of yield/antiyield (AYD1m and AYD2m) markers.

Amplification of the two yield/antiyield TSP markers in the AYD_AM panel of 121 beans was successful, and most of the genotypes produced the expected PCR product sizes. Genomic DNA from three genotypes (ACUG 14-2, Harokent, and Zorro) did not amplify with both markers. An additional five genotypes (ACUG 12-D1, HMS Medalist, OAC Rex, OAC Rico, and Windbreaker) did not produce any PCR product with the AYD2m marker (Figure 9).

Based on the marker allele, the AYD_AM panel was split into two groups. From the larger group of 76 genotypes (62.8%), consisting mainly of small-seeded Mesoamerican beans, a 340-bp AYD1m band was amplified (corresponding to a T–G mutation in the AYD1 gene *Phvul.009G190100*) and a 407-bp AYD2m band was amplified (corresponding to a T–A mutation in the AYD2 gene *Phvul.009G202100*) (AYD1m_340/AYD2m_407, left side in Figure 9). From the second group, consisting of 31 (25.6%) mostly large-seeded Andean beans, a 497-bp AYD1m band and a 255-bp AYD2m band were amplified, which corresponded to the wild-type (G19833, *P. vulgaris* v2.1) alleles (AYD1m_497/AYD2m_255, right side in Figure 9). Six genotypes produced a combination of the two types of alleles amplified by AYD1m and AYD2m markers (categories AYD1m_340/AYD2m_255 and AYD1m_497/AYD2m_407, respectively). Eight genotypes could not be categorized due to the incomplete amplification of one or both markers.

There was a significant negative correlation between the two markers [r (113) = −0.8766, *p*=<0.0001], and they were in opposite relationships with the analyzed traits. The marker AYD1m was positively correlated with yield, flowering, maturity, harvestability, and four derived traits (YGD, SGR, YDH, and SN), while the AYD2m marker had a positive relationship with seed weight (Supplementary Table S10). The strongest association of these markers was with seed weight. Marker AYD1m explained 60.8% variability for the trait, and AYD2m accounted for the 74.1% variability in seed weight (Table 4).

## 4 Discussion

Superior yield is the trait that distinguishes a genotype in a plant breeding program from all the others when selection decisions are made. New cultivars often incorporate attributes such as disease resistance or enhanced crop quality into a package, but for widely used cultivars, such traits are incorporated into highly productive genotypes because the use of high-yielding cultivars by producers enables them to meet challenges related to rising input, labor, and land costs. In spite of its desirability, selection for yield is difficult because it is controlled by many loci, affected by environmental conditions, and its evaluation is labor- and infrastructure-intensive.

### 4.1 Assembly of the AYD_AM panel of beans

The bean germplasm was included in the AYD_AM panel because it contributed diversity or was a line that was used or developed by the University of Guelph bean breeding program. The genotypes belonged to Mesoamerican and Andean gene pools, different market classes, and were released between 1937 and 2019. The panel consisted of 121 genotypes, of which the majority (85 genotypes or 70.2%) were Mesoamerican. The mix of genotypes and market classes reflected the structure of the University of Guelph bean breeding program, which is developing new cultivars for production in Southern Ontario, consisting of 58% navy, 16% black, 17% kidney, and 9% cranberry beans.

### 4.2 Heritability estimates, variability in analyzed traits, and relationships among traits

In addition to the yield and a number of yield-related traits, which are routinely collected in the bean breeding program, the current study evaluated beans for a few derived (index) traits because they (especially the yield/days to maturity) are commonly used as selection criteria in development of new cultivars.

High positive associations among the environments for most of the analyzed traits and moderate-to-high estimates of broad-sense heritability for the traits [47.2% (CBB_R1) to 98.3% (SW)] supported averaging the analyses over four location/year environments. Furthermore, in the combined analysis, significant variation among genotypes was identified for all analyzed traits.

Spearman’s rank correlation analysis revealed the existence of complex relationships among the traits. Particularly interesting were the inverse relationships of yield with seed weight and CBB scores. Several studies have identified a negative relationship between yield and seed weight (White and Gonzalez, 1990; Reinprecht et al., 2020). CBB can significantly reduce yield in susceptible cultivars (Tar’an et al., 2001; Gillard et al., 2009; Durham et al., 2013), and a negative association between yield and CBB has been reported previously (Boersma et al., 2015). The positive associations between CBB scores and seed weight may be related to pleiotropy or linkage because generally, large-seeded Andean genotypes do not have significant levels of CBB resistance (Mutlu et al., 2008; Viteri and Singh, 2014). The negative relationship between CBB scores (susceptibility) and days to maturity is consistent with previous work showing that the late maturing genotypes were more resistant than the early maturing beans (Tar’an et al., 2001; Diaz Castro, 2015). This may be related to linkage drag of late maturity from interspecific sources of CBB resistance incorporated mostly into Mesoamerican cultivars (Ambachew et al., 2021).

These correlations may indicate linkage, pleiotropy, or similar environmental effects on trait expression (Aastveit and Aastveit, 1993). A better understanding of the nature of the correlations among traits is needed to facilitate the combination of high yield with CBB resistance and other desirable phenological and agronomical traits. This information will be useful not only for the direct selection of complex traits, such as yield, but also to avoid unintentional selection for traits that could slow breeding progress (Cobb et al., 2019).

### 4.3 Genotyping and GWAS

The importance of SNP filtering steps in GWASs, which is, in general, related to reducing false associations, has been reviewed previously (Marees et al., 2018; Pavan et al., 2020). As expected, the 4,485 SNPs that remained were not uniformly distributed across the 11 bean chromosomes and along individual chromosomes, with Pv06 and Pv03 having the lowest proportion of SNPs (6.2% and 6.5%, respectively) and Pv05 and Pv02 having the highest proportion (11.6% and 11.3%, respectively) and higher SNP densities toward chromosome ends, while generally sparser in the repetitive DNA-rich pericentromeric regions. The genomic patterns of diversity observed in the current study agree with those observed for the bean reference genome (Schmutz et al., 2014).

#### 4.3.1 Genetic diversity, population structure, and LD

The nucleotide diversity in the AYD_AM panel of 121 genotypes used in the current work, as measured by π (0.387 per bp) for the 4,485 SNPs, was higher than the values (ranging from 0.251 to 0.309) measured for a panel of 180 bean genotypes, representative of the genetic diversity deposited at the Agronomic Institute (IAC) in Campinas, Brazil, which included commercial cultivars from different breeding institutions [including the International Center for Tropical Agriculture (CIAT) and the Brazilian Agricultural Research Corporation (Embrapa)], landraces, and parents of several mapping populations (Diniz et al., 2019). For a collection of 86 wild geo-referenced *P. vulgaris* genotypes representing Mesoamerica, Guatemala, Colombia, Ecuador/Northern Peru, and Andean groupings, the average nucleotide diversity as measured by π was 0.3 per million base pairs, average Watterson’s θ was 0.20 per million base pairs, and average Tajima’s D was 0.68 per million base pairs (Cortés and Blair, 2018). In the current study, average Watterson’s theta (θ) was 0.187 per base pair, and average Tajima’s D was 3.606 per base pair. The positive Tajima’s D value indicates that genotypes of the AYD_AM panel are under balancing selection or population contraction (a few alleles at high frequency). Balancing selection results in a higher level of sequence diversity and an excess of intermediate-frequency variants (Delph and Kelly, 2014). A significant number of SNPs having minor alleles with frequency less than 5% were removed. Furthermore, filtering for less heterozygous markers could have left fewer than expected rare alleles in the collection. Since frequency-based filters were applied in this analysis, Tajima’s D used to detect departures from neutrality could be overestimated. However, diversity calculations with raw SNP data (5,300 SNPs) provided very similar diversity trends (π = 0.369, θ = 0.185, and Tajima’s D = 3.343). Small populations tend to have less allelic diversity, and a loss of genetic diversity is faster in small populations than in the large populations (Leimu et al., 2006). That might be the case with the AYD_AM panel.

When the year of cultivar release was considered, 71 new cultivars released after 2000 had higher π (0.388) and Tajima’s D (3.090), while the 50 cultivars (released in or before 2000) had higher θ (0.223). This indicated that diversity was not lost with the new cultivar releases. Mesoamerican genotypes (87 genotypes) were more diverse (π = 0.246, θ = 0.172, Tajima’s D = 1.486) than the 34 Andean beans, supporting the different domestication pathways in the two gene pools (Schmutz et al., 2014).

As expected from other reports (Kwak and Gepts, 2009; Blair et al., 2012b; Delfini et al., 2021), the STRUCTURE analysis for K = 2 and the phylogenetic tree analysis split the AYD_AM panel into two subpopulations according to Andean and Mesoamerican gene pools. When the membership coefficient of ≥0.60 was used, the Andean beans accounted for 28.9% of the genotypes (34) in the AYD_AM panel. In addition, some level of admixture was identified in 26.5% genotypes, which may be related to breeding histories and shared parentage.

Interestingly, two genotypes considered to be Andean (Jalo EEP558 and XAN 159) were grouped within the Mesoamerican gene pool based on 4,485 filtered SNPs used in the current study (both 0.773 M and 0.227 A). Jalo EEP558 is a Brazilian landrace with an unknown pedigree, which was extensively used in genetic studies. The line was the Andean parent in the Bat93 x Jalo EEP558 cross used in the development of the bean reference genetic map (Freyre et al., 1998). XAN 159 is a breeding line derived from an interspecific cross with *P. acutifolius* accession G 40020 (PI 319443) with medium-sized multicolored seeds and is resistant to CBB (McElroy, 1985). Alternatively, a small-seeded navy bean cultivar OAC Silvercreek (0.996 A and 0.004 M), which has a cranberry cultivar (Cran 74) in its background, was grouped with the Andean beans. Some gene pool membership incongruency was also reported for a STRUCTURE analysis of wild and domesticated Andean and Mesoamerican accessions characterized with microsatellite markers (Kwak and Gepts, 2009). Gene pool miss-assignment was also reported in an analysis of geographical distribution of 246 wild *P. vulgaris* accessions with ∼20,000 GBS-derived SNPs (Ariani et al., 2018).

However, when the three populations within the AYD_AM panel were considered (K = 3), the Andean genotypes remained clustered within a unique subpopulation, whereas the Mesoamerican beans formed several subpopulations with significant admixture, which were indistinguishable based on a market class (pinto, small red, navy, and black). Similar groupings were reported with Brazilian beans in a GWAS analysis for bio-climatic variables (Elias et al., 2021). Inconsistent clustering patterns of genotypes by market class were also observed in peanuts (Otyama et al., 2019). The authors of the study concluded that the market type was not well-predicted by the genotype and speculated that the traits associated with market type may be determined by small genomic regions so that the phylogenetic signal from those regions is masked by other regions. In the current work, the relatively small population size (121 genotypes used in the structure analysis), SNP filtering (for minor alleles and heterozygosity), and crossing among market types may also be associated with the difficulty to differentiate market classes.

The long linkage blocks and high LD values between SNPs observed for the AYD_AM panel in the current study were consistent with those observed in previous studies that found that LD decay in *Phaseolus* is generally low (Valdisser et al., 2017; Campa et al., 2018). Similar to other studies (Blair et al., 2018; Delfini et al., 2021), the current study found that the rate of decay varied among chromosomes. LD decay values determine the number of SNPs required for QTL discovery and mapping resolution. Factors that affect LD include recombination rates along chromosome lengths, population structure, the extent of inbreeding (LD decay is delayed in selfing populations), mutation, and gene flow (Flint-Garcia et al., 2003). Because the population structure can affect estimates of LD (Burle et al., 2010; Vos et al., 2017), it would be beneficial to conduct additional studies of the University of Guelph bean breeding germplasm with larger numbers of genotypes in separate gene pools.

#### 4.3.2 Significant associations, candidate genes, and QTL/marker validation

GWAS was performed using the BLUEs of AYD_AM genotype performances averaged over four Ontario location/year environments to minimize errors with multi-environment data and analysis of incomplete block experimental designs such as lattice designs (Henderson, 1975). Based on the Q-Q plots, BLINK was the most suitable method to identify significant MTAs with the 4,485 SNPs in the AYD_AM collection of 121 bean genotypes. BLINK uses a multi-locus model for testing markers across the genome and runs two fixed-effect models iteratively, eliminating the requirement that quantitative trait nucleotides (QTNs) underlying a trait are distributed equally across the genome and improve statistical power over other algorithms (Huang et al., 2019).

Over 600 candidate genes were identified for 15 analyzed traits in the 200-Kb region centered on significant SNPs. The number of genes in the 200-kb regions ranged from two to thirty-seven, but only a single gene closest to the significant SNPs was considered candidate genes in the current work. Among them, 14 SNPs were found within the gene model sequences and five additional SNPs were located less than 0.6 Kb from the candidate genes. These genes have various functional annotations and are involved in different molecular processes. The roles they may play in the expression of the analyzed traits need to be addressed in future studies. With a few exemptions, these MTAs explained 10%–70% of the phenotypic variation in the analyzed traits. The current discussion focuses only on the most significant MTAs. Molecular markers developed based on the sequences of these genes [similar to the NPP marker (gene *Phvul.008G291900*), Morneau (2019)] may find an immediate use in direct and/or indirect breeding for high-yielding bean cultivars.

Although genetic and physical maps have been integrated in bean (Córdoba et al., 2010), the incorporation of previously mapped QTL was not straightforward. Even when the order of the physical map (bp or Mb) and the genetic map (cM, a unit of recombination frequency) was identical, the value of Mb vs. cM between markers was not proportional. Some QTL filled up almost the entire chromosome. For example, flowering QTL DF1.1 (Blair et al., 2006) mapped to the 6.79–48.35-Mb region on Pv01 of the current physical map. These regions can contain thousands of genes. In this study, only a fraction of candidate genes were identified. Since QTL can span large regions and contain hundreds of genes, the other genes within the investigated 200-Kb region centered on the SNPs need to be evaluated.

##### 4.3.2.1 Yield, seed weight, and yield gain per day (YGD) MTAs

Yield and seed weight QTL were identified on all 11 chromosomes in previous QTL/GWASs (Blair et al., 2006; Checa and Blair, 2012; Mukeshimana et al., 2014; Trapp et al., 2015; Diaz et al., 2020). Some of them were co-localized, and their most stable locations were recently confirmed in a meta-analysis for 394 QTL reported in 21 independent studies (Izquierdo et al., 2023). In the current study, MTAs for both traits were identified on different locations on Pv01 and Pv04.

Two SNPs associated with yield and YGD were located close to a previously identified yield QTL on Pv01 (Trapp et al., 2015; Diaz et al., 2020; Izquierdo et al., 2023). The SNP (ss715646889) on Pv04 was associated with over 40% of the variability in yield, and YGD is located close to the previously identified yield QTL on this chromosome (Diaz et al., 2018). The closest candidate gene on PV04 is *Phvul.004G011500*, located 592 bp downstream of ss715646889. It encodes a NADH-cytochrome b5 reductase, which is required for correct pollen function and seed maturation in *Arabidopsis* (Wayne et al., 2013). In addition, four *PvSWEET* genes were identified in the 200-Kb region centered on the SNP. They belong to the 24-member sugar will eventually be exported transporter (SWEET) gene family, which is involved in plant development and response to abiotic stress (Du et al., 2022). In soybean, gene *GmSWEET39* (bean homolog *Phvul.006G210800*) encodes a protein with probable functions as a seed coat-specific sugar transporter in seed development and has a pleiotropic effect on seed protein and oil contents (Zhang et al., 2020). Since this MTA is associated with such a large portion of the phenotypic variability of the trait, future work should focus on determining the involvement of these genes in yield and YGD in bean.

The SNP associated with seed weight on Pv01 was located 3.76 Mb from a previously identified QTL (Trapp et al., 2015) and MQTL (Izquierdo et al., 2023). It occurs within a gene model *Phvul.001G269300*, which encodes a mediator of RNA polymerase II transcription subunit 13 (MED13) and functions in a mediator complex controlling developmental transitions in plants (Zhang and Guo, 2020). The SNP (ss715646796) associated with the seed weight on Pv04 is located 4.53 Mb from the previously detected QTL (Diaz et al., 2020) and occurs within a gene model *Phvul.004G034000*, which encodes ATP-dependent chaperone ClpB, involved in response to heat and protein processing (Parcerisa et al., 2020).

Three seed weight MTAs on Pv09 were found in similar locations as those of previously mapped QTL/MTAs on this chromosome (Blair and Izquierdo, 2012; Hoyos-Villegas et al., 2016; Hoyos-Villegas et al., 2017; Diaz et al., 2018; Izquierdo et al., 2023) and are within candidate genes. SNP ss715649796 (positioned at 11,080,639 bp) is within the *Phvul.009G061100* gene model, which encodes an AP-4 complex subunit mu-1 (AP4M1)], involved in sorting vacuolar proteins in plants (Fuji et al., 2016). SNP ss715648556, associated with the seed weight on Pv09 (positioned at 15,496,055 bp), is in the *Phvul.009g098100* gene model, which encodes an embryo-defective 2410 protein (DUF490). In *Arabidopsis*, gene *At2g25660* (encoding emb2410) is required for embryo development (Tzafrir et al., 2004). SNP ss715645651 in the *Phvul.009G217900* gene model, which encodes vacuolar sorting-associated protein 54 (VPS54), plays a role in the vacuolar trafficking pathway (Delgadillo et al., 2020).

The MTA identified on Pv10 is 11.32 Mb from the previously identified QTL (sw10.1; Blair et al., 2006) and represents a new region associated with this trait. The SNP (ss715650772) is within the gene model *Phvul.010G085900*, which encodes an acyl-CoA synthetase [EC:6.2.1.-, (AASDH)], involved in lipid biosynthesis. The acyl-CoA synthetase encoded by the *LACS2* gene is essential for normal cuticle development in *Arabidopsis* (Schnurr et al., 2004). The function of these genes in seed weight, as an important component of yield in beans, should be addressed in future studies.

##### 4.3.2.2 Flowering, maturity, and reproductive period (RP)

Previous studies identified QTL/MTAs for phenological traits on all 11 bean chromosomes. Co-localization of phenological QTLs was reported previously and confirmed in a meta-analysis (Izquierdo et al., 2023). The flowering MTA on Pv01 (13,312,244 bp), in the current study, mapped to a similar location as previously identified flowering regions on this chromosome [0.28 Mb from DF1.1 (Hoyos-Villegas et al., 2016) and 1.36 Mb from DF1.2 (Diaz et al., 2020)]. The SNP ss715647042 is located within the *Phvul.001G086100* gene model, which encodes nuclear cap-binding protein subunit 1 (NCBP1, CBP80), involved in a number of processes including abiotic stress responses (Daszkowska-Golec, 2018). Bezerra et al. (2004) described the early flowering phenotype of the Atcbp80/abh1 mutant, and the mutation in CBP80 was linked to the suppression of the late-flowering phenotype. The maturity MTA on Pv01 (45,116,577 bp) mapped 0.67 Mb (Almeida et al., 2020) and 3.19 Mb (Kamfwa et al., 2015) from maturity regions identified on this chromosome previously and confirmed in a recent meta-analysis (Izquierdo et al., 2023). The SNP ss715650911 is located within the candidate gene *Phvul.001G192300*, which encodes the UDP-N-acetylglucosamine-peptide N-acetylglucosaminyltransferase SPINDLY-related protein, with the highest expression in green mature pods (Phytozome). Initially identified as a negative regulator in the gibberellin signal transduction pathway (Jacobsen and Olszewski, 1993), the O-GlcNAc transferase (OGT) SPINDLY (SPY) has an overlapping function with the SECRET AGENT (SEC) in leaf production and reproductive development in *Arabidopsis* (Hartweck et al., 2006).

The SNP ss715640684 on Pv08 was associated with both flowering and maturity (slightly below the significance threshold) and mapped 11.81 Mb from DF8 (Kamfwa et al., 2015) and 0.71 Mb from the maturity QTL detected by Galeano et al. (2012). The flowering MTA can be considered a new location on Pv08 for this trait. The closest candidate gene *Phvul.008G174900* is located 2,838 bp downstream from the SNP. It encodes a protein kinase domain (Pkinase)//Wall-associated receptor kinase galacturonan-binding (GUB_WAK_bind). In *Arabidopsis*, AtWAK1 was involved in plant stress responses (He et al., 1998). The second SNP on chromosome Pv08 associated with maturity was also related to the RP and was located within the MQTL-YC8.5 (QTN.DM_DF_SW) region (Izquierdo et al., 2023). It is located within the candidate gene *Phvul.008G291400*, which encodes chloroplastic MATE efflux family protein 3 (multi-antimicrobial extrusion protein) involved in transmembrane transport. MATEs are involved in regulating many agronomic traits including seed color, dormancy, and response to stress (Ku et al., 2022).

The flowering MTA on Pv04 is located 0.5–2.22 Mb from the previously identified flowering QTL on this chromosome (González et al., 2021). It is located 3,691 bp from the candidate gene *Phvul.004G030500*, which encodes an unannotated protein. The SNP ss715646980 associated with flowering on Pv02 [1.25–1.51 Mb from MQTL-YC2.2 (QTN.DF), Izquierdo et al. (2023)] is located 564 bp upstream of the closest candidate gene *Phvul.002G015100*, which encodes an Myb transcription factor [IPR009057 (homeodomain-like)], which is highly expressed in green mature pods, stems, and flower buds (Phytozome). Similarly, an SNP on Pv07 (slightly below Bonferroni’s threshold) that was associated with flowering was mapped within the two overlapping MQTL regions detected by Izquierdo et al. (2023). The closest candidate gene *Phvul.007g111400* is located 4,531 bp upstream of the SNP and encodes the protein YLS7-related [*Glycine max*]/IPR025846 (PMR5 N-terminal domain)/IPR026057 (PC-Esterase)/trichome birefringence-like family. In *Brassica napus*, gene *BnaA03g47330D*, homolog to *Arabidopsis AT4G25360*, was identified as a candidate gene for lodging (Li et al., 2018). It encodes a member of the TBL gene family (*TBL18*) involved in the synthesis and deposition of secondary wall cellulose.

##### 4.3.2.3 Plant height and harvestability

Two MTAs that were detected for plant height can be considered new locations for this trait since they were located away from previously identified QTL associated with height (Blair et al., 2006). For example, the MTA found on Pv01 was found 8.38–42.49 Mb from the QTL identified previously. The closest candidate gene *Phvul.*001G042400 is located 12,324 bp upstream of the SNP and encodes an ER membrane protein complex subunit 4 (DUF1077, TMEM85), involved in biogenesis of membrane proteins (Shurtleff et al., 2018). The SNP (ss715647036) on Pv07 was located 5.97 Mb from the previously identified QTL on this chromosome (Blair et al., 2006). The candidate gene *Phvul.007G066580* is located only 316 bp upstream from the SNP and encodes one of the two MATE efflux family proteins and is highly expressed in roots and nodules (Phytozome). MATE transporters were associated with the nodulation and nitrogen fixation in a GWAS with the Middle American panel of beans (Oladzad et al., 2020). Plants contain a large family of MATEs with distinct roles. Islam et al. (2022) identified 59 putative MATE transporters in bean. Among them, gene *PvMATE8* was associated with seed coat darkening in pinto beans.

Three MTAs that were identified for harvestability explained 2.2% (Pv05) to 28.7% (Pv03) of the phenotypic variability and are the first genome locations reported for this trait. The MTA on Pv03 is within a candidate gene *Phvul.003G231400*, which encodes for one of 43 proteins containing a protein kinase domain (Pkinase)//leucine-rich repeat (LRR_1)//leucine-rich repeat N-terminal domain (LRRNT_2)//leucine-rich repeat (LRR_8) (Phytozome). Aono et al. (2023) identified 1,203 putative protein kinases in the bean genome, which were classified into 20 groups and 119 subfamilies, many involved in stress responses. The SNP ss715645411 associated with harvestability on Pv05 was located 1,526 bp upstream of the candidate gene *Phvul.005G154400*, which encodes one of 55 GRAS domain family/scarecrow-like transcription factors that have diverse functions in stress responses, shoot and root development, and gibberellic acid signaling (Cenci and Rouard, 2017). The third SNP associated with harvestability (ss715646179) was also linked with YDHR (trait derived from the yield and harvestability measurements). It is located 211 bp upstream from the candidate gene *Phvul.009G053600*. This gene encodes a nipped-B-like protein, a cohesin loading factor, involved in chromatin binding and regulation of gene expression (Newkirk et al., 2017).

##### 4.3.2.4 Derived traits

In total, 13 MTAs (two below the significance threshold) were identified for six additional traits that were calculated from the values of the measured traits. Most of these associations were not reported previously, and a number of the candidate genes encode proteins of unknown function. The SNP ss715647091, on Pv03, was associated with the SGR and YGD (below Bonferroni) and was located within a yield QTL SY3.3 (a measured trait) identified previously (Hoyos-Villegas et al., 2016). The MTA associated with YGD was located 34.64 Mb from the previously reported QTL associated with this trait (Mukeshimana et al., 2014). The closest candidate gene (*Phvul.003g167800*), located 9,097 bp upstream from the SNP, encodes the Rab-GTPase-TBC domain-containing protein (DUF3548), which is involved in growth and development, plant–microbe interactions, and responses to biotic and abiotic stresses (Tripathy et al., 2021). Two MTAs identified for YDHR were within candidate genes. The SNP ss715648523 on Pv01 is within the gene model *Phvul.001G038800*, which encodes a cytokinin dehydrogenase 2-related protein, which has been characterized as a target for yield improvement in wheat (Chen et al., 2020). Similarly, SNP ss715646123 on Pv04 was within the gene model *Phvul.004G150200*, which encodes aldose-1-epimerase, a key enzyme in carbohydrate metabolism and is involved in biotic and abiotic stress control (Sheshukova et al., 2017). The MTA on Pv06 was located 5,650 bp downstream from the candidate gene *Phvul.006G018200*, which encodes an F-box domain (F-box)//Kelch motif (Kelch_1) that controls organ size in *Arabidopsis* (Wang et al., 2016). Future work should focus on investigating the potential roles these genes play in conditioning yield and yield-related traits in beans. All three YDHR MTAs are within or close to QTL/MQTL previously identified for yield (measured trait). The MTA on Pv01 is 0.14 Mb from the yield QTL YD1.1_DR18 reported by Diaz et al. (2018), while the MTA on Pv04 is within the yield MQTL-YC4.5 (YD), and the MTA on Pv06 is 4.07 Mb from the yield MQTL-YC6.1 (QTN.DM_SW_YD_DF) identified by meta-analysis (Izquierdo et al., 2023).

##### 4.3.2.5 CBB resistance

Previous studies reported over 25 QTL/MTAs for CBB resistance on all 11 bean chromosomes. In the current work, MTAs for three measurements of CBB symptom development identified on Pv03, Pv05, Pv08, and Pv11 were in genome regions associated with the trait in earlier studies.

Our current results confirmed the usefulness of the NPP (*Phvul.008G291900*) gene-based marker (Morneau, 2019) for screening bean genotypes for CBB resistance. It mapped to the same region on Pv08 as the major CBB QTL SU91 identified previously in a number of studies (Miklas et al., 2000a; Shi et al., 2011; Perry et al., 2013; Trapp et al., 2015; Xie et al., 2017; Simons et al., 2021). The Niemann–Pick genes in *Arabidopsis* encode proteins with homology to the mammalian Niemann–Pick C1, which functions in cholesterol transport. The Niemann–Pick C1 genes are essential for plant development and reproduction (Feldman et al., 2015), but the role played by the Niemann–Pick-like protein in disease resistance is unknown. It may be related to its proposed function in sterol and sphingolipid localization within the plant cell. The modification of the lipid composition of the membrane may create lipid aggregations (or rafts), re-localization of proteins involved in the hypersensitive response, reduced penetration of plant cells by bacterial effectors, or lowering endocytosis of extracellular effector molecules (Borner et al., 2005; Huckelhoven, 2007; Kale et al., 2010).

The SNP ss715647322 on Pv05 associated with CBB_R1 is located 4.17 Mb from the previously identified region associated with CBB (Simons et al., 2021). The closest candidate gene (+9,399 bp) is the *Phvul.005G055800* gene model, the function of which is undetermined. The SNP ss715648956 associated with CBB_R2, located 2.16 Mb from the CBB QTL on Pv11, as previously identified by Diaz Castro (2015), is 3,806 bp upstream of the candidate gene *Phvul.011g169600*, which encodes a non-specific serine/threonine protein kinase. An additional SNP on Pv03 (slightly below Bonferroni’s threshold), associated with CBB_R2 and CBB_AUDPC, is located 0.455 Mb upstream from the previously reported QTL on this chromosome (Miklas et al., 2006). Because it is located 20.14 Mb and 24.00 Mb further from the CBB QTL, which were identified on chromosome Pv09 previously (Shi et al., 2011), the SNP ss715647179 (CBB_R2 and CBB_AUDPC) is a new genome location associated with CBB resistance. The candidate gene *Phvul.009G232500* is located 1,535 bp upstream of the SNP and encodes a beta-galactosidase 1 (carbohydrate metabolic process; glycosyl hydrolase family 35, *AT3G13750*, BGAL1) that is believed to be a plant defense enzyme (Buscaill et al., 2021). The *Arabidopsis* gene *AT3G13750* belongs to the six-member gene family encoding glycosyl hydrolase (GH) 35 involved in cell wall modification (Gantulga et al., 2009). Future work should verify the association of this region on chromosome Pv09 and involvement of the candidate gene in CBB resistance.

### 4.4 Single-marker analysis

Single-marker analysis validated the usefulness of the yield/antiyield (gene-based markers AYD1m (*Phvul.009G190100*) and AYD2m (*Phvul.009G202100*)) with yield and a few yield-related traits, including maturity, identified previously (Reinprecht et al., 2021). Both genes encode proteins with DUF1118 protein domains that are involved in the organization of light-harvesting complex II (Yokoyama et al., 2016) or function as cbZIP transcription factors that are targeted to chloroplasts and are involved in stress responses (Shahmir and Pauls, 2021). They are orthologs of *Arabidopsis AT1G74730* (RIQ2) and its homolog, *AT5G08050* (RIQ1) and *B. napus BnMicEmUP* gene (accession HQ647330). When suppressed, the *BnMicEmUP* gene had a positive effect on seed production in *Arabidopsis* and was characterized as an antiyield gene (Shahmir and Pauls, 2021). Both bean genes (*Phvul.009G190100* and *Phvul.009G202100*) are in a region of previously mapped yield and seed weight QTL on bean Pv09 (Blair et al., 2006; Mukeshimana et al., 2014; Diaz et al., 2018; Izquierdo et al., 2023). However, the current GWAS analysis conducted with the mapping panel of 121 bean genotypes and 4,485 SNPs averaged over four location/year Ontario environments did not identify associations of these markers with any of the analyzed traits. On the other hand, both analyses confirmed the effectiveness of the NPP marker in screening beans for CBB resistance. Moreover, the two analyses explained similar phenotypic variability for the CBB (30.9%–37.9% GWAS and 29.7%–37.4% single-marker analysis).

### 4.5 Study limitations and future prospects

The power of the GWAS is affected by a number of factors, including phenotypic variation, the size and structure of genotype collection, the number of markers, allele frequency, and LD (Alqudah et al., 2020). The primary limitations of the AYD_AM panel of beans for GWAS are its relatively small size, structure, and slow LD decay that might limit the resolution of association mapping. The inclusion of additional genotypes and more markers could potentially increase the mapping resolution. However, the addition of more distant genotypes would not only increase the size but also enhance differences among clusters. The prevalence of Mesoamerican genotypes in the AYD_AM panel reflects the bean breeding program at the University of Guelph, which focuses on developing high-yielding cultivars for the Ontario environment and addresses the predominance of navy beans in the mix of market classes grown in Ontario (https://ontariobeans.on.ca/). Because there were only a small number of Andean beans in the panel, LD patterns within gene pools and GWAS in separate gene pools were not considered.

However, in spite of the limitations, the work was successful in identifying a number of significant MTAs for all analyzed traits, and many of the associations confirmed previously identified genome regions associated with yield and yield-related traits in bean. Over 600 candidate genes were identified upstream and downstream of the significant MTAs. Future work should focus on elucidating their biological significance in the expression of analyzed traits. The most significant and closest associations could be starting points for the development of facile markers, such as KASP markers (Makhoul and Obermeier, 2022), that would facilitate selection for yield-related traits with the materials in the Guelph bean breeding program. In those instances where candidate genes were identified in the current study that seem to be related mechanistically to the trait being studied, such as the scarecrow-like transcription factor in harvestability, the associations are excellent starting points for developing an understanding of causation of the trait.

In conclusion, this study offers insights into bean diversity and provides valuable information to bean breeders and geneticists, which can be used in bean toward cultivar improvement. The SNPs associated with yield and yield-related traits, including CBB resistance, can aid in the understanding of the genetic architecture of these traits, as well as simplify breeding for high-yielding and CBB-resistant bean cultivars.

## Data Availability

The original contributions presented in the study are publicly available. These data can be found at: https://borealisdata.ca/dataset.xhtml?persistentId=doi:10.5683/SP3/FD81LR.

## Supplementary material

SUPPLEMENTARY FIGURE S1 Images of the 121 common bean genotypes selected for the yield/antiyield association mapping (AYD_AM) panel.

SUPPLEMENTARY FIGURE S2 Year of release of common bean cultivars/lines selected for the yield/antiyield (AYD_AM) study and their yields (averaged for Elora, Woodstock, St Thomas, and Monkton, ON, for years 2010–2014).

SUPPLEMENTARY FIGURE S3 Weather conditions at the University of Guelph Elora (ERS) and Woodstock research stations (WRS) (Ontario) in 2015 and 2016 common bean growing seasons.

SUPPLEMENTARY FIGURE S4 A scree plot of the eigenvalues of principal components in a principal component analysis (PCA) of traits evaluated in the 121 AYD_AM collection of common beans averaged over four Ontario location/year environments.

SUPPLEMENTARY FIGURE S5 Genome distribution of the SNPs used in the common bean AYD_AM study. **(A)** SNP distribution before and after site-filtering (Tassel 5). SNPs with unknown genome positions (107) were removed from the analysis. **(B)** Relationships between the number of SNPs and chromosome sizes. **(C)** SNP densities within 1-Mb windows across the genome. The horizontal axis shows the physical distance, with the different colors reflecting the local SNP densities. **(D)** Average SNP densities in chromosomes. The bubble size indicates the number of SNPs per kilobase (Kb) for each chromosome (values in brackets).

SUPPLEMENTARY FIGURE S6 Physical locations of 4,485 SNPs in the *Phaseolus vulgaris* v2.1 genome sequence (https://phytozome-next.jgi.doe.gov) used to map yield and yield-related traits in the AYD_AM collection of common beans evaluated over four Ontario location/year environments.

SUPPLEMENTARY FIGURE S7 Structure of the AYD_AM panel of common beans based on an analysis performed with 220 SNPs, with K=2.

SUPPLEMENTARY FIGURE S8 Linkage disequilibrium decay rate by chromosome in common beans evaluated with the 4,485 SNPs in the AYD_AM collection of 121 genotypes.

SUPPLEMENTARY FIGURE S9 GWAS model selection based on 4,485 SNPs for 121 common bean genotypes in GAPIT 3. **(A)** A scree plot was used to select the number of principal components (PCs) to retain in the analysis. It shows the eigenvalues (variance) on the y-axis and the number of PCs on the x-axis. The leveling off of the curve indicates that the three PCs should be kept in the analysis. **(B)** A 3D scatter plot of the first three PCs. **(C)**)Heatmap of pairwise kinship matrix values generated with the VanRaden algorithm (VanRaden, 2008). The color histogram shows the distribution of the coefficients of the coancestry. A stronger red color indicates more related genotypes (all created in GAPIT 3).

SUPPLEMENTARY FIGURE S10 Quantile–quantile (Q-Q) plots from the initial GWAS scan with multiple models [General Linear Model (GLM), Mixed Linear Model (MLM), Multiple Locus Mixed linear Model (MLMM), Fixed and random model Circulating Probability Unification (FarmCPU), and Bayesian-information and linkage-disequilibrium Iteratively Nested Keyway (BLINK)] for 15 traits averaged over four Ontario location/year environments conducted using GAPIT 3 software. The shaded areas indicate the 95% confidence intervals. For most traits in the BLINK models, the majority of the *p*-values were similar to the expected *p*-values (diagonal in the Q-Q plot), which indicated the appropriateness of this model for the GWAS analysis. Measured traits: YD = yield (kg ha^-1^), SW = seed weight (g), DF = days to flowering (days), DM = days to maturity (days), PH = plant height (cm), HR = harvestability (scale 1–5), CBB_R1 = common bacterial blight (scale 0–5) 1^st^ scoring, CBB_R2 = 2^nd^ CBB scoring, and CBB_AUDPC = CBB area under the disease progress curve. Derived traits: RP = reproductive period (RP = DM–DF, days), YGD = yield gain per day (YGD = YD/DM, kg ha^-1^ day^-1^), SGR = seed growth rate (SGR = YD/RP, kg ha^-1^ day^-1^), YDH = yield per unit of height (YDH = YD/PH, kg ha^-1^ cm^-1^), SN = seed number (SN = YD/SW, seed number x 10^6^ seeds ha^-1^), and YDHR = yield per unit of harvestability (YDHR = YD/HR, kg ha^-1^).

SUPPLEMENTARY FIGURE S11 Complete physical map with positions of significant marker-trait associations (MTAs) identified in the current AYD_AM study and QTL reported previously for yield and yield-related traits in the common bean genome sequence (*Phaseolus vulgaris* v2.1). Details can be found in Figure 4.
