# Supplementary figures and images for: Mapping yield and yield-related traits using diverse common bean germplasm
Source: Front Genet. 2024 Jan 3;14:1246904. doi: 10.3389/fgene.2023.1246904 (PMC10791882; doi:10.3389/fgene.2023.1246904)

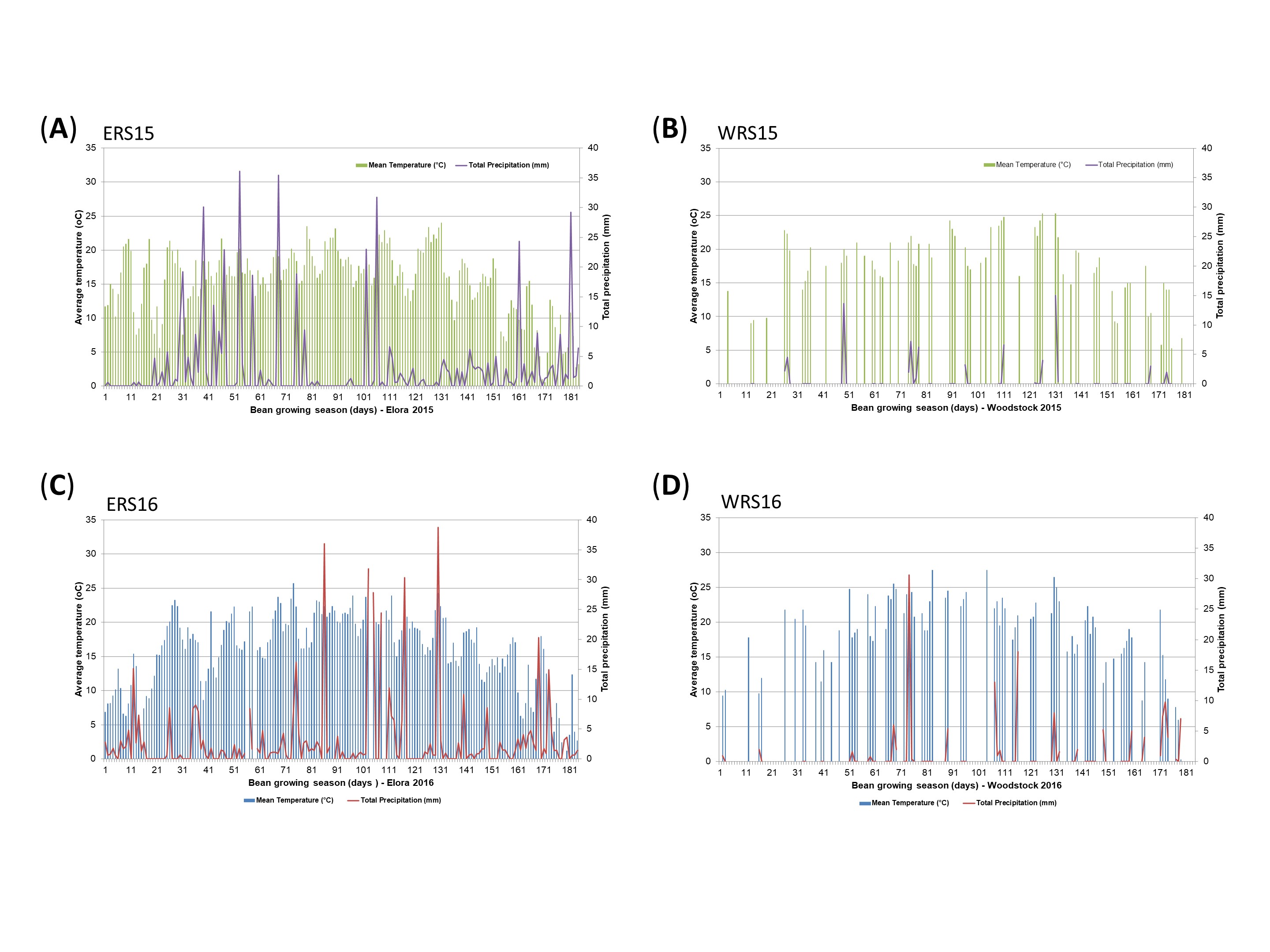

Supplement: Supplementary file 1 [file Image3.JPEG]

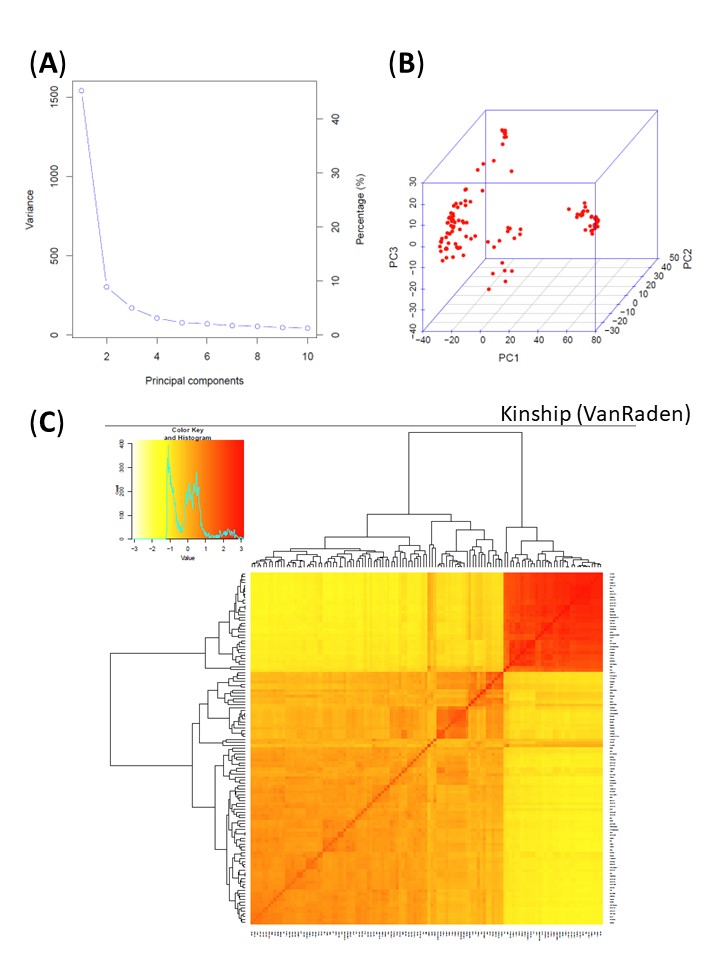

Supplement: Supplementary file 3 [file Image9.JPEG]

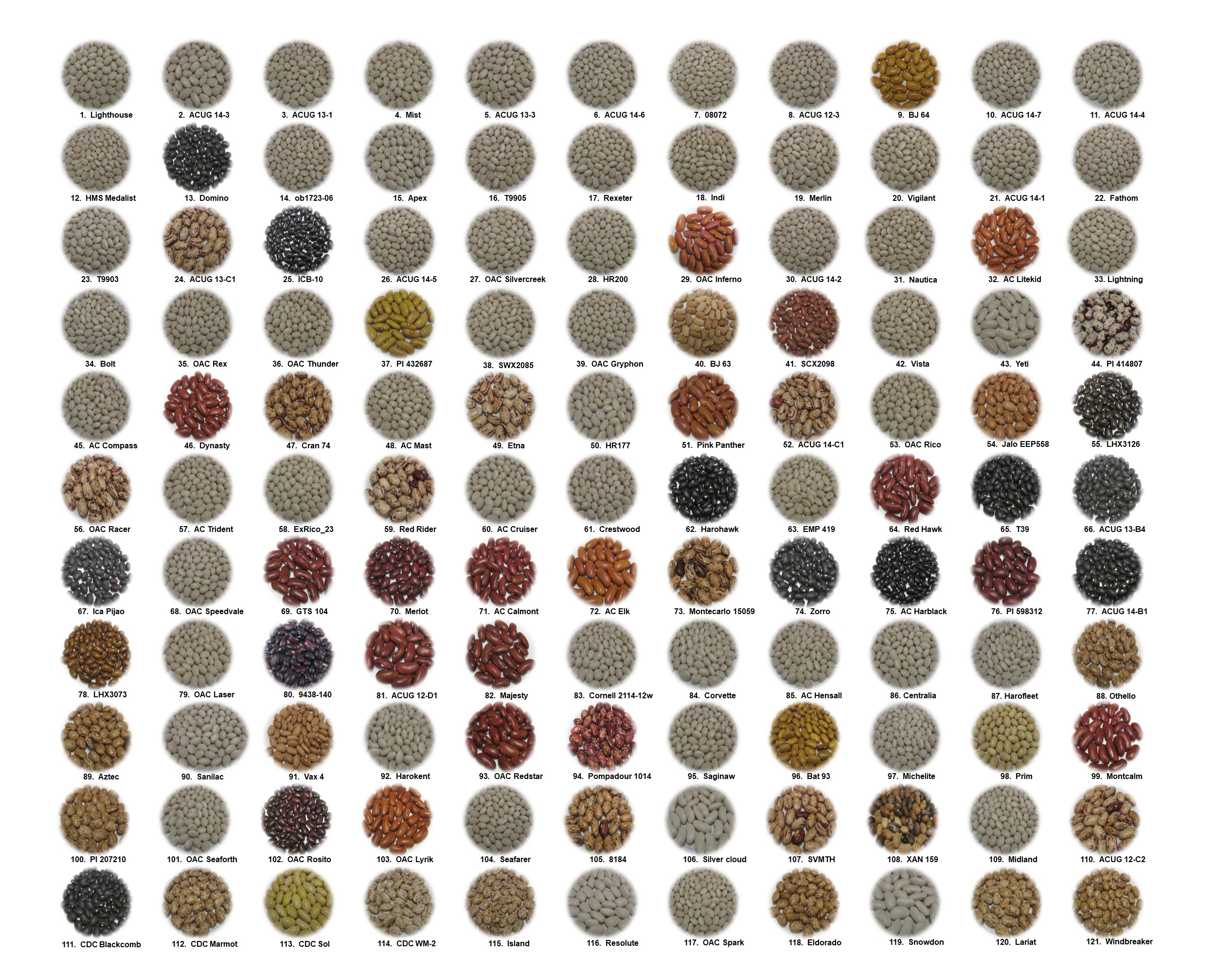

Supplement: Supplementary file 6 [file Image1.JPEG]

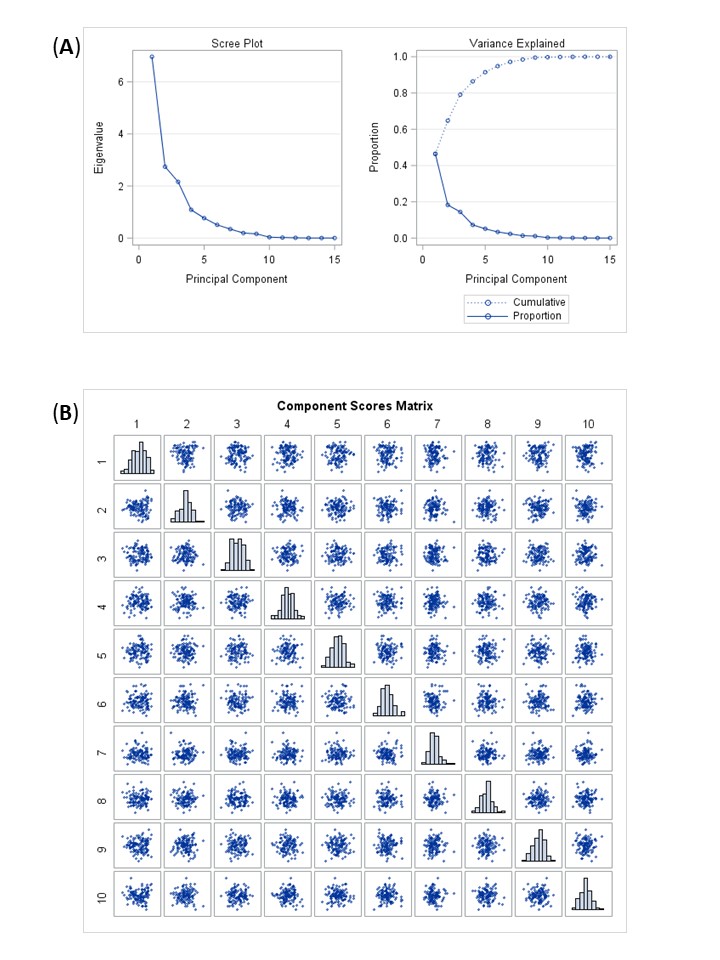

Supplement: Supplementary file 7 [file Image4.JPEG]

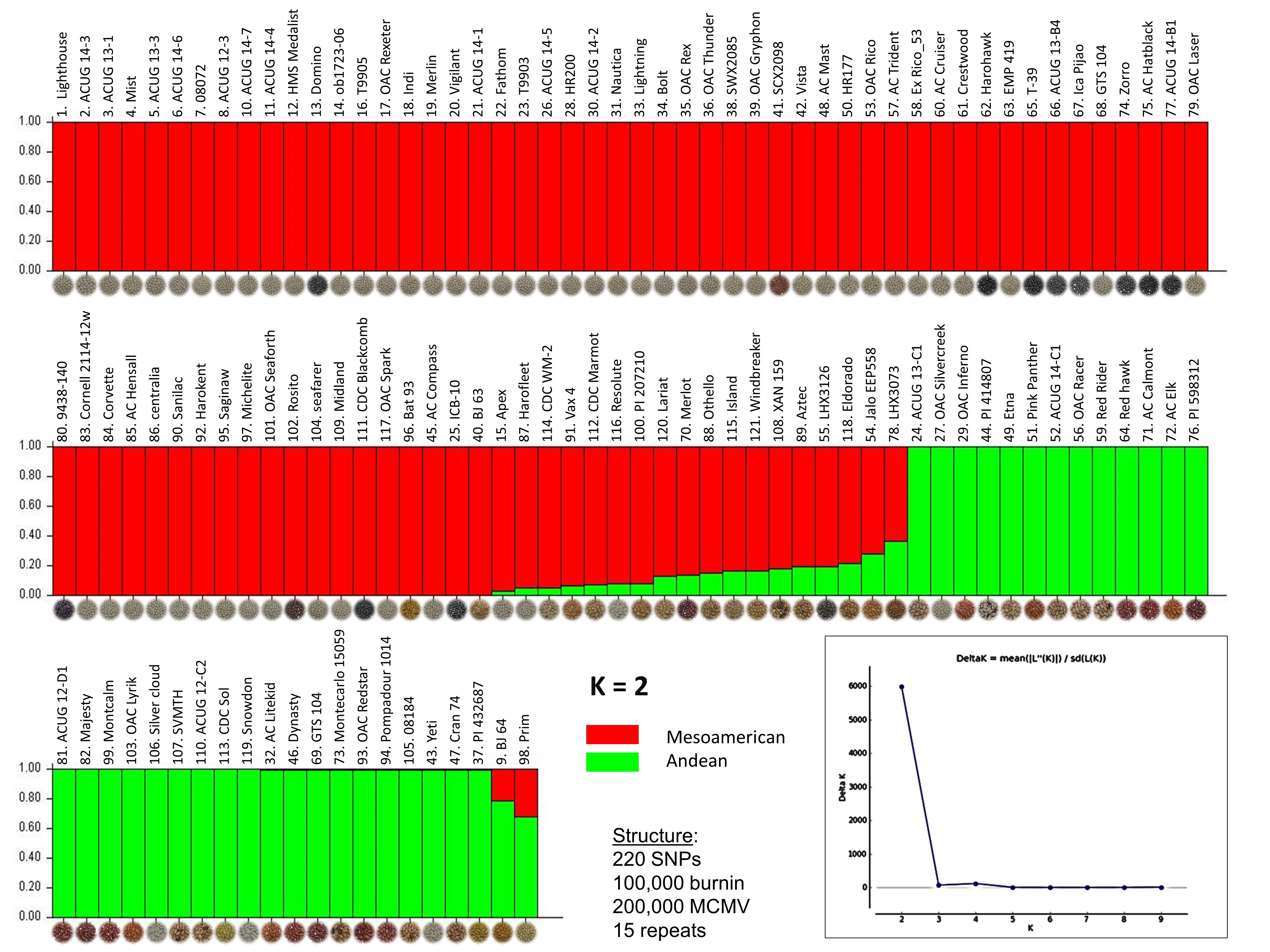

Supplement: Supplementary file 8 [file Image7.JPEG]

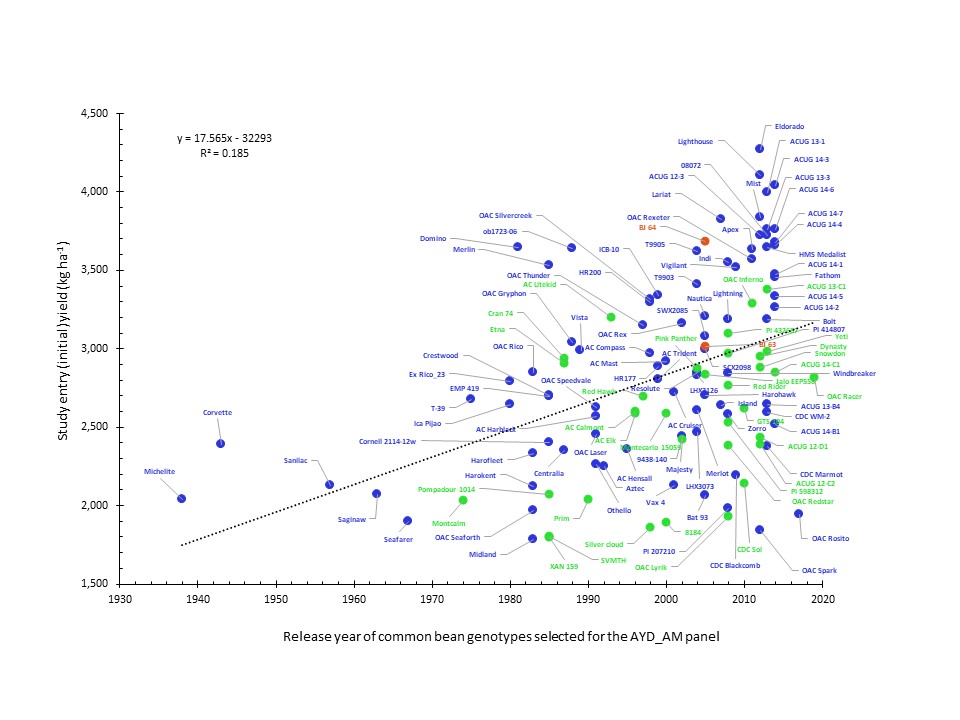

Supplement: Supplementary file 9 [file Image2.JPEG]

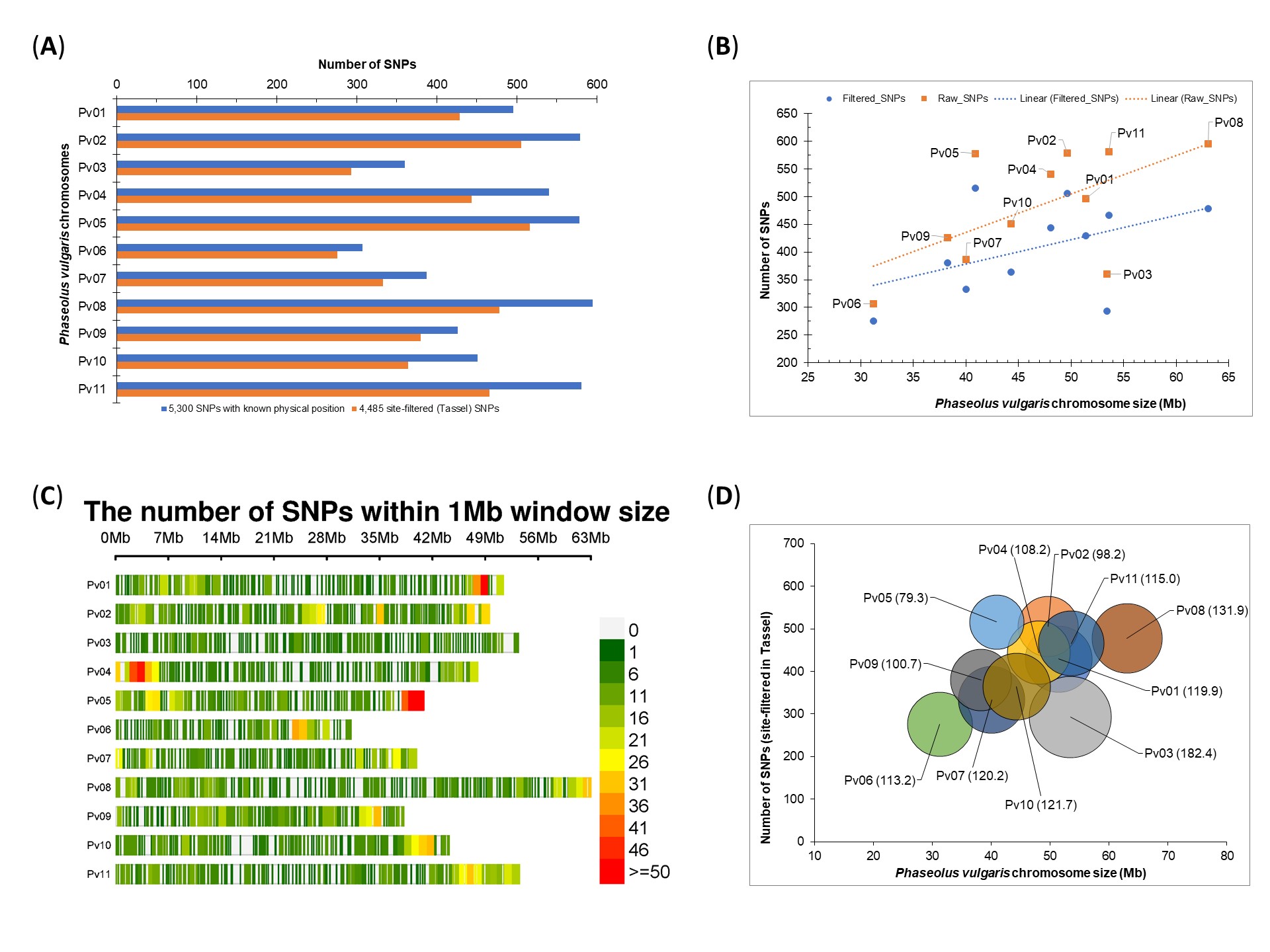

Supplement: Supplementary file 10 [file Image5.JPEG]

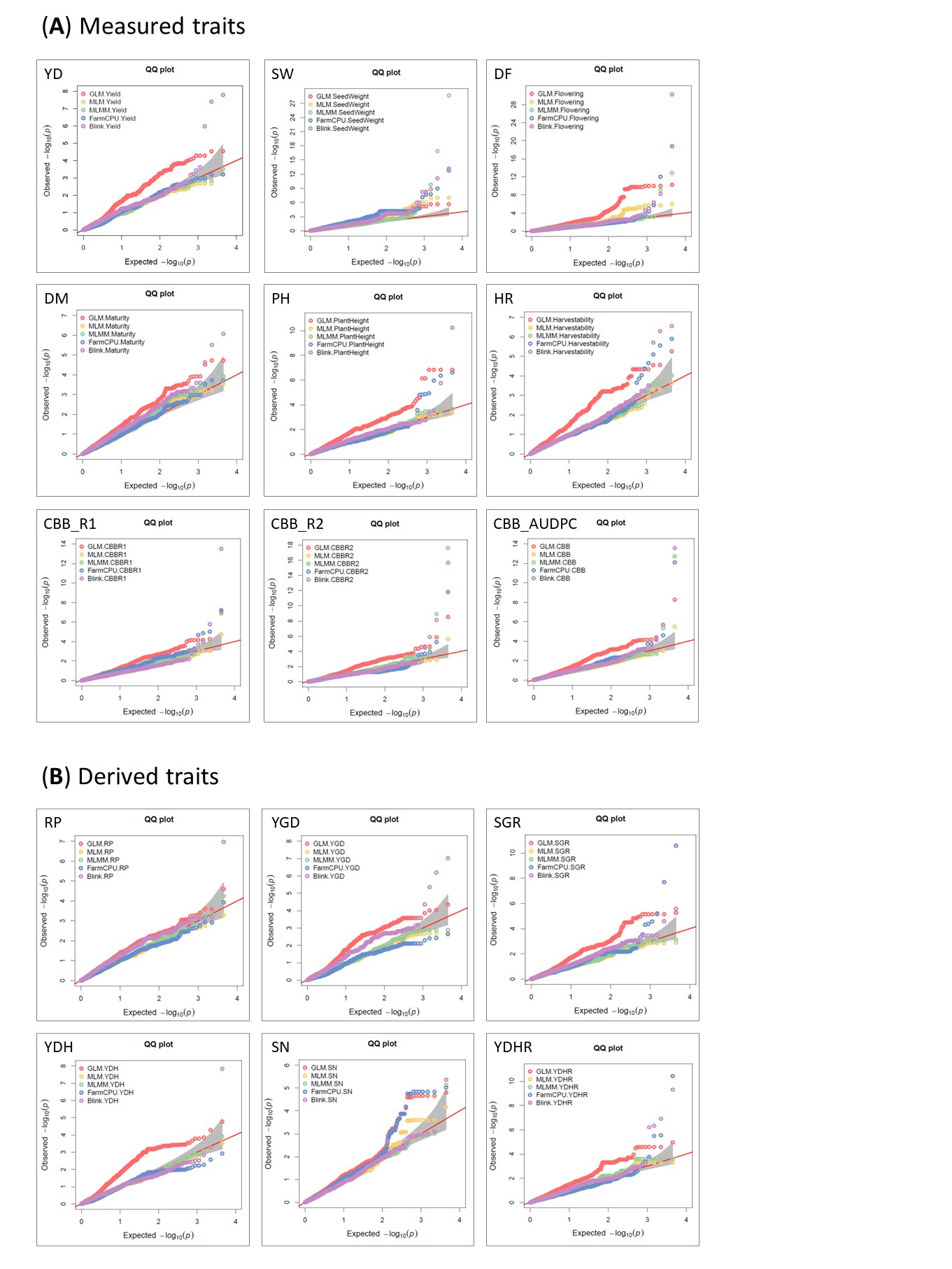

Supplement: Supplementary file 11 [file Image10.JPEG]

Pv01

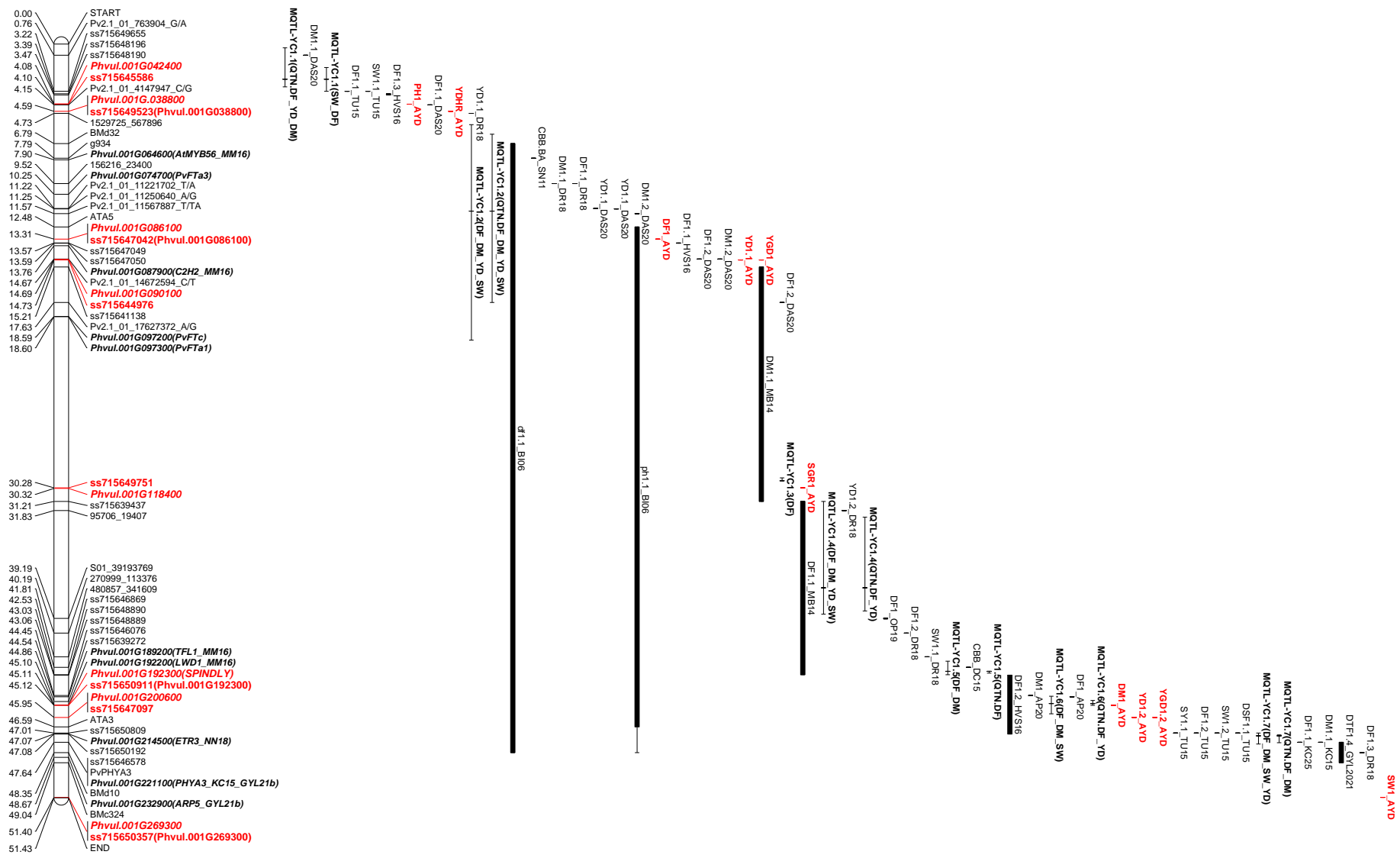

**Pv02**

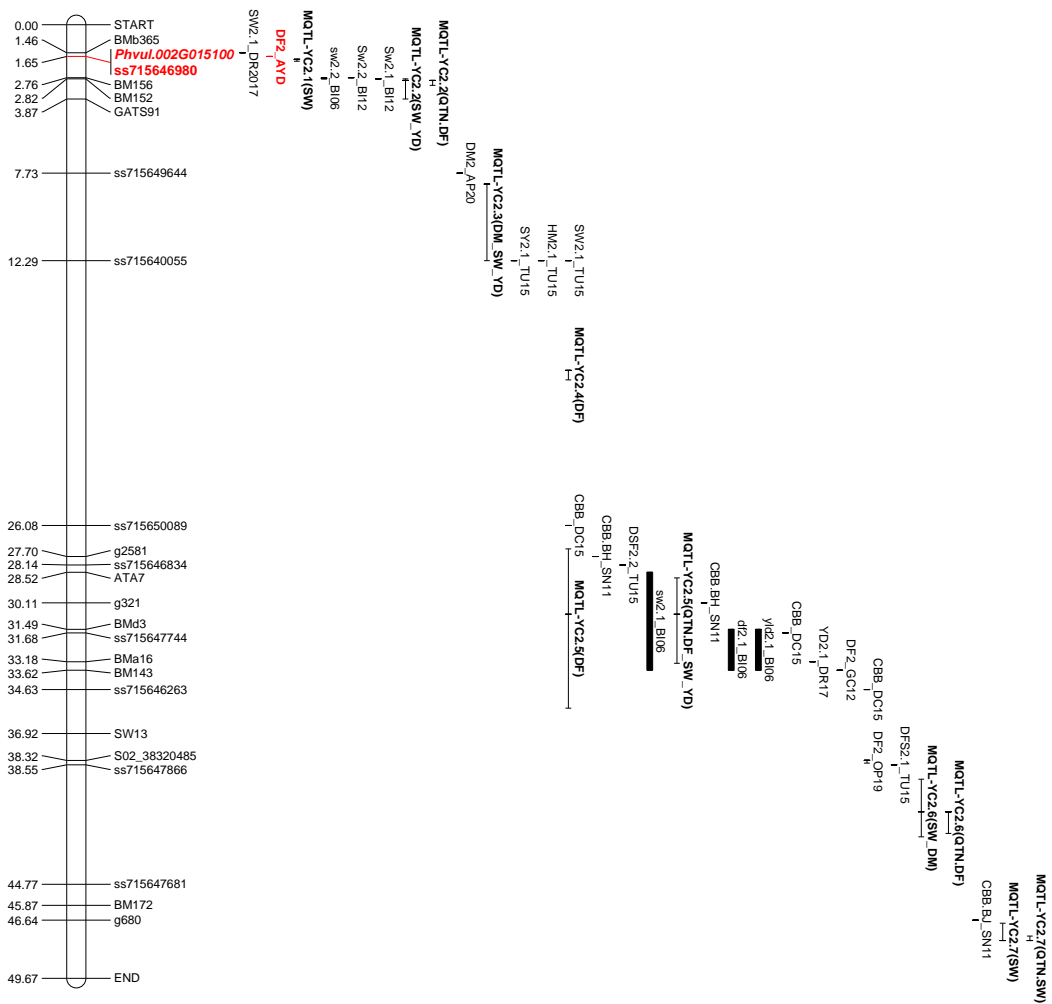

# Pv03

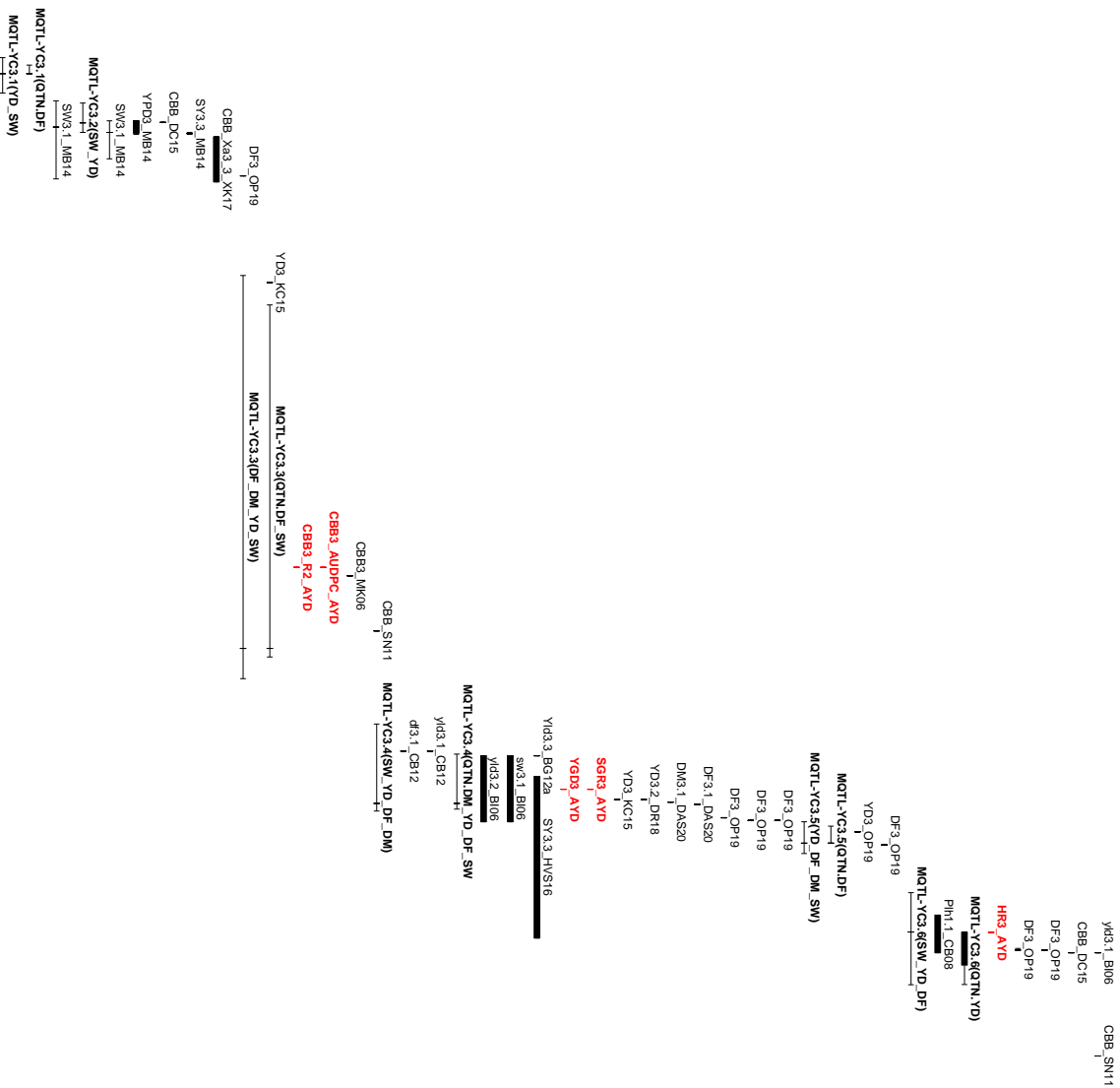

Pv04

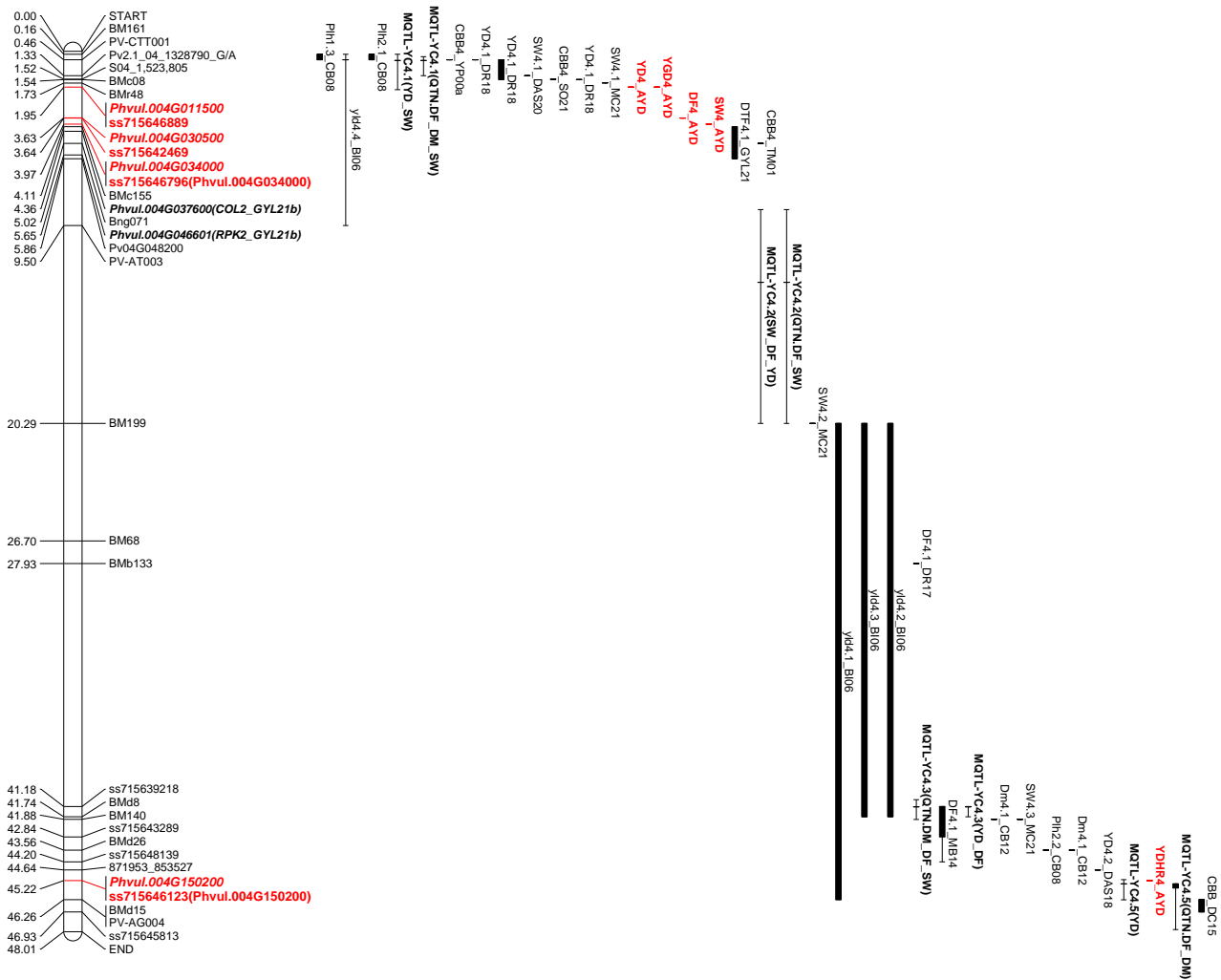

# Pv05

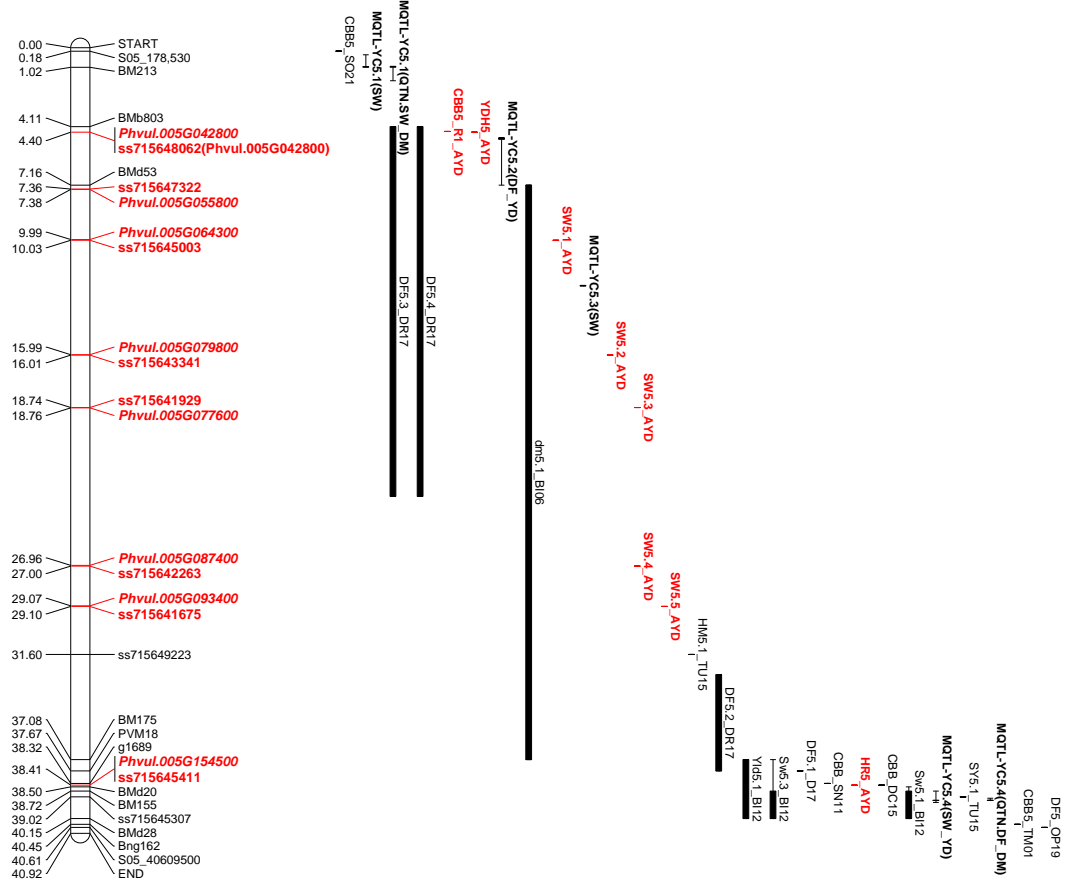

## Pv06

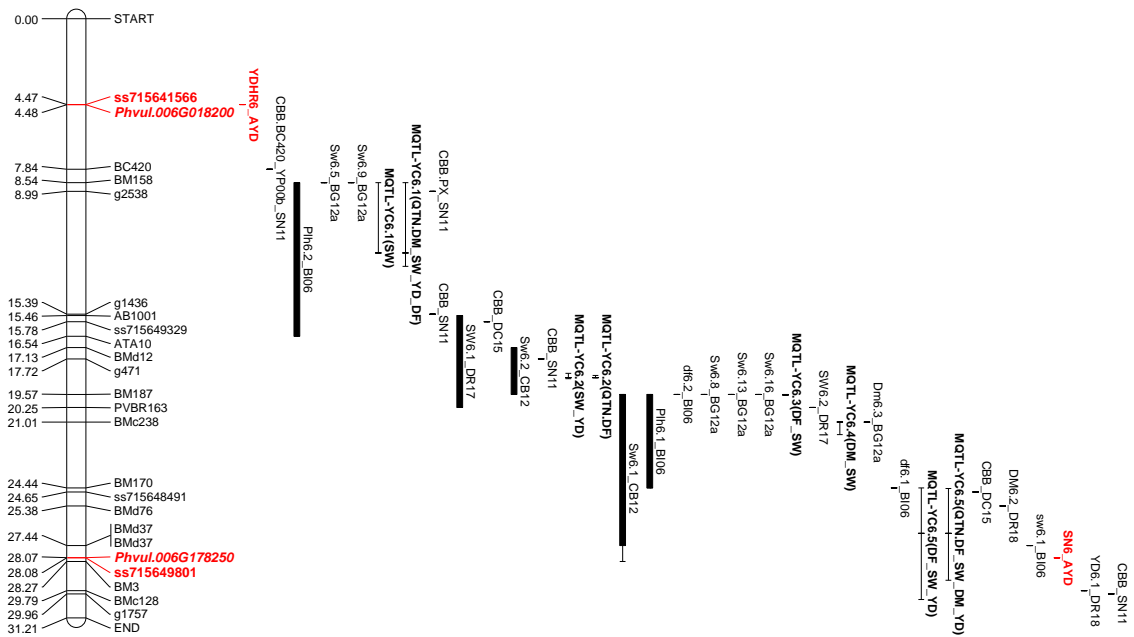





**Pv09**

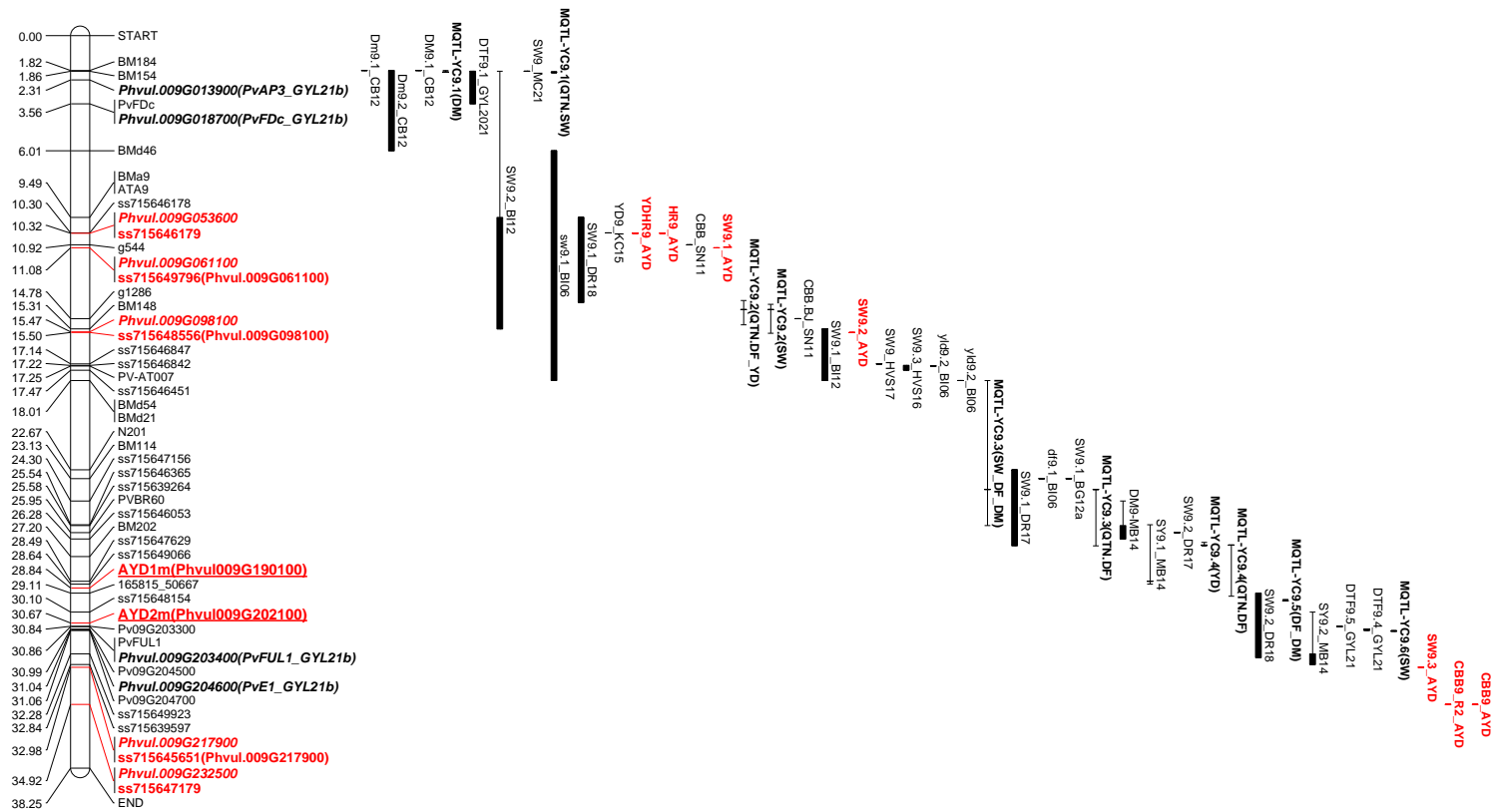

## Pv10

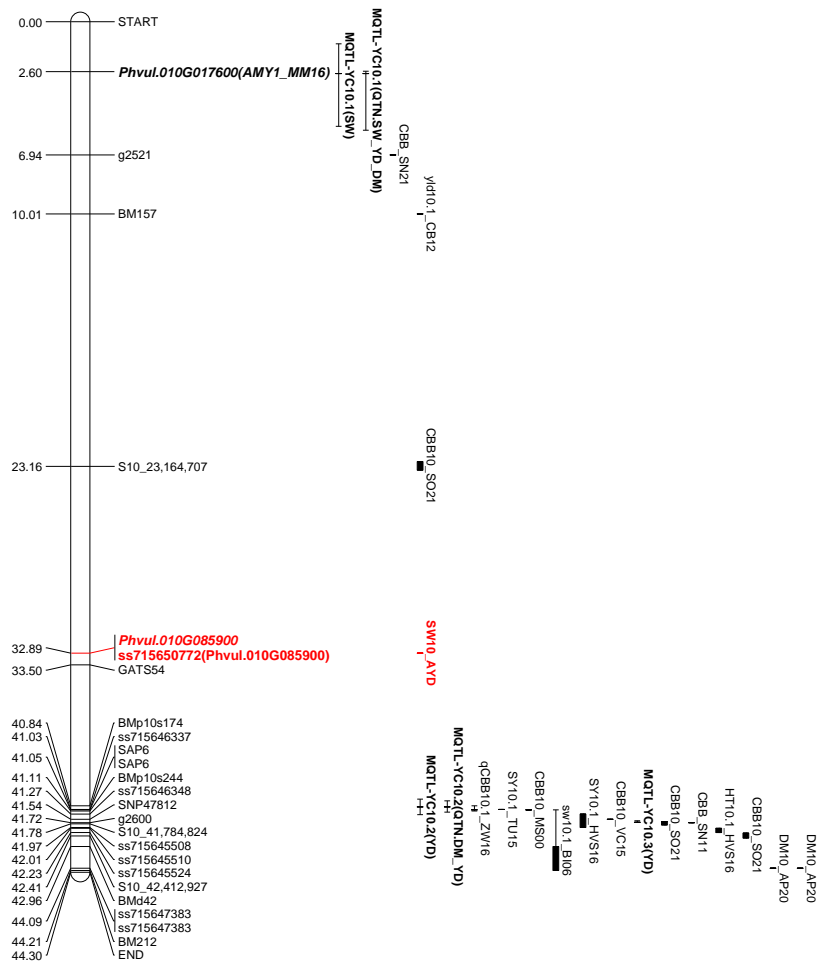

# Pv11

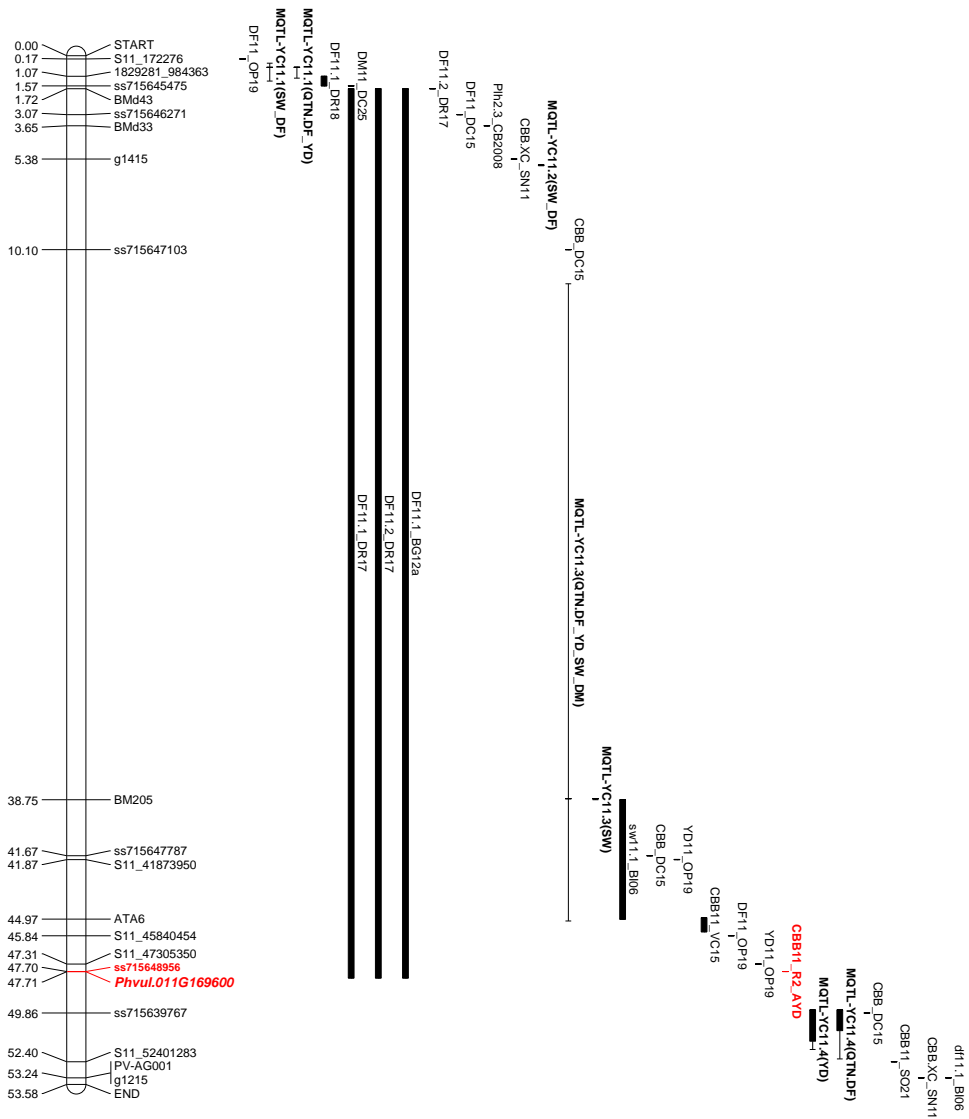

Supplement: Supplementary file 14 [file DataSheet1.PDF]

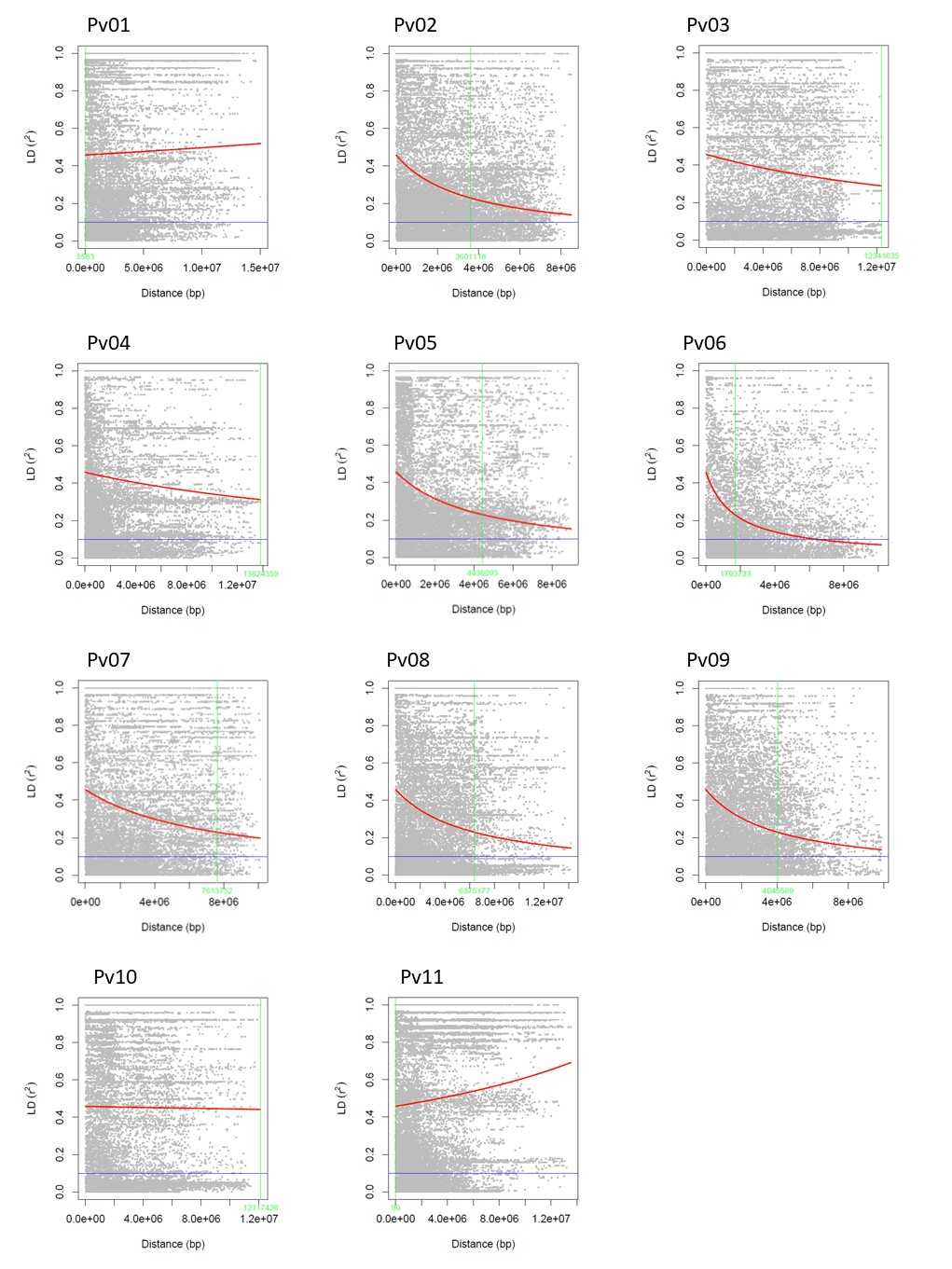

Supplement: Supplementary file 18 [file Image8.JPEG]

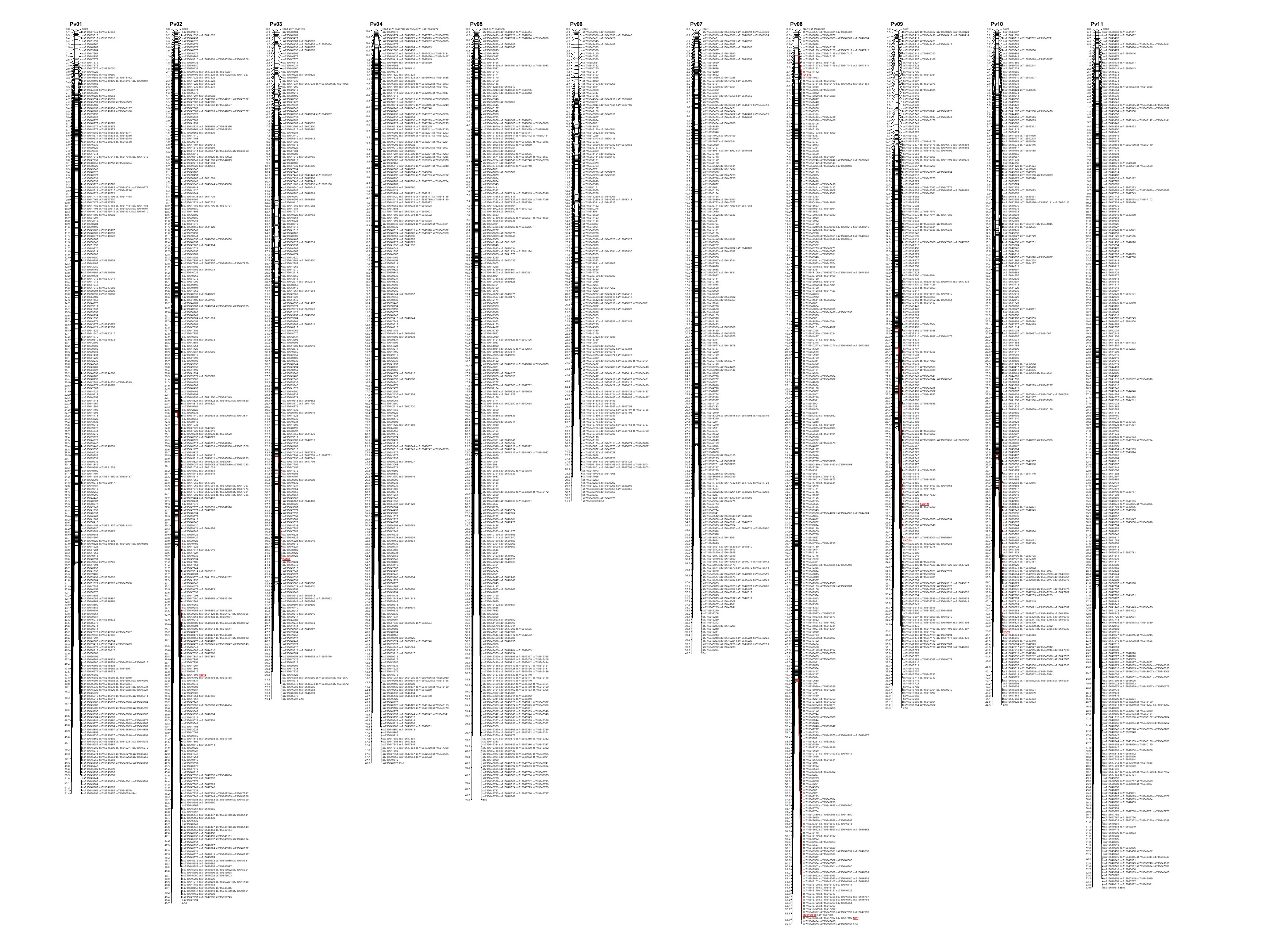

Supplement: Supplementary file 21 [file Image6.JPEG]
