# Supplementary material for: Mapping yield and yield-related traits using diverse common bean germplasm
Source: Front Genet. 2024 Jan 3;14:1246904. doi: 10.3389/fgene.2023.1246904 (PMC10791882; doi:10.3389/fgene.2023.1246904)
Supplement: Supplementary file 2 [file Table1.DOCX]

**Supplementary Table S1** **|** Characteristics of 121 common bean genotypes selected for the anti-yield association mapping (AYD_AM) panel.

| **Entry^1^** | **Germplasm** | **Previous name** | **Pedigree** | **Origin** | **Year of release^2^** | **Gene**  **pool^3^** | **Race^4^** | **Market class** | **Yield^5^**  **(kg ha^-1^)** |
| --- | --- | --- | --- | --- | --- | --- | --- | --- | --- |
| 76 | PI 598312 | HOK8-1 | NA | Japan | 2008 | A | - | (Kintoki) | 2,528 |
| 44 | PI 414807 | Lady bean | NA | Hungary | 2008 | A | - | (Purple Mottled) | 2,971 |
| 94 | Pompadour 1014 | NA | NA | NA | 1985 | A | - | (Purple Mottled) | 2,072 |
| 108 | XAN 159 | NA | UI-114/PI319441//PI319443/3/Masterpiece; derived from *P. acutifolius* (G 40020 = PI 319443) | Colombia - CIAT | 1985 | A | - | (Xan) | 1,794 |
| 98 | Prim | NA | NA | UK | 1990  (1998) | A | - | Yellow | 2,039 |
| 113 | CDC Sol | 2253-4 | Monteca F//CPC-00-250/610-23 | Canada | 2010 | A | - | Yellow | 2,140 |
| 47 | Cran 74 | NA | NA | USA | 1987 | A | NG | Cranberry | 2,941 |
| 49 | Etna | XPB 213 | Taylor Hort/Mosaic resistant Cranberry breeding line (BCMV_R) | USA | 1987  (2006) | A | NG | Cranberry | 2,906 |
| 52 | ACUG 14-C1 | W11BA019 | HR163/Etna | Canada | 2014 | A | NG | Cranberry | 2,851 |
| 56 | OAC Racer | W11CE100/ ACUG 14-C2 | HR161/Etna | Canada | 2019 | A | NG | Cranberry | 2,818 |
| 59 | Red Rider | HR163-4099 | SVMTH/Dolly | Canada | 2008 | A | NG | Cranberry | 2,765 |
| 73 | Montecarlo 15059 | NA | NA | NA | 2000 | A | NG | Cranberry | 2,585 |
| 105 | 8184 | NA | NA | USA | 2000 | A | NG | Cranberry | 1,894 |
| 107 | SVMTH | SVM CRAN SE1 | NA | USA | 1985  (1992) | A | NG | Cranberry | 1,800 |
| 110 | ACUG 12-C2 | H5357C-42108 | HR167-4099/Chianti | Canada | 2012 | A | NG | Cranberry | 2,390 |
| 24 | ACUG 13-C1 | NA | Etna/H4607-25386 [(HR48LRK/Cran09)/Ferg. Cran | Canada | 2013 | A | NG | Cranberry (Romano) | 3,378 |
| 46 | Dynasty | OAC 07-6D1 | HR85-1885 and Montcalm and USWA-39 and AC Litekid | Canada | 2012 | A | NG | Dark Red Kidney (DRK) | 2,953 |
| 69 | GTS 104 | NA | NA | Canada | 2010 | A | NG | DRK | 2,617 |
| 64 | Red Hawk | K90101 | Charlevoix/2*Montcalm | USA | 1997  (2003) | A | NG | DRK | 2,697 |
| 71 | AC Calmont | HR41-923 | California Light Red Kidney (CLRK)/Montcalm | Canada | 1996 | A | NG | DRK | 2,603 |
| 81 | ACUG 12-D1 | H5334B | Majesty//Majesty/(Majesty///ELK//OAC Rex/MM4) | Canada | 2012 | A | NG | DRK | 2,434 |
| 82 | Majesty | HR111-1889 | Montcalm/DRK15 | Canada | 2002 | A | NG | DRK | 2,422 |
| 93 | OAC Redstar | OAC 03-D1 | Montcalm/Litekid//Cran 09/Foxfire///Chinook/Darkid//Generatif/OAC 90-C1 | Canada | 2008 | A | NG | DRK | 2,383 |
| 99 | Montcalm | DRK023 | Charlevoir DRK/Great Northern # 1 | USA | 1974  (1998) | A | NG | DRK | 2,034 |
| 29 | OAC Inferno | OAC 07-L1 | HR85-1885/Montcalm//USWA-39/AC Litekid///Foxfire/  AC Elk//Sacramento/AC Calmont | Canada | 2011 | A | NG | Light Red Kidney (LRK) | 3,291 |
| 32 | AC Litekid | HR24-942 | Ruddy/CDRK | Canada | 1993 | A | NG | LRK | 3,199 |
| 51 | Pink Panther | EX 08590462 | Inbred line (parentage not disclosed) | USA | 2004 | A | NG | LRK | 2,872 |
| 72 | AC Elk | HR48-1290 | Mecosta/MRK44 | Canada | 1996 | A | NG | LRK | 2,586 |
| 103 | OAC Lyrik | OAC 04-L1 | AC Elk/AC Litekid | Canada | 2008 | A | NG | LRK | 1,932 |
| 54 | Jalo EEP558 | NA | A selection from Brazilian landrace Jalo | Brazil | 2005 | A | NG | (Tan Kidney) | 2,837 |
| 106 | Silver cloud | USWA-70 | Lisa/Linden | USA | 1998 | A | NG | White Kidney | 1,858 |
| 119 | Snowdon | K08961 | K04604/USDK-CBB-15 | USA | 2012 | A | NG | White Kidney | 2,881 |
| 43 | Yeti | ACUG 10-W1 | AC Calmont/PI358207 | Canada | 2013 | A | NG | White Kidney (Canellini) | 2,982 |
| 37 | PI 432687 | Strogele | NA | Netherlands | 2008 | A | NG | Yellow | 3,098 |
| 40 | BJ 63 | NA | Bat 93/JaloEEP558 | USA | 2005 | AM | - | Tan | 3,013 |
| 9 | BJ 64 | NA | Bat 93/Jalo EEP558 | USA | 2005 | AM | - | Yellow | 3,686 |
| 91 | Vax 4 | PI 613174 | NA | Colombia - CIAT | 2001 | M | - | (Tan) | 2,125 |
| 96 | Bat 93 | NA | (G3709 x G1320) x (G3645 x G5478) | Colombia - CIAT | 2005 | M | - | (Tan) | 2,063 |
| 116 | Resolute | L98E212 | 83B352/5/GN Star*2/ 3/Redkloud/Kentwood/ 2/Swan Valley/4/GN Star*2 /3/Redkloud/Kentwood/ 2/Swan Valley/ 6/Sask 92070 | Canada | 2004 | M | D | Great Northern | 2,851 |
| 88 | Othello | GH-215 | Pinto NW-410/2/Pink Victor/Aurora | USA | 1991 | M | D | Pinto | 2,263 |
| 89 | Aztec | MSU#P89430 | CO81-12034/P86297 | USA | 1992 | M | D | Pinto | 2,250 |
| 100 | PI 207210 | Idaho pinto | NA | Colombia - CIAT | 2008 | M | D | Pinto | 1,982 |
| 112 | CDC Marmot | 2537-12 | SC11745-3/CDC Pintius | Canada | 2013 | M | D | Pinto | 2,374 |
| 114 | CDC WM-2 | 2793 CBB | Minto/3/Minto/Weihing//Minto/OAC Rex/4/1533-15*3/6/Minto*2/5/1533-15/4/Minto/3/Minto/Weihing//Minto/OAC Rex | Canada | 2013 | M | D | Pinto | 2,596 |
| 115 | Island | L03B754 | 64-68-8/6/PINTO 410*2/3/'REDKL'/'Kentwood'/2/'S VALLEY/4/SASK_92064 | Canada | 2007 | M | D | Pinto | 2,640 |
| 118 | Eldorado | MSU#P07863 | AN-37/P02630 | USA | 2012 | M | D | Pinto | 4,270 |
| 120 | Lariat | ND020069 | ‘Maverick’, ‘Winchester’ and ‘Aztec’ along with numerous experimental lines | USA | 2007 | M | D | Pinto | 3,826 |
| 121 | Windbreaker | NA | Buster/Matterhorn | USA | 2008 | M | D | Pinto | 2,844 |
| 13 | Domino | MSU#61380 | NEP-2/Black Turtle Soup (BTS) | USA | 1981 | M | M | Black | 3,646 |
| 25 | ICB-10 | VCV-II-10,19322-256b, H9652-5,  ICB-10-5 | *P. vulgaris/P. coccineus//233B)* | USA | 1999 | M | M | Black | 3,339 |
| 55 | LHX3126 | JL157 | Berna/EMP419 | Canada | 2004 | M | M | Black | 2,832 |
| 62 | Harohawk | HR123-2642B | Raven//HR45/AC Harblack | Canada | 2005 | M | M | Black | 2,701 |
| 65 | T-39 | NA | NA | USA | 1975 | M | M | Black | 2,675 |
| 66 | ACUG 13-B4 | W10BE080 | RWX4035/Rex | Canada | 2013 | M | M | Black | 2,644 |
| 67 | Ica Pijao | NA | NA | Colombia - ICA | 1980 | M | M | Black | 2,644 |
| 74 | Zorro | B04554 | B00103*2/X00822 | USA | 2008  (2012) | M | M | Black | 2,579 |
| 75 | AC Harblack | HR21-893 | Snowflake/Midnight | Canada | 1991 | M | M | Black | 2,565 |
| 77 | ACUG 14-B1 | W11BB112 | Common Red Mex/RWX4018b | Canada | 2014 | M | M | Black | 2,516 |
| 78 | LHX3073 | JL81 | Berna/EMP419 | Canada | 2004 | M | M | Black | 2,468 |
| 111 | CDC Blackcomb | 1519-10 | NA | Canada | 2009 | M | M | Black | 2,191 |
| 2 | ACUG 14-3 | 47180 | Rex/SWX2045 | Canada | 2014 | M | M | Navy | 4,040 |
| 3 | ACUG 13-1 | GF6-11-8 | RWX4041/Rexeter | Canada | 2013 | M | M | Navy | 3,995 |
| 4 | Mist | ACUG 10-6 | spscbbr136/PI207262//ICB-10/Vax4///OAC Speedvale/Avanti//OAC 99-1/OAC Rex | Canada | 2012 | M | M | Navy | 3,839 |
| 5 | ACUG 13-3 | W10WA131 | RWX4042/Rexeter | Canada | 2013 | M | M | Navy | 3,763 |
| 6 | ACUG 14-6 | W11WB018 | HR135/SWX2045 | Canada | 2014 | M | M | Navy | 3,759 |
| 7 | 08072 | NA | NA | NA | 2013 | M | M | Navy | 3,723 |
| 8 | ACUG 12-3 | H5348-43297 | HR164/(HR164//AC Compass/MM16) | Canada | 2012 | M | M | Navy | 3,721 |
| 10 | ACUG 14-7 | W11WA174 | HR135/SWX2045 | Canada | 2014 | M | M | Navy | 3,678 |
| 11 | ACUG 14-4 | 47393 | HR144/w4170a-96060(HR93/OAC Rex) | Canada | 2014 | M | M | Navy | 3,659 |
| 12 | HMS Medalist | 1054 | NA | USA | 2013 | M | M | Navy | 3,648 |
| 14 | ob1723-06 | NA | NA | NA | 1988 | M | M | Navy | 3,638 |
| 15 | Apex | HR199-4587 | Centralia/NY5268//HR67/3/AC Cruiser | Canada | 2011 | M | M | Navy | 3,631 |
| 16 | T9905 | 151-971 | Vista/D80024 | Canada | 2004 | M | M | Navy | 3,618 |
| 17 | OAC Rexeter | OAC 07-2 | OAC Rex/AC Kippen | Canada | 2011 | M | M | Navy | 3,572 |
| 18 | Indi | N5027557 | NA | NA | 2008  (2012) | M | M | Navy | 3,553 |
| 19 | Merlin | 3019 | NA | NA | 1985 | M | M | Navy | 3,532 |
| 20 | Vigilant | 2084 | NA | USA | 2009 | M | M | Navy | 3,520 |
| 21 | ACUG 14-1 | 47422 | HR144/w4170a-96060(HR93/OAC Rex) | Canada | 2014 | M | M | Navy | 3,473 |
| 22 | Fathom | ACUG 12-5 | spse087/spsbf019 | Canada | 2014 | M | M | Navy | 3,456 |
| 23 | T9903 | 89-967 | Pilot/HR20-728 | Canada | 2004 | M | M | Navy | 3,407 |
| 26 | ACUG 14-5 | W11WE153 | BLTXX161/SWX2045 | Canada | 2014 | M | M | Navy | 3,335 |
| 27 | OAC Silvercreek | OAC 94-3 | Midnight/Seafarer//Cran 74 | Canada | 1998 | M | M | Navy | 3,317 |
| 28 | HR200 | HR200-4345 | [(L9322/OAC 87-2)/Vista]/OAC 95-4 | Canada | 1998 | M | M | Navy | 3,292 |
| 30 | ACUG 14-2 | W11WE059 | OAC Spark/BLTWXX161 | Canada | 2014 | M | M | Navy | 3,265 |
| 31 | Nautica | HR81-5 | OAC Laser/HR20-827 | Canada | 2005 | M | M | Navy | 3,208 |
| 33 | Lightning | OAC 04-2 | Navigator/ OAC Thunder//AC Compass/ OAC Silvercreek | Canada | 2008 | M | M | Navy | 3,187 |
| 34 | Bolt | ACUG 10-1 | OAC Rex/A98083//AC Compass/B98143///RESW2138/  B981045//B98213/Kippen | Canada | 2013 | M | M | Navy | 3,186 |
| 35 | OAC Rex | OAC 95-4 | (HR20-728///ICA Pijao/PI 440795 (*P. acutifolius*)//Ex Rico 23 | Canada | 2002 | M | M | Navy | 3,163 |
| 36 | OAC Thunder | OAC 93-1 | ExRico 23 *5 // Ex Rico23 / Narda/4/T81-01 / Domino // Ex Rico are / Domino /3/ NZUpright / Sacremento Light Red Kidney | Canada | 1997 | M | M | Navy | 3,146 |
| 38 | SWX2085 | NA | Silvercreek/CIAT9 | Canada | 2005 | M | M | Navy | 3,077 |
| 39 | OAC Gryphon | OAC 82-2 | Ex Rico 23/Narda//5*ExRico 23 | Canada | 1988 | M | M | Navy | 3,039 |
| 42 | Vista | XPB 190 | GTS 0181-1/Seafarer | Canada | 1989 | M | M | Navy | 2,990 |
| 45 | AC Compass | HR70-1774 | Crestwood/HR14//OAC Laser | Canada | 1998 | M | M | Navy | 2,968 |
| 48 | AC Mast | HR80-1439 | HR-13-621//OAC Rico/XAN 159 | Canada | 2000 | M | M | Navy | 2,918 |
| 50 | HR177 | HR177-3719 | (OAC 87-2/HR20-827)/OAC 94-3 | Canada | 1999 | M | M | Navy | 2,889 |
| 53 | OAC Rico | Ex-Rico-*Are* (OAC 81-4) | Ex Rico 23*6/Ex-Rico 23/Narda | Canada | 1983 | M | M | Navy | 2,850 |
| 57 | AC Trident | HR74-1756 | OAC Gryphon/OAC Laser | Canada | 1999 | M | M | Navy | 2,804 |
| 58 | Ex Rico_23 | ICA Bunsi | Magdalena/Japon3 | Colombia - CIAT/ ICA | 1980 | M | M | Navy | 2,792 |
| 60 | AC Cruiser | HR93-1843 | OAC Laser/4/OAC  Gryphon/3/HR13-621//Haro Rico/XAN 159 | Canada | 2001 | M | M | Navy | 2,722 |
| 61 | Crestwood | GT0381 | Ex Rico 23*3/Ex Rico 23/PI 326418 | Canada | 1985 | M | M | Navy | 2,703 |
| 63 | EMP 419 | NA | NA | Colombia-CIAT | 1985 | M | M | Navy | 2,697 |
| 68 | OAC Speedvale | OAC 87-1 | Seafarer/PI 324685 | Canada | 1991 | M | M | Navy | 2,626 |
| 79 | OAC Laser | OAC 87-2 | Midnight/Seafarer | Canada | 1991 | M | M | Navy | 2,453 |
| 83 | Cornell 2114-12w | NA | NA | USA | 1985 | M | M | Navy | 2,404 |
| 84 | Corvette | NA | Robust x Hybrid (Wells Red Kidney/White Pea) | Canada | 1943 | M | M | Navy | 2,388 |
| 85 | AC Hensall | HR43-1582 | OAC Rico'/4/W1541-1503 F2  /3/W1336-F2//'OAC Seaforth'/I667 | Canada | 1995 | M | M | Navy | 2,356 |
| 86 | Centralia | HR10-685 | Ex Rico_23 BC6 F1-Are/Harokent | Canada | 1987 | M | M | Navy | 2,353 |
| 87 | Harofleet | Fleetwood *Are* | Fleetwood*6/Fleetwood/PI 326418 | Canada | 1983 | M | M | Navy | 2,335 |
| 90 | Sanilac | BC60 Are | Robust/Crawford//X-Ray mutant out of Michelite/3/X-Ray mutant out of Michelite/*2Michelite//Em 53 | USA | 1957 | M | M | Navy | 2,127 |
| 92 | Harokent | Kentwood *Are* | Kentwood*6/Kentwood/PI 326418 | Canada | 1983 | M | M | Navy | 2,122 |
| 95 | Saginaw | 874 | Mitchelite*6/US5Refugee//Robust/Crawford/3/Emerson 847 *6//Robust/Early Prolific | USA | 1963 | M | M | Navy | 2,071 |
| 97 | Michelite | NA | Robust/ Early Prolific | USA | 1938  (1963) | M | M | Navy | 2,040 |
| 101 | OAC Seaforth | Seafarer ARE | Seafarer*6/Seafarer/PI 326418 | Canada | 1983 | M | M | Navy | 1,971 |
| 104 | Seafarer | 0173 | Michelite X-ray mutant/3/Emerson/2/(Michelite/Trag)/(Michelite/Florida) | USA | 1967 | M | M | Navy | 1,899 |
| 109 | Midland | T7901 | Seafarer/2/Seafarer/California Small White | Canada | 1983 | M | M | Navy | 1,783 |
| 117 | OAC Spark | OAC 05-1 | OAC 97-1/OAC 99-1 | Canada | 2012 | M | M | Navy | 1,844 |
| 1 | Lighthouse | OAC 09-4 | spscbbr136/PI207262//ICB-17 10/Vax4///OAC Speedvale/Avanti//OAC 99-1/OAC Rex | Canada | 2012 | M | M | Navy (Pea) | 4,107 |
| 41 | SCX2098 | NA | Silvercreek/CIAT9 | Canada | 2005 | M | M | Small Red | 2,998 |
| 70 | Merlot | R98026 | ARS-R94037/ARS-R94161 | USA | 2004 | M | M | Small Red | 2,609 |
| 80 | 9438-140 | NA | NA | NA | 2002 | M | M | Small Red | 2,442 |
| 102 | OAC Rosito | ACUG 13-SR1 | NA | Canada | 2017 | M | M | Small Red | 1,945 |

^1^Entry order was based on time of inclusion in the AYD_AM panel

^2^Year of cultivar registration in Canada is shown in brackets (available at: http://www.inspection.gc.ca)

^3^Gene pool: M, Mesoamerican; A, Andean

^4^Race: Mesoamerican gene pool - D, Durango; M, Mesoamerica; Andean gene pool - NG, Nueva Granada

^5^Initial yield of selected germplasms (average of genotypes evaluated over five to seven year/location environments)

^6^NA, not available
