# Supplementary material for: Mapping yield and yield-related traits using diverse common bean germplasm
Source: Front Genet. 2024 Jan 3;14:1246904. doi: 10.3389/fgene.2023.1246904 (PMC10791882; doi:10.3389/fgene.2023.1246904)
Supplement: Supplementary file 4 [file Table8.DOCX]

**Supplementary Table S8 |** Genetic diversity of the AYD_AM collection of common bean genotypes. Diversity parameters (π per bp, θ per bp and Tajima’s D) were calculated with the 4,485 filtered SNPs in Tassel 5.

| **Type** | **π per bp** | | | | | **θ per bp** | | | | | **Tajima’s D** | | | | |
| --- | --- | --- | --- | --- | --- | --- | --- | --- | --- | --- | --- | --- | --- | --- | --- |
|  | **121^1^** | **Release year^2^** | | **Gene pool^3^** | | **121** | **Release year** | | **Gene pool** | | **121** | **Release year** | | **Gene pool** | |
|  |  | **Old** | **New** | **A** | **M** |  | **Old** | **New** | **A** | **M** |  | **Old** | **New** | **A** | **M** |
| **Genome** | **0.3866** | **0.3831** | **0.3881** | **0.1454** | **0.2460** | **0.1866** | **0.2233** | **0.2069** | **0.1433** | **0.1721** | **3.6031** | **2.6241** | **3.0898** | **0.0557** | **1.4864** |
| Pv01 | 0.4003 | 0.4008 | 0.3982 | 0.0858 | 0.2151 | 0.1864 | 0.2233 | 0.2069 | 0.1078 | 0.1855 | 3.7847 | 0.8713 | 3.2080 | -0.3876 | 0.5880 |
| Pv02 | 0.3980 | 0.3991 | 0.3973 | 0.0690 | 0.3017 | 0.1865 | 0.2233 | 0.2069 | 0.1290 | 0.1802 | 3.7397 | 2.8427 | 3.1914 | -1.6075 | 2.2605 |
| Pv03 | 0.3849 | 0.3887 | 0.3841 | 0.1646 | 0.2264 | 0.1865 | 0.2233 | 0.2070 | 0.1228 | 0.1626 | 3.5103 | 2.6741 | 2.9470 | 1.1481 | 1.2992 |
| Pv04 | 0.3905 | 0.4009 | 0.3832 | 0.1801 | 0.2555 | 0.1866 | 0.2234 | 0.2071 | 0.1669 | 0.1419 | 3.6065 | 2.8702 | 2.9510 | 0.1654 | 2.6752 |
| Pv05 | 0.4107 | 0.4024 | 0.4141 | 0.1932 | 0.3066 | 0.1866 | 0.2236 | 0.2069 | 0.1770 | 0.1917 | 3.9624 | 2.8892 | 3.4738 | 0.0334 | 2.0223 |
| Pv06 | 0.3912 | 0.3772 | 0.3973 | 0.0989 | 0.2926 | 0.1866 | 0.2233 | 0.2072 | 0.0795 | 0.1852 | 3.6162 | 2.4895 | 3.1853 | 0.8841 | 1.9570 |
| Pv07 | 0.3935 | 0.3933 | 0.3911 | 0.1026 | 0.2796 | 0.1866 | 0.2233 | 0.2070 | 0.0952 | 0.1920 | 3.6596 | 2.7492 | 3.0889 | 0.2909 | 1.5266 |
| Pv08 | 0.3635 | 0.3616 | 0.3629 | 0.1223 | 0.2218 | 0.1864 | 0.2233 | 0.2069 | 0.1285 | 0.1700 | 3.1317 | 2.2367 | 2.6159 | 0.2725 | 1.0357 |
| Pv09 | 0.3491 | 0.3387 | 0.3553 | 0.1856 | 0.1614 | 0.1866 | 0.2238 | 0.2071 | 0.1959 | 0.1387 | 2.8718 | 1.8561 | 2.4842 | -0.2262 | 0.5538 |
| Pv10 | 0.3671 | 0.3499 | 0.3795 | 0.1700 | 0.1706 | 0.1866 | 0.2233 | 0.2069 | 0.1824 | 0.1500 | 3.1931 | 2.0479 | 2.8928 | -0.2692 | 0.3867 |
| Pv11 | 0.3954 | 0.3807 | 0.4076 | 0.3197 | 0.2139 | 0.1865 | 0.2233 | 0.2069 | 0.2081 | 0.1956 | 3.6948 | 2.5463 | 3.3650 | 2.0208 | 0.3163 |

^1^121, YD_AM panel (121 genotypes)

^2^Release year, where old = 50 genotypes released in or before year 2000 and new = 71 genotypes released after year 2000

^3^Gene pool, where A = Andean (34 genotypes) and M = Mesoamerican (87 genotypes)
