# Supplementary material for: Mapping yield and yield-related traits using diverse common bean germplasm
Source: Front Genet. 2024 Jan 3;14:1246904. doi: 10.3389/fgene.2023.1246904 (PMC10791882; doi:10.3389/fgene.2023.1246904)
Supplement: Supplementary file 13 [file Table2.DOCX]

**Supplementary Table S2 |** Primer sequences and PCR conditions for SCAR, and gene-based NPP and AYD markers.

**Table S2a |** Yield/antiyield gene-based Temperature Switch PCR (TSP) markers tested in the 121 AYD_AM panel of common beans. [Source: Reinprecht, Y., Qi, Y., Shahmir, F., Smith, T. H. and Pauls, K. P. (2021)]. Yield and antiyield genes in common bean (*Phaseolus vulgaris* L.). *Legume Sci*., 1-17. https://doi.org/10.1002/leg3.91]

| **TSP marker** | **Primer** | | **Amplicon size (bp)** | **Tagged Locus** | **Genome location in**  ***Phaseolus vulgaris* v2.1** |
| --- | --- | --- | --- | --- | --- |
|  | **ID** | **Sequence (5’ – 3’)** |  |  |  |
| AYD1m | AYD1-GS-F | TCGCATAACAAACTTAACATTGGACT | 497/340_TSP,SNP_ | *Phvul.009G190100* | Pv09: 28,837,231 |
|  | AYD1-GS-R | TGTTGTTGTAAAAATGTAGAAGGTGGA |  |  |  |
|  | AYD1-AS-F | GGAAATATATACTTTAGATAAATTTTG |  |  |  |
| AYD2m | AYD2-GS-F | CCAGTGCATGACACGTGGAC | 255_TSP_/407_SNP_ | *Phvul.009G202100* | Pv09: 30,674,030 |
|  | AYD2-GS-R | CAGTACCCTTTTGGTTCCCTCA |  |  |  |
|  | AYD2-AS-F | ATCAACCATTTGCAAAAT |  |  |  |

“The TSP assays were performed in a 20-μl reaction volumes containing 1× PCR buffer and 3-mM MgCl2 (both supplied with enzyme), 0.1 mM each of dNTPs (Invitrogen), 125-ng bovine serum albumin Fraction V (Sigma-Aldrich), 0.6-U JumpStart Taq DNA Polymerase (Sigma-Aldrich), 0.05 μM each of the forward and reverse GS primers, 0.5-μM AS primer, and approximately 25 ng of common bean genomic DNA.

**Supplementary Table 2Sb |** PCR protocol used to amplify SCAR markers associated with CBB resistance in common bean AYD_AM panel.

| **Components** | **Stock concentration** | **20 μl reaction** | **Work**  **concentration** | **Supplier** |
| --- | --- | --- | --- | --- |
| H_2_O | - | 6.11 μl | - | MBG, Corning (46-000-CV) |
| PCR Buffer | 10x | 2.00 μl | 1x | Sigma (with Taq) |
| MgCl_2_ | 25 mM | 2.40 μl | 3 mM | Sigma (with Taq) |
| dNTPs | 0.5 mM | 4.00 μl | 0.1 mM | Invitrogen (10297-018) |
| BSA | 20 mg/ml | 0.25 μl | 5.0 μg/μl | New England Biolabs (NEB, B9000S) |
| GS primer F | 5 μM | 1.00 μl | 0.25 μM | UofG, Lab Services |
| GS primer R | 5 μM | 1.00 μl | 0.25 μM | UofG, Lab Services |
| AS primer F |  |  |  |  |
| JumpStart Taq | 2.5 U/μl | 0.24 μl | 0.576 U | Sigma (D9307-250UN) |
| Bean DNA | 8 ng/μl | 3.00 μl | 8 ng/μl | DNA from 121-line AM panel (extracted with Qiagen DNeasy Plant Mini Kit, 69104) |
| **OR** | | | | |
| PCR master mix | 2x | 10.00 μl | 1x | Invitrogen™ Platinum™ II Hot-Start Green PCR Master Mix/ Invitrogen™ 14001013 |
| GS primer F | 5 μM | 1.00 μl | 0.25 μM | UofG, Lab Services |
| GS primer R | 5 μM | 1.00 μl | 0.25 μM | UofG, Lab Services |
| AS primer F |  |  |  |  |
| H_2_O | - | 5.00 μl | - | MBG, Corning (46-000-CV) |
| Bean DNA | 8 ng/μl | 3.00 μl | 8 ng/μl | DNA from 121-line AM panel (extracted with Qiagen DNeasy Plant Mini Kit, 69104) |

**Supplementary Table S2c |** PCR cycling conditions used to amplify Yield/antiyield TSP markers in the AYD_AM panel of common beans. PCRs were performed in BioRad My Cycler with a PCR mixture listed in Table 2b. For each marker, annealing temperature was selected based on a gradient PCR. Hold at 4^o^C was included in PCR cycling for each marker.

| **Initial denaturation** | **Phase** | **Number of cycles of** | **Cycling (duration and temperature)** | | | **Final extension**  **at 72^o^C** |
| --- | --- | --- | --- | --- | --- | --- |
|  |  |  | **Denaturation** | **Annealing** | **Extension** |  |
| 94^o^C for 5 min | 1. Enrichment of GS product | 15 | 30s at 95^o^C | 30s at 58^o^C | 60s at 72^o^C | 10 min |
|  | 2. Incorporation of AS primer into enriched GS PCRs | 5 | 10s at 95^o^C | 30s at 45^o^C | - |  |
|  | 3.Competitive amplification of GS and AS PCR products | 15 | 10s at 95^o^C | 30s at 53^o^C | 5s at 72^o^C |  |

The TSP PCR products were separated on a 1% ultra-pure agarose (Invitrogen) gel (with ethidium bromide added) at 100 V in 1× TBE buffer for 1–2 h. The amplicons were visualized on ChemiDoc™ XRS system with the Image Lab™ software (BioRad).”

**Supplementary Table S2d |** Gene-based markers associated with CBB tested in the 121 AYD_AM panel of common beans.

[Source: Morneau, E. (2019). MSc thesis, University of Guelph]

| **Marker** | **Primer** | | **Amplicon size (bp)** | **Tagged Locus** | **Genome location in**  ***Phaseolus vulgaris* v2.1** |
| --- | --- | --- | --- | --- | --- |
|  | **ID** | **Sequence (5’ – 3’)** |  |  |  |
| NPP | G7-NPP | GCTTCTGTTGGTAGTTTGCAT  ATAGGAATCTCGTGGAAGAGC | 956_R_/535_S_ | SU91-QTL (Gene 7, Niemann Pick, *Phvul.008g291900*) | Pv08: 62,915,925 |
| - | G4-600 | ACAGATGATGAGACCACAAGTGA  AATTTTGTCCTCGGATCTCCTG | 602 | SU91 (Gene 4,  R gene 231733-8-004, KF429160) | Pv08:62,416,216 |
| - | G4-200 | AACCCAAGTTGAGCTTCCAGA  AATTTTGTCCTCGGATCTCCTG | 163 | SU91 (Gene 4,  R gene 231733-8-005, K429161F) | Pv08:62,461,659 |

**Supplementary Table S2e |** PCR protocol used to amplify gene-based markers associated with CBB resistance in common bean AYD_AM panel.

| **Components** | **Stock concentration** | **20 μl reaction** | **Work**  **concentration** | **Supplier** |
| --- | --- | --- | --- | --- |
| H_2_O | - | 6.11 μl | - | MBG, Corning (46-000-CV) |
| PCR Buffer | 10x | 2.00 μl | 1x | Sigma (with Taq) |
| MgCl_2_ | 25 mM | 2.40 μl | 3 mM | Sigma (with Taq) |
| dNTPs | 0.5 mM | 4.00 μl | 0.1 mM | Invitrogen (10297-018) |
| BSA | 20 mg/ml | 0.25 μl | 5.0 μg/μl | New England Biolabs (NEB, B9000S) |
| Primer F | 5 μM | 1.00 μl | 0.25 μM | UofG, Lab Services |
| Primer R | 5 μM | 1.00 μl | 0.25 μM | UofG, Lab Services |
| JumpStart Taq | 2.5 U/μl | 0.24 μl | 0.576 U | Sigma (D9307-250UN) |
| Bean DNA | 8 ng/μl | 3.00 μl | 8 ng/μl | DNA from 121-line AM panel (extracted with Qiagen DNeasy Plant Mini Kit, 69104) |
| **OR** | | | | |
| PCR master mix | 2x | 10.00 μl | 1x | Invitrogen™ Platinum™ II Hot-Start Green PCR Master Mix/ Invitrogen™ 14001013 |
| Primer F | 5 μM | 1.00 μl | 0.25 μM | UofG, Lab Services |
| Primer R | 5 μM | 1.00 μl | 0.25 μM | UofG, Lab Services |
| H_2_O | - | 5.00 μl | - | MBG, Corning (46-000-CV) |
| Bean DNA | 8 ng/μl | 3.00 μl | 8 ng/μl | DNA from 121-line AM panel (extracted with Qiagen DNeasy Plant Mini Kit, 69104) |

**Supplementary Table S2f |** PCR cycling conditions used to amplify gene-based markers associated withCBB resistance in common bean AYD_AM panel. PCRs were performed in BioRad My Cycler with a PCR mixture listed in Table 2b. For each marker, annealing temperature was selected based on a gradient PCR. Hold at 4^o^C was included in PCR cycling for each marker.

| **Marker** | **Initial denaturation** | **Number of**  **cycles of** | **Cycling (duration and temperature)** | | | **Final extension**  **at 72^o^C** | **PCR product (bp)** | **PCR**  **'quality'** |
| --- | --- | --- | --- | --- | --- | --- | --- | --- |
|  |  |  | **Denaturation** | **Annealing** | **Extension (72^o^C)** |  |  |  |
| NPP (G7) | 94^o^C for 3 min | 35 | 30s at 94^o^C | 45s at 65^o^C | 60s | 10 min | 956/535 | √ |
| G4-600 | 94^o^C for 3 min | 35 | 30s at 94^o^C | 45s at 65^o^C | 60s | 10 min | 602 | √ |
| G4-200 | 94^o^C for 3 min | 35 | 30s at 94^o^C | 45s at 65^o^C | 60s | 10 min | 162 | √ |

**Supplementary Table S2g |** SCAR markers associated with CBB resistance in common bean (*Phaseolus vulgaris* L.) tested in the AYD_AM panel [Source: Miklas. P. (2005). DNA markers (SCARS) linked with disease resistance traits in bean (*Phaseolus vulgaris*). Updated: 1/20/05 by Phil Miklas. Available at: https://www.ars.usda.gov/ARSUserFiles/3848/PDF/Scartable3.pdf]

| **SCAR** | **Size (bp)** | **Primers (5’ – 3’)** | **Tagged Locus** | **Chr.** | **Reference** | **Genome location in**  ***Phaseolus vulgaris* v2.1** |
| --- | --- | --- | --- | --- | --- | --- |
| SW13^1^ | 690 | CACAGCGACATTAATTTTCCTTTC  CACAGCGACAGGAGGAGCTTATTA | *I* locus (BCMV)  *Pse-3* locus (HB) | Pv02 | Haley et al., 1994  Melotto et al., 1996 | Pv02: 36,923,563 |
| R4865 | 950 | TCCAAAGCCATTCTAGTT  CAGCTACTTTCAAAC | Major QTL  (OAC 88-1) | Pv03 | Bai et al., 1997  Beattie et al., 1998 | Pv03: 41,208,322 |
| BC420 | 900 | GCAGGGTTCGAAGACACACTGG  GCAGGGTTCGCCCAATAACG | Major QTL  (XAN 159) | Pv06 | Yu et al., 2000 | Pv06: 20,457,061 |
| BC420-CG14 | 519/425 | CGAGACTCGTGTGCTCTCTG  ACGAAGGTTGATTCCCAGTG | Major QTL | Pv06 | Shi et al., 2012 | Pv06: 5,736,339 |
| BC420-CG10B | 459 | CCACCTGCCACATAGACCTT  TCTCGAGAAGGGCAGAGGTA | Major QTL | Pv06 | Shi et al., 2012 |  |
| BC420-CG9 | 451/350 | AAGCAAACCCTTCCATTCC  TCCCAAACACCAATGGAAAT | Major QTL | Pv06 | Shi et al., 2012 |  |
| STS183 | 142 | CCTATGTACTTCTTGAGGGAGAC  AGAAGCCCAGGGACTTGGAT | - | Pv06 | Liu et al., 2008 |  |
| STS333a | 274 | CATAAGATGAATGGTTCTTGAC  CCATTTGGTGAGATTCACTT | - | Pv06 | Liu et al., 2008 |  |
| Phs^2^ | Multiple | AGCATATTCTAGAGGCCTCC  GCTCAGTTCCTCAATCTGTTC | Major QTL (WM7.1) (G 122) & (BAT 93) | Pv07 | Kami et al., 1995  Nodari et al., 1993 Miklas et al., 2001 | Pv07: 4,959,794 |
| SAS13^3^ | 950 | CACGGACCGAATAAGCCACCAACA  CACGGACCGAGGATACAGTGAAAG | Co-4^2^ | Pv08 | Young et al., 1998  Kelly et al., 2003 | Pv08: 2,282,250 |
| R7313 | 700 | ATTGTTATCGTCGACACG  AATATTTCTGATCACACGAG | Major QTL  (OAC 88-1) | Pv08 | Bai et al., 1997  Beattie et al., 1998 | Pv08: 57,179,804 |
| SU91 | 700 | CCACATCGGTTAACATGAGT  CCACATCGGTGTCAACGTGA | Major QTL  (XAN 159) | Pv08 | Pedraza et al., 1997 | Pv08:62,837,460 |
| SU91-CG11 | 464/425 | GGCGACGGCTTCTTTGAC  TCCAAAGACCAAAGGGTGAG | Major QTL | Pv08 | Shi et al., 2012 | Pv08: 59,338,805 |
| SU91-CG10 | 425/350 | ATGGTGGAGACGAGATGACC  TCCGACATTGAAACCAGTTG | Major QTL | Pv08 | Shi et al., 2012 | Pv08: 62,815,310 |
| BAC6 | 1250 | TAGGCGGCGGCGCACGTTTTG  TAGGCGGCGGAAGTGGCGGTG | Major QTL  (GN#1 sel 27) | Pv10 | Jung et al., 1999 | Pv10: 22,159,094 |
| SAP6 | 820 | GTCACGTCTCCTTAATAGTA  GTCACGTCTCAATAGGCAAA | Major QTL  (GN#1 sel 27) | Pv10 | Miklas et al., 2000 | Pv10: 41,048,879 |

^1^SW13, pathogens BCMV/CBB

^2^Phs, pathogens CBB/WM

^3^SAS13, pathogens CBB/ANT

**Supplementary Table S2h |** PCR protocol used to amplify SCAR markers associated with CBB resistance in common bean AYD_AM panel.

| **Components** | **Stock concentration** | **20 μl reaction** | **Work**  **concentration** | **Supplier** |
| --- | --- | --- | --- | --- |
| H_2_O | - | 6.11 μl | - | MBG, Corning (46-000-CV) |
| PCR Buffer | 10x | 2.00 μl | 1x | Sigma (with Taq) |
| MgCl_2_ | 25 mM | 2.40 μl | 3 mM | Sigma (with Taq) |
| dNTPs | 0.5 mM | 4.00 μl | 0.1 mM | Invitrogen (10297-018) |
| BSA | 20 mg/ml | 0.25 μl | 5.0 μg/μl | New England Biolabs (NEB, B9000S) |
| Primer F | 5 μM | 1.00 μl | 0.25 μM | UofG, Lab Services |
| Primer R | 5 μM | 1.00 μl | 0.25 μM | UofG, Lab Services |
| JumpStart Taq | 2.5 U/μl | 0.24 μl | 0.576 U | Sigma (D9307-250UN) |
| Bean DNA | 8 ng/μl | 3.00 μl | 8 ng/μl | DNA from 121-line AM panel (extracted with Qiagen DNeasy Plant Mini Kit, 69104) |
| **OR** | | | | |
| PCR master mix | 2x | 10.00 μl | 1x | Invitrogen™ Platinum™ II Hot-Start Green PCR Master Mix/ Invitrogen™ 14001013 |
| Primer F | 5 μM | 1.00 μl | 0.25 μM | UofG, Lab Services |
| Primer R | 5 μM | 1.00 μl | 0.25 μM | UofG, Lab Services |
| H_2_O | - | 5.00 μl | - | MBG, Corning (46-000-CV) |
| Bean DNA | 8 ng/μl | 3.00 μl | 8 ng/μl | DNA from 121-line AM panel (extracted with Qiagen DNeasy Plant Mini Kit, 69104) |

**Supplementary Table 2i |** PCR cycling conditions used to amplify markers associated with CBB resistance in common bean AYD_AM panel. PCRs were performed in BioRad My Cycler with a PCR mixture listed in Table 2b. For each marker, annealing temperature was selected based on a gradient PCR. Hold at 4^o^C was included in PCR cycling for each marker.

| **Marker** | **Initial denaturation** | **Number of**  **cycles of** | **Cycling (duration and temperature)** | | | **Final extension**  **at 72^o^C** | **PCR product (bp)** | **PCR**  **'quality'** |
| --- | --- | --- | --- | --- | --- | --- | --- | --- |
|  |  |  | **Denaturation** | **Annealing** | **Extension (72^o^C)** |  |  |  |
| SW13 | 94^o^C for 3 min | 35 | 30s at 94^o^C | 45s at 67^o^C | 60s | 10 min | 690 | √ |
| R4865 | 95^o^C for 2 min | 35 | 45s at 95^o^C | 40s at 50oC | 90s | 5 min | 950 | √ |
| BC420 | 94^o^C for 3 min | 35 | 30s at 94^o^C | 45s at 60^o^C | 60s | 10 min | 900 | √ |
| BC420-CG14 | 94^o^C for 3 min | 35 | 30s at 94^o^C | 45s at 60^o^C | 60s | 10 min | 519/425 | √ |
| BC420-CG10B | 94^o^C for 3 min | 35 | 30s at 94^o^C | 45s at 60^o^C | 60s | 10 min | 459 | - |
| BC420-CG9 | 95^o^C for 2min | 35 | 30s at 94^o^C | 45s at 60^o^C | 60s | 5 min | 415/375 | - |
| STS183 | 94^o^C for 3 min | 35 | 30s at 94^o^C | 45s at 62^o^C | 60s | 10 min | 142 | √ |
| STS333a | 94^o^C for 3 min | 35 | 30s at 94^o^C | 45s at 62^o^C | 60s | 10 min | 274 | √ |
| Phs | - | 34 | 10s at 94^o^C | 40s at 50^o^C | 120s | 5 min | Multiple | - |
| SAS13 | 94^o^C for 3 min | 35 | 30s at 94^o^C | 60s at 68^o^C | 90s | 10 min | 950 | √ |
| R7313 | - | 34 | 10s at 94^o^C | 40s at 60^o^C | 120s | 5 min | 700 | - |
| SU91 | 94^o^C for 3 min | 35 | 30s at 94^o^C | 45s at 65^o^C | 60s | 10 min | 700 | √ |
| SU91-CG11 | 94^o^C for 3 min | 35 | 30s at 94^o^C | 45s at 65^o^C | 60s | 10 min | 464/425 | √ |
| SU91-CG10 | 94^o^C for 3 min | 35 | 30s at 94^o^C | 45s at 60^o^C | 60s | 10 min | 425/350 | √ |
| BAC6 | 94^o^C for 3 min | 35 | 30s at 94^o^C | 60s at 68^o^C | 90s | 10 min | 1250 | √ |
| SAP6 | 95^o^C for 4 min | 34 | 10s at 94^o^C | 40s at 55^o^C | 120s | 5 min | 820 | √ |
