# Supplementary material for: Mapping yield and yield-related traits using diverse common bean germplasm
Source: Front Genet. 2024 Jan 3;14:1246904. doi: 10.3389/fgene.2023.1246904 (PMC10791882; doi:10.3389/fgene.2023.1246904)
Supplement: Supplementary file 15 [file Table5.DOCX]

**Supplementary Table S5 |** Broad-sense mean-based heritability for traits analyzed in the AYD_AM collection of common beans in four Ontario location/year environments.

| **Trait^1^** | **Environment^2^** | | | | | | **Combined** |
| --- | --- | --- | --- | --- | --- | --- | --- |
|  | **ERS15** | **WRS15** | **HAR15** | **ERS16** | **WRS16** | **HAR16** |  |
| YD | 0.7822 | 0.7523 | - | 0.9001 | 0.8159 | - | 0.7723 |
| SW | 0.9987 | 0.9967 | - | 0.9981 | 0.9974 | - | 0.9826 |
| DF | 0.9726 | 0.9848 | - | 0.9724 | 0.9716 | - | 0.9588 |
| DM | 0.9622 | 0.9575 | - | 0.9554 | 0.9668 | - | 0.9392 |
| PH | 0.8869 | 0.8535 | - | 0.8269 | 0.8586 | - | 0.8549 |
| HR | - | - | - | 0.8941 | 0.7538 | - | 0.6867 |
| RP | 0.9513 | 0.9244 | - | 0.9230 | 0.9315 | - | 0.8848 |
| YGD | 0.7523 | 0.7175 | - | 0.8742 | 0.7722 | - | 0.6623 |
| SGR | 0.7892 | 0.7240 | - | 0.9000 | 0.7982 | - | 0.6419 |
| YDH | 0.7277 | 0.6360 | - | 0.8546 | 0.7425 | - | 0.5383 |
| SN | 0.9326 | 0.9069 | - | 0.9666 | 0.9574 | - | 0.9265 |
| YDHR | - | - | - | 0.8820 | 0.7958 | - | 0.6668 |
| CBB_R1 | - | - | 0.7287 | - | - | 0.4902 | 0.4720 |
| CBB_R2 | - | - | 0.8315 | - | - | 0.6151 | 0.7490 |
| CBB_AUDPC | - | - | 0.8281 | - | - | 0.6494 | 0.6990 |

^1^Measured agronomic traits (ERS and WRS in 2015 and 2016): YD, yield (kg ha^-1^); SW, seed weight (g); DF, flowering (days); DM, maturity (days); PH, plant height (cm); harvestability (1-5 scale, data collected only in 2016); Derived traits: RP, reproductive period [RP = DM – DF (days)]; YGD, yield gain per day [YGD = YD / DM (kg day^-1^ ha^-1^); SGR, seed growth rate [SGR = YD / RP (kg ha^-1^ day^-1^)]; YDH, yield per unit of height [YDH = YD / PH (kg ha^-1^ cm^-1^)]; SN, seed number [SN = YD / SW (seed number x 10^6^ seeds ha^-1^)]; YDHR, yield per unit of harvestability (YDHR = YD / HR); Disease resistance (AAFC, Harrow 2015 and 2016 disease nursery): CBB (common bacterial blight), where CBB_R1 indicated 1^st^ disease severity scoring (10 days after the inoculation), CBB_R2 denotes 2^nd^ disease severity scoring (10 days after the first scoring) and CBB_AUDPC, represent the area under disease progress curve (AUDPC) calculated based on two disease scorings using a scale 0-5.

^2^Environment: ERS15, Elora research station (ERS) 2015; ERS16, ERS 2016; WRS15, Woodstock research station (WRS) 2015; WRS16, WRS 2016; HAR15, disease nursery Harrow Research and Development Centre (HAR) 2015; HAR16, HAR 2016.
