# Supplementary material for: Mapping yield and yield-related traits using diverse common bean germplasm
Source: Front Genet. 2024 Jan 3;14:1246904. doi: 10.3389/fgene.2023.1246904 (PMC10791882; doi:10.3389/fgene.2023.1246904)
Supplement: Supplementary file 17 [file Table3.DOCX]

**Supplementary Table S3 |** Restricted maximum likelihood analysis of the field experiments conducted with 121 common bean genotypes at the Elora and Woodstock research stations (ON, Canada) in 2015 and 2016.

| **Traits^1^** | **Genotype (G) - Fixed effects**  **(type III tests of fixed effects Pr> F)** | | | | **Random effects (tests of covariance parameters based on the restricted likelihood, Pr > ChiSq)** | | | | **Shapiro-Wilk tests of normality** | |
| --- | --- | --- | --- | --- | --- | --- | --- | --- | --- | --- |
|  | **Numerator DF** | **Denominator DF** | **F value** | **Pr > F** | **Environment**  **(E)** | **G x E** | **Block** | **iBlock** | **Statistics (W)** | ***p* value**  **Pr < W** |
| YD | 120 | 354.70 | 4.24 | < 0.0001 | 0.0086 | < 0.0001 | < 0.0001 | < 0.0001 | 0.9985 | 0.0922 |
| SW | 120 | 358.00 | 57.08 | < 0.0001 | **< 0.0001** | < 0.0001 | 0.0001 | 0.0377 | 0.9919 | **< 0.0001** |
| DF | 120 | 353.00 | 23.76 | < 0.0001 | **< 0.0001** | < 0.0001 | < 0.0001 | < 0.0001 | 0.9946 | **< 0.0001** |
| DM | 120 | 352.50 | 16.11 | < 0.0001 | **< 0.0001** | < 0.0001 | < 0.0001 | < 0.0001 | 0.9978 | **0.0111** |
| PH | 120 | 356.60 | 6.71 | < 0.0001 | **< 0.0001** | < 0.0001 | < 0.0001 | < 0.0001 | 0.9990 | 0.3987 |
| HR | 120 | 119.30 | 3.15 | < 0.0001 | 0.1060 | < 0.0001 | < 0.0001 | 0.0981 | 0.9988 | 0.7892 |
| RP | 120 | 351.60 | 8.51 | < 0.0001 | **< 0.0001** | < 0.0001 | < 0.0001 | < 0.0001 | 0.9987 | 0.1753 |
| YGD | 120 | 356.50 | 2.87 | < 0.0001 | **0.0267** | < 0.0001 | < 0.0001 | < 0.0001 | 0.9992 | 0.5988 |
| SGR | 120 | 358.20 | 2.71 | < 0.0001 | **0.0100** | < 0.0001 | < 0.0001 | < 0.0001 | 0.9992 | 0.6429 |
| YDH | 120 | 357.60 | 2.11 | < 0.0001 | **< 0.0001** | < 0.0001 | < 0.0001 | < 0.0001 | 0.9919 | **< 0.0001** |
| SN | 120 | 355.40 | 12.93 | < 0.0001 | 0.0708 | < 0.0001 | < 0.0001 | < 0.0001 | 0.9960 | **0.0001** |
| YDHR | 120 | 114.30 | 2.93 | < 0.0001 | 0.3185 | < 0.0001 | < 0.0001 | < 0.0001 | 0.9900 | **< 0.0001** |
| CBB_R1 | 120 | 112.70 | 1.82 | 0.0007 | **0.0107** | 0.0001 | 0.6410 | NA | 0.9950 | 0.1533 |
| CBB_R2 | 120 | 98.65 | 3.80 | < 0.0001 | 0.0561 | 0.0132 | < 0.0001 | NA | 0.9939 | 0.0663 |
| CBB_AUDPC | 120 | 109.00 | 3.18 | < 0.0001 | 0.4838 | 0.0019 | < 0.0001 | NA | 0.9961 | 0.3419 |

^1^Measured agronomic traits (ERS and WRS in 2015 and 2016): YD, yield (kg ha^-1^); SW, seed weight (g); DF, flowering (days); DM, maturity (days); PH, plant height (cm); harvestability (1-5 scale, data collected only in 2016); Derived traits: RP, reproductive period [RP = DM – DF (days)]; YGD, yield gain per day [YGD = YD / DM (kg day^-1^ ha^-1^); SGR, seed growth rate [SGR = YD / RP (kg ha^-1^ day^-1^)]; YDH, yield per unit of height [YDH = YD / PH (kg ha^-1^ cm^-1^)]; SN, seed number [SN = YD / SW (seed number x 10^6^ seeds ha^-1^)]; YDHR, yield per unit of harvestability (YDHR = YD / HR); Disease resistance (AAFC, Harrow 2015 and 2016 disease nursery): CBB (common bacterial blight), where CBB_R1 indicated 1^st^ disease severity scoring (10 days after the inoculation), CBB_R2 denotes 2^nd^ disease severity scoring (10 days after the first scoring) and CBB_AUDPC, represent the area under disease progress curve (AUDPC) calculated based on two disease scorings using a scale 0-5.
