# Supplementary material for: Mapping yield and yield-related traits using diverse common bean germplasm
Source: Front Genet. 2024 Jan 3;14:1246904. doi: 10.3389/fgene.2023.1246904 (PMC10791882; doi:10.3389/fgene.2023.1246904)
Supplement: Supplementary file 20 [file Table10.DOCX]

**Supplementary Table S10 |** Marker trait correlation (Spearman Correlation Coefficients; Prob > |r| under H0: Rho=0; Number of observations)

| **Marker^1^** | **Trait^2^** | | | | | | | | | | | | | | | **Marker** | |
| --- | --- | --- | --- | --- | --- | --- | --- | --- | --- | --- | --- | --- | --- | --- | --- | --- | --- |
|  | **Measured agronomic traits** | | | | | | **Derived traits** | | | | | | **Disease resistance** | | |  |  |
|  | **YD** | **SW** | **DF** | **DM** | **PH** | **HR** | **RP** | **YGD** | **SGR** | **YDH** | **SN** | **YDHR** | **CBBR1** | **CBBR2** | **CBB_AUDPC** | **AYD2m** | **NPP** |
| **AYD1m** | .371 | -.681 | .546 | .417 | .218 | .311 | .157 | .285 | .387 | .429 | .700 | .003 | -.204 | -.258 | -.229 | -.877 | .247 |
|  | <.0001 | <.0001 | <.0001 | <.0001 | .0177 | .0006 | .0890 | .0017 | <.0001 | <.0001 | <.0001 | .9768 | .0264 | .0049 | .0126 | <.0001 | .0076 |
|  | 118 | 118 | 118 | 118 | 118 | 118 | 118 | 118 | 118 | 118 | 118 | 118 | 118 | 118 | 118 | 113 | 116 |
| **AYD2m** | -.292 | .739 | -.581 | -0.402 | -.148 | -.409 | -.082 | -.199 | -.340 | -.385 | -.690 | -.001 | .153 | .206 | .170 |  | -.236 |
|  | .0017 | <.0001 | <.0001 | <.0001 | .1180 | <.0001 | .3900 | .0343 | .0002 | <.0001 | <.0001 | .9901 | .1059 | .0288 | .0722 | 1 | .0122 |
|  | 113 | 113 | 113 | 113 | 113 | 113 | 113 | 113 | 113 | 113 | 113 | 113 | 13 | 113 | 113 |  | 112 |
| **NPP** | .118 | -.119 | .017 | 0.1853 | .170 | -.040 | .253 | .078 | .035 | .032 | .202 | .129 | -.453 | -0.517 | -.493 |  |  |
|  | .2030 | .1991 | .8513 | 0.0436 | .0651 | .6626 | .0055 | .4003 | .7050 | .7320 | .0273 | .01624 | <.0001 | <.0001 | <.0001 | 1 | 1 |
|  | 119 | 119 | 119 | 119 | 119 | 119 | 119 | 119 | 119 | 119 | 119 | 119 | 119 | 119 | 119 |  |  |

^1^Markers: AYD1m (AYD gene 1, *Phvul.009G190100*) and AYD2m (AYD gene 2, *Phvul.009G202100*), yield/anti-yield markers (Reinprecht et al., 2021); NPP (gene *Phvul.08G291900*) the Niemann-Pick polymorphism (NPP) CBB marker (Morneau, 2019).

^2^Measured agronomic traits (ERS and WRS in 2015 and 2016): YD, yield (kg ha^-1^); SW, seed weight (g); DF, flowering (days); DM, maturity (days); PH, plant height (cm); harvestability (1-5 scale, data collected only in 2016); Derived traits: RP, reproductive period [RP = DM – DF (days)]; YGD, yield gain per day [YGD = YD / DM (kg day^-1^ ha^-1^); SGR, seed growth rate [SGR = YD / RP (kg ha^-1^ day^-1^)]; YDH, yield per unit of height [YDH = YD / PH (kg ha^-1^ cm^-1^)]; SN, seed number [SN = YD / SW (seed number x 10^6^ seeds ha^-1^)]; YDHR, yield per unit of harvestability (YDHR = YD / HR); Disease resistance (AAFC, Harrow 2015 and 2016 disease nursery): CBB (common bacterial blight), where CBB_R1 indicated 1^st^ disease severity scoring (10 days after the inoculation), CBB_R2 denotes 2^nd^ disease severity scoring (10 days after the first scoring) and CBB_AUDPC, represent the area under disease progress curve (AUDPC) calculated based on two disease scorings using a scale 0-5.
